# A transaminase-mediated aldol reaction and applications in cascades to styryl pyridines†

**DOI:** 10.1039/d3cy01370g

**Published:** 2024-03-01

**Authors:** Yu Wang, Yiwen Li, Yeke Ni, Dejan-Krešimir Bučar, Paul A. Dalby, John M. Ward, Jack W. E. Jeffries, Helen C. Hailes

**Affiliations:** a Department of Chemistry, University College London 20 Gordon Street London WC1H 0AJ UK h.c.hailes@ucl.ac.uk; b Department of Biochemical Engineering, University College London Gower Street London WC1E 6BT UK jack.jeffries.12@ucl.ac.uk

## Abstract

Transaminase enzymes are well established biocatalysts that are used in chemical synthesis due to their beneficial sustainability profile, regio- and stereoselectivity and substrate specificity. Here, the use of a wild-type *Chromobacterium violaceum* transaminase (*Cv*TAm) in enzyme cascades revealed the formation of a novel hydroxystyryl pyridine product. Subsequent studies established it was a transaminase mediated reaction where it was exhibiting apparent aldolase reactivity. This promiscuous enzyme reaction mechanism was then explored using other wild-type transaminases and *via* the formation of *Cv*TAm mutants. Application of one pot multi-step enzyme cascades was subsequently developed to produce a range of hydroxystyryl pyridines.

## Abstract 2

A transaminase mediated aldolase reactivity has been discovered and used to construct styryl pyridines *via* the use of enzyme cascades.

## Graphical abstract

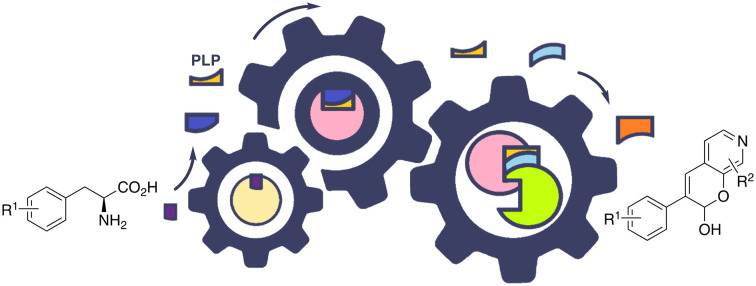

## Introduction

Biocatalysis has become an important aspect of modern organic synthesis, both in academia and across the chemical and pharmaceutical industries. Its success has been largely due to a rapid expansion of the range of chemical reactions accessible and substrates accepted. Applications of naturally occurring enzymes, however, can be limited by narrow substrate specificities. Some classes of enzymes do show good native enzyme substrate promiscuity where the same transformation is performed and is due to active site plasticity or alternative substrate binding modes. Recently, interest has also focused on discovering functional enzyme promiscuity where enzymes can additionally catalyse a non-physiological reaction.^1^ Examples include hydrolytic enzymes such as porcine pancreatic lipase and a peptidase from *Sulfolobus tokodaii* that have been reported to catalyse aldol reactions.^1–4^ Enzyme mutagenesis has been reported to modify enzyme functionality and an early example was a single mutation in alanine racemase which converted it into an aldolase.^5,6^ More recently, a range of new to nature enzyme functionalities have been reported such as photoinduced enzyme catalysis with ene reductases and variants *via* radical mechanisms for asymmetric reduction, hydroalkylation and conjugate addition.^7–9^ Enzyme engineering to produce other new enzyme functionalities such as efficient Morita–Baylis–Hillman and cyclopropanes have also been described, highlighting the potential of new biocatalysts for use in synthesis.^10–12^

Transaminases (TAms) are well established biocatalysts that reversibly transform a ketone or aldehyde group into an amine moiety using an amine donor and the co-factor pyridoxal 5′-phosphate (PLP) 1.^13,14^ When using prochiral ketones, the products can be single enantiomers and TAms have been used in numerous applications towards single isomer pharmaceutical ingredients, biologically active compounds, and small molecule amines not readily accessible *via* traditional synthetic routes.^15–20^

**Scheme 1:**
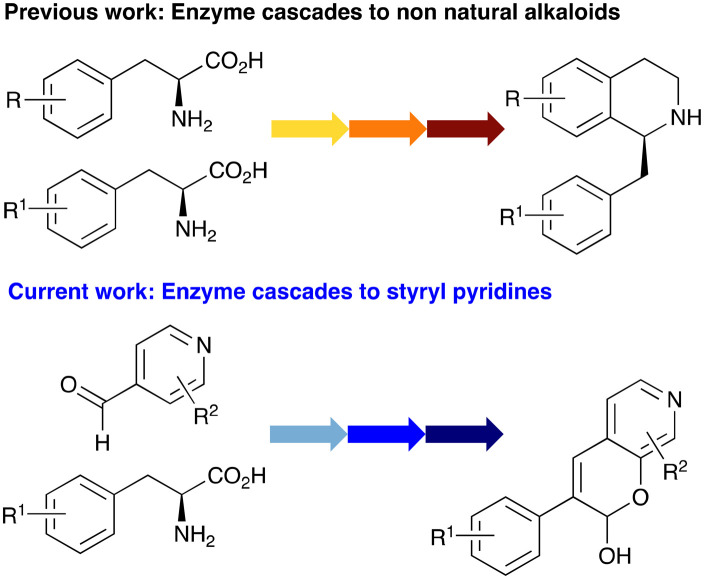
Previous work using cascades to alkaloids and novel aldolase activity mediated by *Cv*TAm in this work giving rise to styryl pyridines.

In this study, while incorporating the transaminase from *Chromobacterium violaceum* (*Cv*TAm)^21^ into biocatalytic cascades to give benzylisoquinoline alkaloids (BIAs), a novel product was identified, arising from one of the aldehydes formed *in situ* from a *Cv*TAm reaction and subsequent aldol condensation. Investigations revealed that *Cv*TAm appeared to catalyse the aldol reaction, and this was explored further *via in silico* enzyme modelling and the production of *Cv*TAm variants. Applications of this new *Cv*TAm functionality were then applied in multistep one-pot enzyme cascades incorporating a tyrosinase (TYR), tyrosine decarboxylase (TyrDC) and *Cv*TAm to generate complex styryl pyridines (Scheme 1), which are a promising class of molecules for several biological targets.

## Results and discussion

### Initial hydroxystyryl pyridine formation

In previous work one-pot enzyme cascades were developed to generate new BIAs, starting from l-tyrosine 2a and analogues. Several recombinant enzymes in *E. coli* were used, a tyrosinase from *Candidatus Nitrosopumilus salaria* BD31 (*Cn*TYR), tyrosine decarboxylase from *Enterococcus faecalis* DC32 (*Ef*TyrDC), *Cv*TAm and *Thalictrum flavum* norcoclaurine synthase (*Tf*NCS) to produce, *in vitro*, alkaloids such as norlaudanosoline in high yields and stereoselectivities.^22,23^ In subsequent work, parallel cascades were constructed using *Cn*TYR and variants together with a *Ef*TyrDC to produce the arylethylamine component, and *Ef*TyrDC and *Cv*TAm to produce arylacetaldehydes, enabling the use of two different amino acid starting materials. Addition of *Tf*NCS was then able to generate novel doubly halogenated BIAs.^24^ When generating arylacetaldehydes *in situ* from 2a and analogues, it was noted that an unknown side-product 3a was formed. While these could readily be separated from the BIA products, we were intrigued to understand how they were formed to streamline future cascade design.

**Scheme 2:**
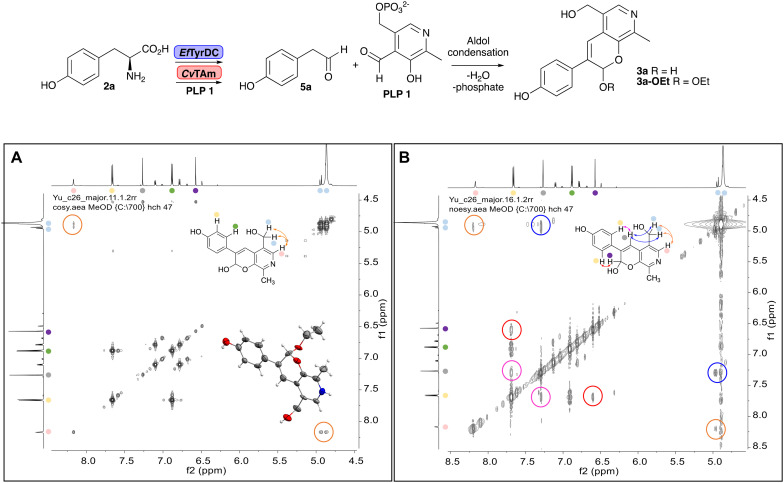
Formation of 3a and key characterisation data. A. COSY NMR spectra of 3a with a key long-range coupling indicated with double headed arrows and assignments using coloured circles. Also, the molecular structure of the 3a**-OEt** trifluoroacetate cation, as derived from single crystal X-ray diffraction analyses; B NOESY NMR spectra of 3a showing key NOEs with double headed arrows, and proton assignments.

NMR spectroscopic analysis confirmed that the new product 3a, formed when using 2a, was not a BIA with the characteristic tetrahydroisoquinoline signals, ruling out the involvement of *Tf*NCS. The presence of a deshielded proton at ∼8.2 ppm suggested that it could be a pyridinium species arising from 1. *Ortho*-aryl couplings indicated that hydroxylation using *Cn*TYR had not occurred, and that intermediates present including tyramine 4a, formed by an *in situ* decarboxylation with *Ef*TyrDC, or 4-hydroxylphenylacetaldehyde 5a generated by a subsequent reaction with *Cv*TAm, may be involved. Accurate mass spectrometry (MS) data (*m*/*z* 286.1072) corresponded to the addition of 1 and 5a and loss of H_2_O (and hydrolysis of the phosphate group), suggesting an aldol addition. Analysis of the NMR data indicted key NOEs between a CH_2_ group and alkene CH, the later also giving rise to an NOE with the aryl group (Scheme 2). Together, this suggested that 3a was 5-(hydroxymethyl)-3-(4-hydroxyphenyl)-8-methyl-2*H*-pyrano[2,3-*c*]pyridin-2-ol, and this was consistent with results of single crystal X-ray diffraction studies of a trifluoroacetate salt of **3a-OEt** – an ethoxy hemiacetal of 3a obtained through recrystallization from ethanol.

Recent studies have reported that some (*E*)-4-(substituted styryl)pyridines, structurally related to resveratrol, inhibited the formation of the vascular endothelial growth factor (VEGF) from HT-29 cells and expression of the telomerase-related *hTERT* and *c-Myc* genes.^25^ Other hydroxystyryl pyridines have been determined to act as aldose reductase inhibitors for type II diabetes applications.^26^ The hydroxystyryl pyridine 3a was therefore a potentially interesting pharmacophore, and also due to the unusual mode of formation the reaction was explored in more detail to give a range of styryl pyridines.

### Exploring the reaction conditions required for styryl pyridine formation

To establish whether the aldol addition to produce 3a was enzymatically catalysed or due to the presence of particular buffers, phenylacetaldehyde 5b and 1 were initially reacted together in different buffers or water (Table 1a), including 2-[4-(2-hydroxyethyl)piperazin-1-yl]ethanesulfonic acid (HEPES) buffer (50 mM, pH 5.5 and pH 7.5), potassium phosphate (KPi) buffer (300 mM, pH 5.5 and pH 7.5) and 2-amino-2-hydroxymethyl-propane-1,3-diol (Tris) buffer (100 mM, pH 5.5 and pH 7.5). While all reactions gave rise to three new peaks (P_1_–P_3_) (P_4_ is 5b) after 16 h by HPLC analysis, they were mixtures of uncharacterized side products (potentially formed *via* the enol of 5b) confirming that the formation of the styryl pyridine 3b was enzyme mediated.

**Table 1:** Exploring the reaction conditions for the aldol addition to give styryl pyridine 3b

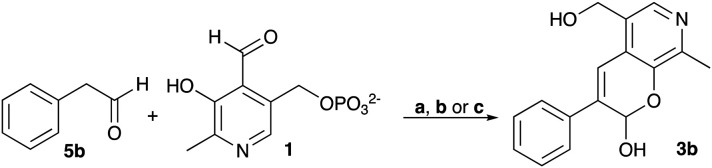
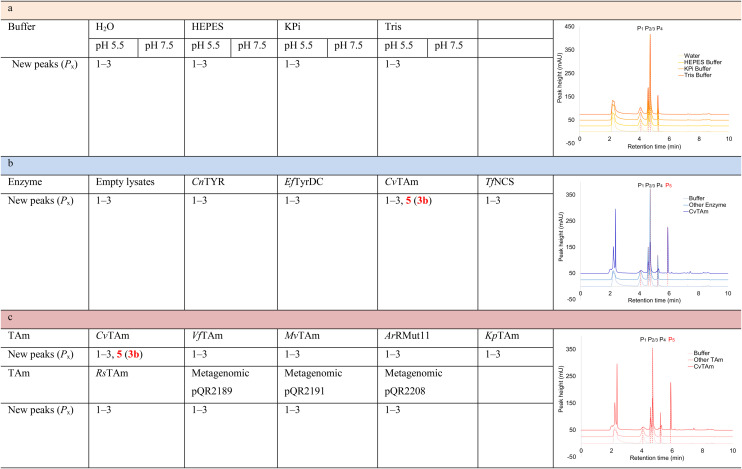

**Fig. 1:**
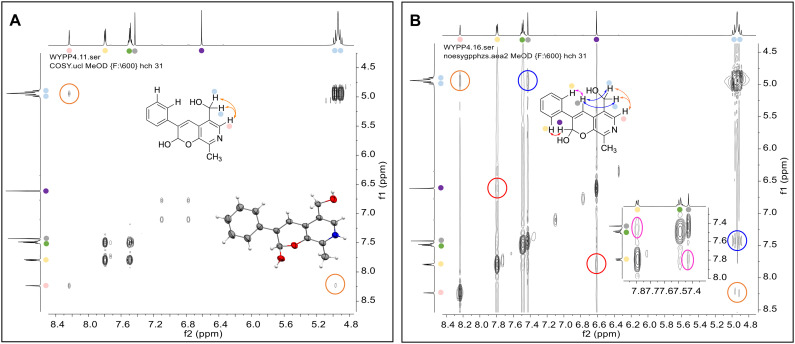
Key characterisation data for 3b. A. COSY NMR spectra of 3b with a key long-range coupling indicated with the double headed arrows and assignments using coloured circles. Also, the molecular structure of the 3b trifluoroacetate cation, as derived from single crystal X-ray diffraction analyses;† B NOESY NMR spectra of 3b showing key NOEs with double headed arrows, and proton assignments.

Enzymes used in the original enzyme cascade were then investigated including an empty cell vector lysate, lysates of *Cn*TYR, *Ef*TyrDC, *Cv*TAm and *Tf*NCS. In agreement with the preliminary observations, *Cn*TYR and *Tf*NCS were not involved in the styryl pyridine formation, giving rise to peaks P_1_–P_4_ only, and this was also the case for *Ef*TyrDC. However, the reaction with *Cv*TAm gave a new product (P_5_, Table 1b) and separation by preparative HPLC followed by characterisation by NMR spectroscopy (COSY, NOESY, Fig. 1) indicated the structure as the aldol product 3b. Single crystal X-ray diffraction analysis also confirmed the structure of 3b (Fig. 1A).

The apparent promiscuous ability of *Cv*TAm to catalyze an aldol addition of arylacetaldehydes 5a and 5b to 1 was intriguing and so initially studies were carried out to determine whether other transaminases could also catalyze the reaction. Using 5b and 1 again as starting materials, the (*R*)-selective transaminase from *Mycobacterium vanbaalenii* (*Mv*TAm)^27^ and *Arthrobacter* sp. (*Ar*Rmut11),^15^ and (*S*)-selective transaminases from *Vibrio fluvialis* (*Vf*TAm),^28^*Klebsiella pneumoniae* (*Kp*TAm; from UCL plasmid pQR1005)^29,30^ and *Rhodobacter sphaeroides* (*Rs*TAm; from UCL plasmid pQR 1019)^30,31^ were selected due to their previous biocatalytic applications. Several other transaminases from a drain metagenome, expressed from plasmids pQR2189, pQR2191, and pQR2208 which have shown good activity towards aromatic amines, were also selected to test in the reaction.^32,33^*Cv*TAm gave a reasonable yield (42%) of 3b. Notably, none of the other transaminases gave 3b (Table 1c). This data indicated that *Cv*TAm was distinctive in being able to effectively catalyse the aldol addition to give styryl pyridines.

**Fig. 2:**
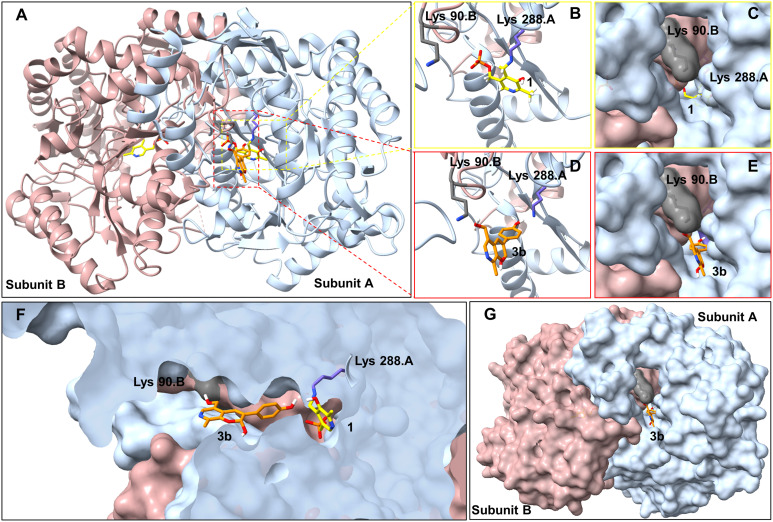
Molecular docking studies using *Cv*TAm (dimer)with styryl pyridine 3b. A. For aldolase activities, ligand 3b (orange) was located between Lys_288.A_ and Lys_90.B_ (subunit A in blue and subunit B in pink, active sites in red square). For transaminase activities, 1 interacts with Lys_288_. B and C. Transaminase active sites are located at the central of each subunit. D, E and G. Potential aldolase active sites located in a cleft between the dimeric subunits. F. Potential catalytic sites for the aldol condensation and (known) transamination are distinct. Figures were generated using UCSF ChimeraX.^43,44^

To try and establish the key residues in *Cv*TAm that gave rise to the aldolase activity, molecular docking studies with *apo Cv*TAm (PDB: 4BA4)^34^ and 3b were performed using AutoDock Vina.^35,36^ Analysis of the liganded structures revealed that 3b (for five conformations out of nine with the lowest energies) was located in a cleft between the dimeric subunits of *Cv*TAm (Fig. 2 and Table S2†). Thus, it is likely that the catalytic site for the aldol condensation is distinct from the catalytic site for transaminase activity (Fig. 2F). Type I aldolases such as 2-deoxyribose-5-phosphate aldolase (DERA), contain two key lysine residues in the catalytic site, one which forms a Schiff base intermediate, with a second residue nearby to perturb the p*K*_a_ of the reactive lysine.^37–39^ Inspired by this concept it was considered that key residues in *Cv*TAm for the aldol addition could be two lysines, positioned at the cleft between the subunits with one lysine residue located on each subunit and one lysine forming an imine with one of the substrates. Modelling positioned 3b in close proximity to Lys_288.A_ and Lys_90.B_ (the distance between Lys_288.A_ and Lys_90.B_ is 14.2 Å, and we recognise that this is a larger distance than in native and evolved aldolases,^37–39^ Fig. S7A†) suggesting that these may potentially be involved in the promiscuous aldolase activity. Other transaminases including *Vf*TAm (PDB:5ZTX,^40^ Lys_285.A_ and Lys_126.B_, Fig. S7B†) *Ar*Rmut11 (PDB: 3WWJ,^41^ Lys_188.A_ and Lys_142.B_, Fig. S7C†) have the equivalent residues. However, no aldol additions were observed, possibly due to the greater separation of the two lysine residues (24.4 Å for *Ar*Rmut11), although for *Vf*TAm the distance was similar to *Cv*TAm (14.6 Å), suggesting other factors may be important. No equivalent residues were found for *Kp*TAm (PDB: 3I5T,^42^ Fig. S7D†).

**Fig. 3:**
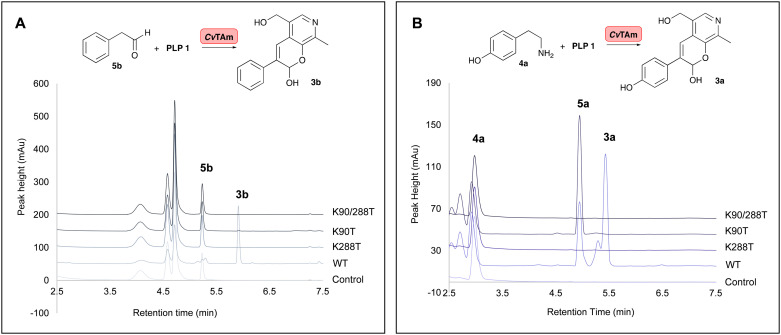
HPLC traces of *Cv*TAm reactions showing key HPLC product peaks. A. HPLC traces of *Cv*TAm reaction with 5b and 1; B. HPLC traces of the *Cv*TAm reaction with 4a and 1. Reaction conditions: 5b or 4a (10 mM, 1 equiv.) and 1 (15 mM, 1.5 equiv.) were added to reaction buffers, and 10% (v/v) DMSO as a co-solvent. To initiate the reactions, 0.1 mg mL^−1^ purified enzyme was added. Reactions were performed at 37 °C for 16 h and monitored by analytical HPLC at 280 nm.

To establish whether these are the key catalytic residues, the *Cv*TAm variants K288T, K90T and double mutant K90T/K288T were generated, as threonine has a smaller size compared to lysine, but retains the polar residue characteristics and is uncharged. The single mutants were generated by site directed mutagenesis and the K90T mutant was then used as a template for the creation of the double mutant. Reactions using phenylacetaldehyde 5b and 1 were performed with purified *Cv*TAm WT, K288T, K90T, K90T/K288T (0.1 mg mL^−1^) and an empty vector control. WT *Cv*TAm only gave the product 3b (Fig. 3A). When reacting tyramine 4a (to produce 5a*in situ*) and 1, the aldol product 3a again was only formed with WT *Cv*TAm (Fig. 3B). The experiments with K288T resulted in the loss of transamination activity with no formation of 5a as expected, as Lys_288_ is a key mechanistic lysine. In addition, while the transamination reactivity was retained in K90T giving 5a, the aldol addition activity was lost. The double mutant K90T/K288T also lost all transaminase and aldol activities (Fig. 3). These experiments supported the hypothesis that Lys_288_ and Lys_90_ have key mechanistic roles in the aldol reaction.

**Scheme 3:**
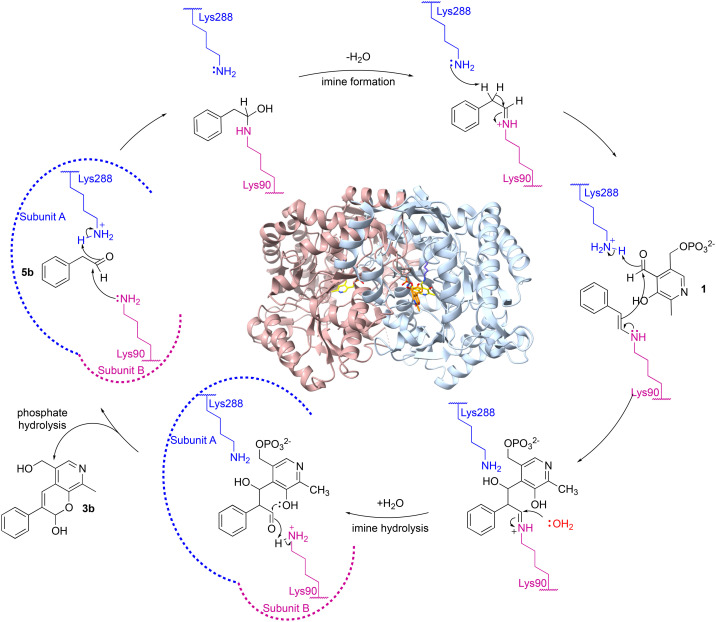
Proposed mechanism for the aldol addition by *Cv*TAm to give (after purification) styryl pyridine 3b. Note that the roles of Lys_288_ and Lys_90_ could be reversed.

A possible mechanism is shown in Scheme 3. Firstly, aldehyde 5b could be protonated by Lys_288.A_ and nucleophilic attack by Lys_90.B_, would give the carbinolamine. Protonation and subsequent loss of water would give the imine and subsequent deprotonation of the α-proton by Lys_288.A_, would give the key enamine. This can then attack the carbonyl carbon in 1 with protonation from Lys_288.A_ to give the aldol product and subsequent imine hydrolysis would give the corresponding aldehyde. Intramolecular hemiacetal formation followed by phosphate hydrolysis would generate 3b (Scheme 3). It was considered that phosphate hydrolysis likely occurred during the purification step and this is discussed further in the following section. Other potential aldol mechanisms are also possible such as switching which aldehyde forms the Lys imine: first PLP imine formation and then the aldol addition of 5b, with subsequent elimination of the Lys.

To investigate the potential competition between transamination activity and aldol activity, kinetic studies were performed. Firstly, the concentration of pyruvate was optimised. The results showed that pyruvate did not participate in the aldol addition but instead acted as an amine accepter for transamination activity. Therefore, pyruvate was used at 1 eq. to 4a (1 eq., 10 mM, Fig. S9†). Different ratios of 1 (0–2.5 eq.) to 4a (1 eq., 10 mM) were also tested with purified WT *Cv*TAm (0.1 mg mL^−1^). With lower equivalents of 1 (<0.1 eq.), only the transamination product 5a was formed. The aldol product 3a was observed at higher equivalents of PLP 1 (>0.2 eq.) and reached its maximum at 1.5 eq. of 1 (48% yield by HPLC analysis, Fig. S10†). Therefore, for the kinetic study of C*v*TAm for aldolase activity, 1 was used at 1.5 eq. to the substrate 4a and 1 eq. pyruvate was used. The *Cv*TAm kinetic studies were performed with 0.1 eq. of 1 and 1 eq. of pyruvate, revealing that the transaminase activity (*k*_cat.app_/*K*_m.app_ = 3.57 s^−1^ mM^−1^, *K*_m.app_ = 1.69 mM and *k*_cat.app_ = 6.04 s^−1^) is about 20 times higher than the aldolase activity (*k*_cat.app_/*K*_m.app_ = 0.18 s^−1^ mM^−1^, *K*_m.app_ = 9.84 mM and *k*_cat.app_ = 1.75 s^−1^). This also suggests that the aldolase activity has little effect on the transaminase reaction.

### Exploring the substrate scopes for the aldol addition to give styryl pyridines by *Cv*TAm

The substrate scope for the production of styryl pyridines using *Cv*TAm was then investigated. Tyramine 4a as before, dopamine 4c, *meta*-tyramine 4d and *ortho*-tyramine 4e were selected, with a view to them forming the corresponding aldehydes *in situ* and reacted with 1. Both 4a and 4e gave the products 3a and 3e, respectively (Table 2). While 4c and 4d can readily form the corresponding aldehydes,^23,24^ the presence of the *meta*-OH appeared to inhibit the aldol reaction. The scope of the aldehydes accepted was then investigated using 2-pyridinecarboxaldehyde 6a, 4-pyridinecarboxaldehyde 6b, pyrrole-2-carboxaldehyde 6c and pyrrole-2-carboxaldehyde 6d which were reacted with 4a and 5b. As shown in Table 2, the pyrrole carboxaldehydes were not accepted, while reactions using pyridine carboxaldehydes 6a and 6b gave the (*E*)-styryl pyridines 7a–7d in 35–46% (by analytical HPLC against product standards). It was noted that as previously reported,^33,45^ pyridine and pyrrole carboxaldehydes/ketones can be accepted by transaminases. Here, the pyridine-and pyrrole-substituted methylamine peaks had HPLC retention times that overlapped with 1 and sodium pyruvate, so competing aminations were not monitored. Benzaldehyde and analogues substituted with electron withdrawing groups, 2-, 3-, and 4-nitrobenzaldehyde, were also used with 5b, but no aldol products were observed. These results indicated that a pyridine structure appeared to be required in the electrophile for the aldol addition, perhaps to assist in the orientation of the substrate into a productive conformation.

**Table 2:** Exploring the substrate scopes of *Cv*TAm in aldol reactions to give styryl pyridines^a^

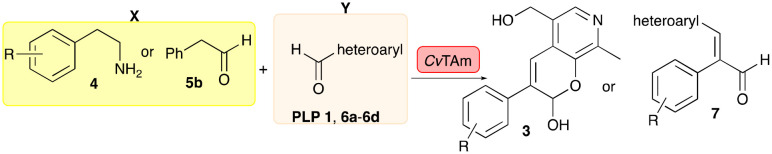			
***X***	***Y***	Product	Isolated yield (yield by HPLC)
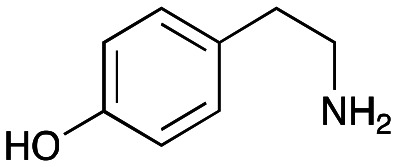 4a	1	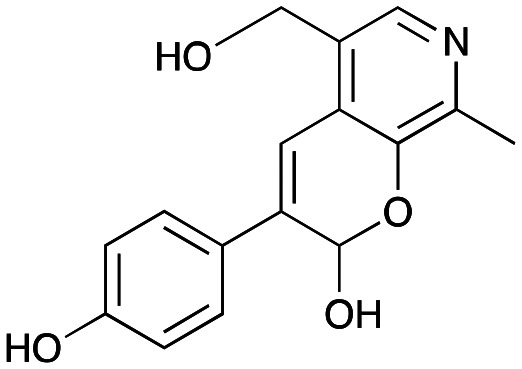 3a	**36%** (44%)
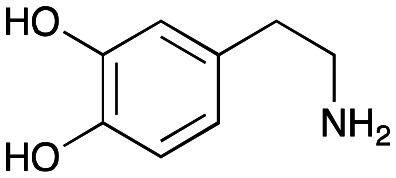 4c	1	No aldol product	—
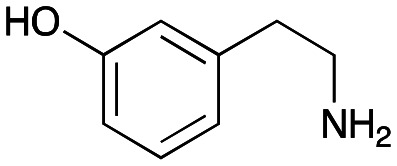 4d	1	No aldol product	—
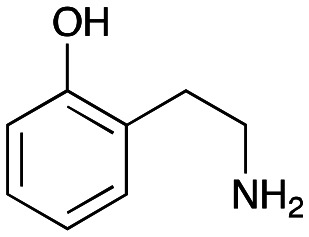 4e	1	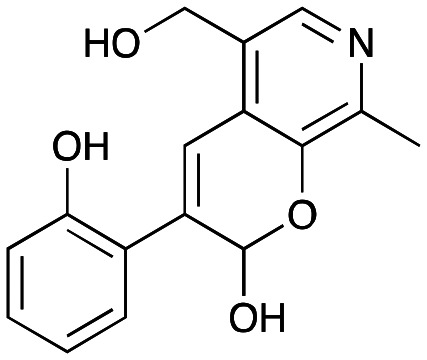 3e	**29%** (37%)
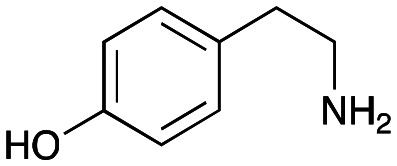 4a	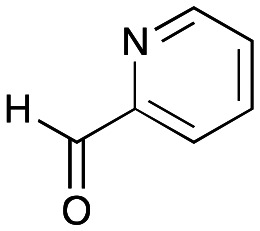 6a	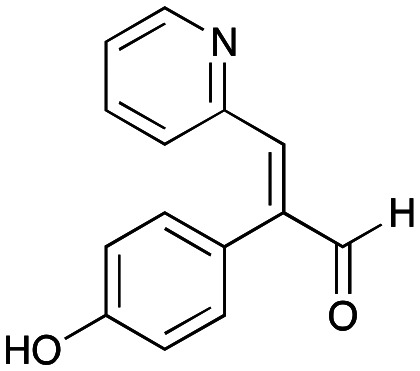 7a	**24%** (35%)
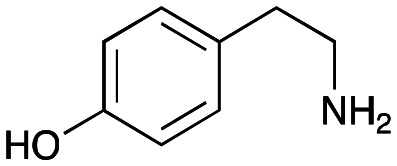 4a	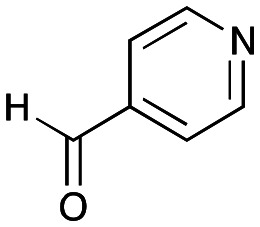 6b	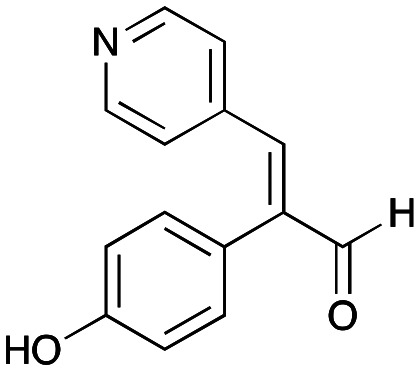 7b	**25%** (36%)
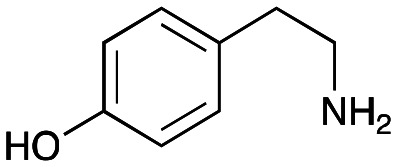 4a	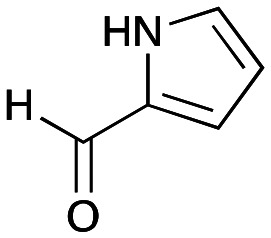 6c	No aldol product	—
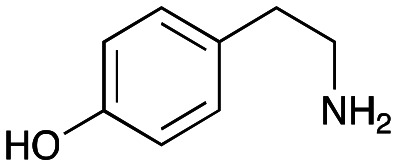 4a	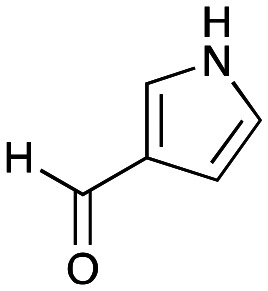 6d	No aldol product	—
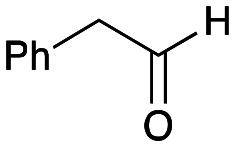 5b	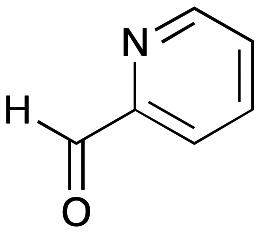 6a	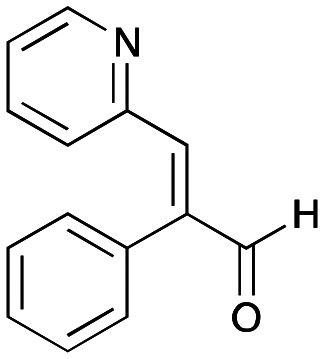 7c	**26%** (39%)
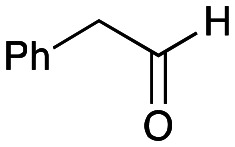 5b	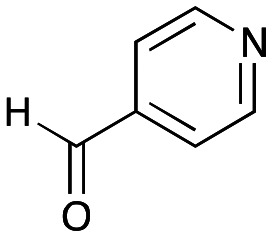 6b	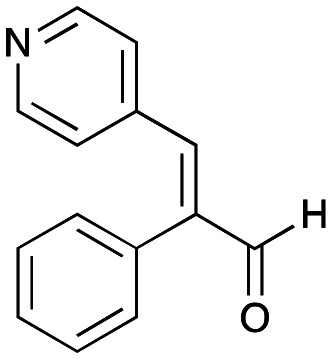 7d	**35%** (46%)

a *Reaction conditions*: starting materials ***X*** (10 mM, 1 equiv.) and ***Y*** (15 mM, 1.5 equiv.) were added to HEPES buffer (50 mM, pH 7.5), and 10% (v/v) DMSO was used as a co-solvent – 0.1 mmol reactions were performed. For reactions with 4a, 4c–4e, 1 mM 1 was also added for maintaining the transamination activity of *Cv*TAm and 10 mM pyruvate. To initial reactions, 10% (v/v) *Cv*TAm lysates (4 mg mL^−1^) were added. Reactions were then carried out at 37 °C for 16 h and monitored by analytical HPLC at 280 nm against product standards.

The phosphate hydrolysis step was then explored as the loss of phosphate was potentially due to the acidic work-up and purification method used. For the reaction between 1 and 4e (using the same conditions as previously), when this was quenched and purified in the absence of acid, it gave 3e-phosphate as the isolated product in 28% yield (ESI† section S3). This indicated that the phosphate hydrolysis was due to the work-up and purification conditions used, rather than a transaminase-mediated step.

### Enzyme cascades to halogenated hydroxystyryl pyridines

Enzyme cascades to halogenated hydroxystyryl pyridines were then developed, combining *Ef*TyrDC and *Cv*TAm and using 2a and the halogenated tyrosines 2b–2d as the starting materials as halogenated arylacetaldehydes are not readily available. First, the cascade to 3a was optimised from 2a using *Ef*TyrDC and *Cv*TAm to give the arylacetaldehyde, which directly condensed with 1 using *Cv*TAm giving 3a in 39% isolated yield. Similarly, 8a was formed from 2b in 37% yield (51% HPLC yield, Table 2). As 2b can also be hydroxylated by *Cn*TYR from previous work,^23,24^ this was also included in the cascade to the arylacetaldehyde, which with 1 gave the catechol styryl pyridine 8b in 35% yield. Finally, application of the cascade without wild-type *Cn*TYR, which does not readily accept 2c and 2d, gave products 8c and 8d in 36% and 29% isolated yields, respectively (Table 3). The ability to start from readily available amino acids, and through one pot two-enzyme three-step, or three-enzyme four-step cascades, to produce complex hydroxylated styryl pyridines is a valuable route to such compounds in 29–39% yields (41–51% yields by HPLC).

**Table 3:** One-pot enzyme cascades to halogenated hydroxystyryl pyridines

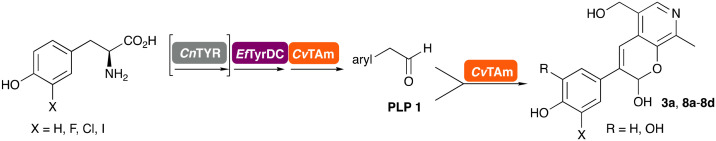			
Amino acid	Cascade route	Product	Isolated yield^a^ (yield by HPLC)^b^
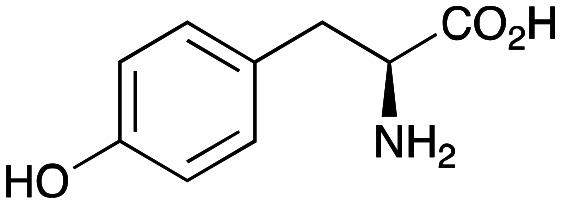 2a	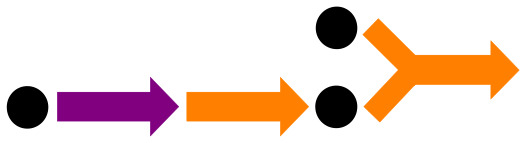	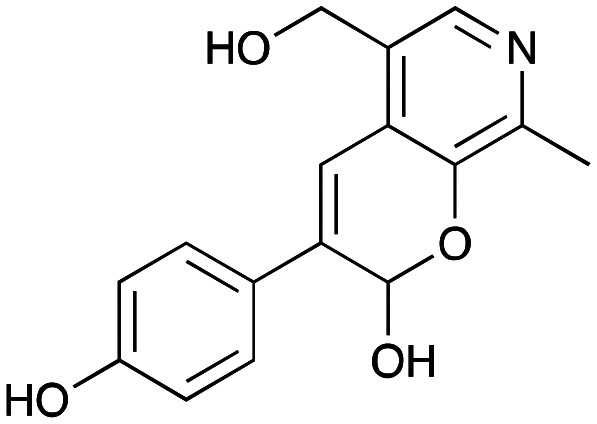 3a	**39%** (48%)
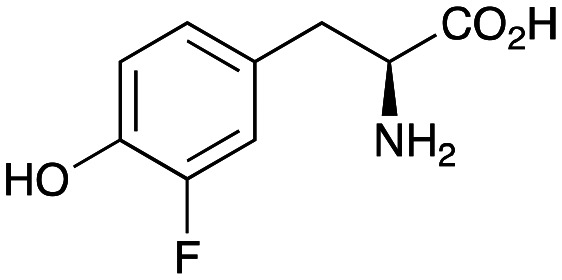 2b	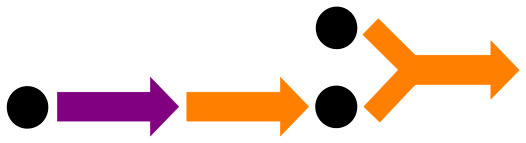	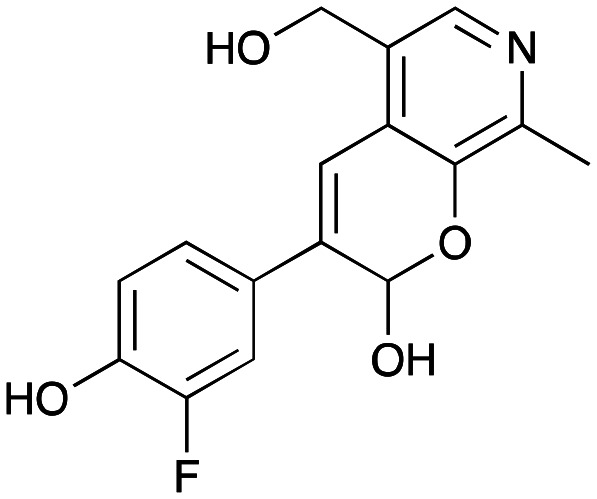 8a	**37%** (51%)
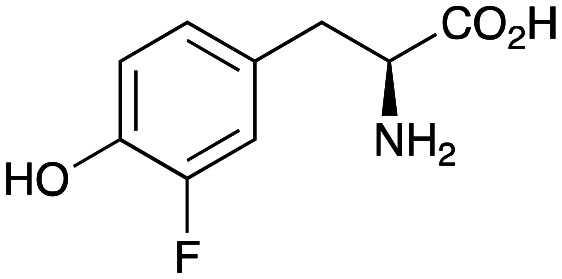 2b	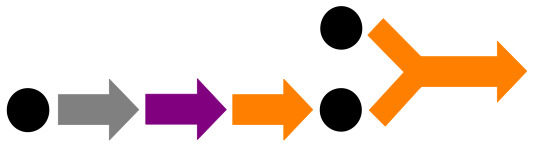	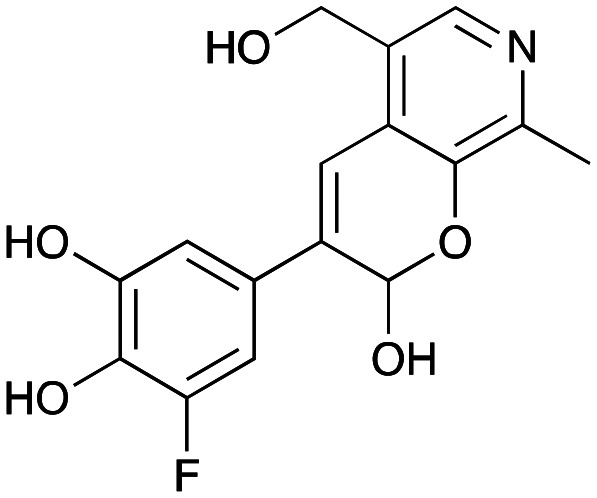 8b	**35%** (42%)
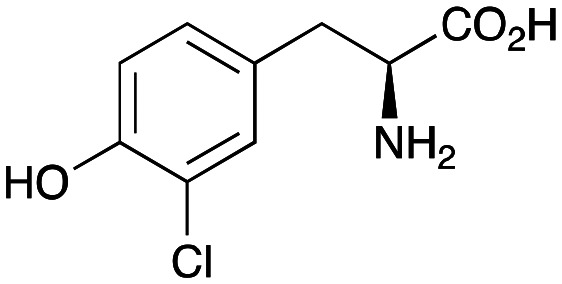 2c	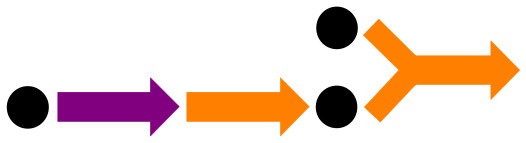	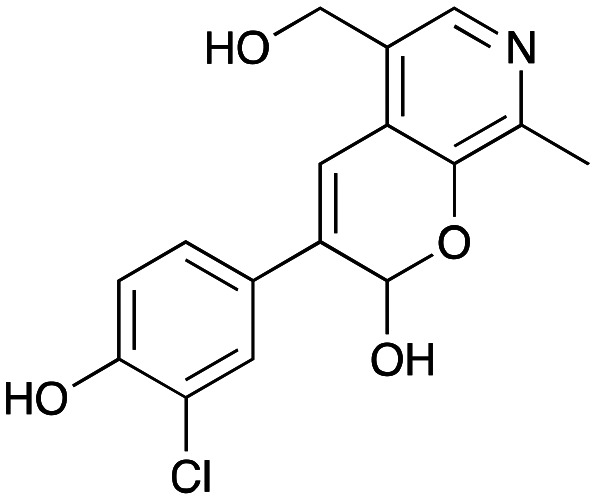 8c	**36%** (48%)
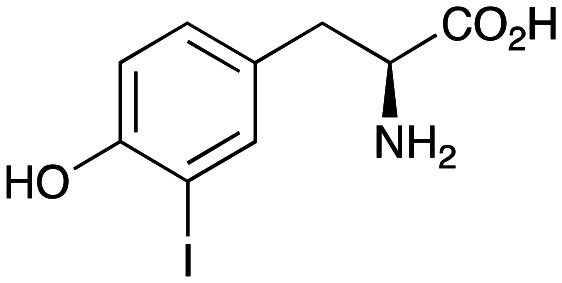 2d	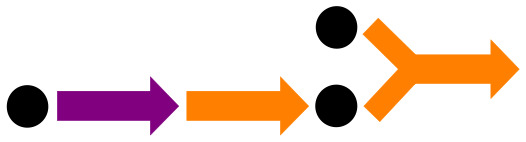	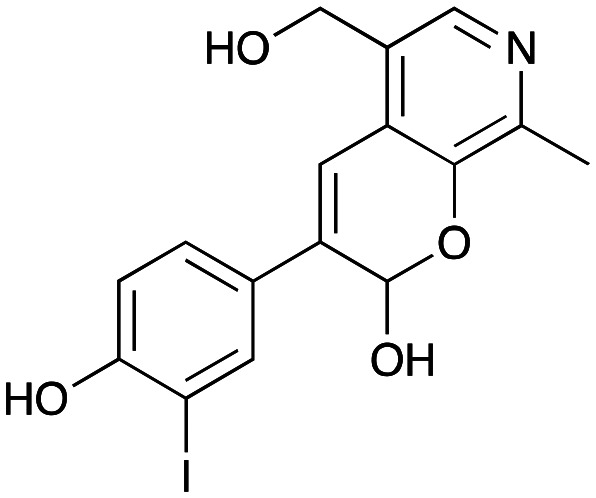 8d	**29%** (41%)

a *Reaction conditions*: 1 (10 mM, 1 equiv.) and substrate 2a–d (15 mM, 1.5 equiv.) were added to HEPES buffer (50 mM, pH 7.5) – 0.1 mmol reactions were performed. To initial reactions, 10% (v/v) enzyme lysates (4 mg mL^−1^ total enzyme) of *Cn*TYR (where used), *Ef*TyrDC and *Cv*TAm were added into one-pot. Reactions were performed at 37 °C for 16 h.

b Reactions were monitored by analytical HPLC at 280 nm against product standards.

As hydroxystyryl pyridines have an interesting pharmacophore, initial molecular dynamics modelling with 3a was investigated with human pancreatic amylase (HPA) (ESI† section S6), which suggested that they could be a potential inhibitor and useful scaffold for use in future studies.

## Conclusions

Here, a transaminase from *Chromobacterium violaceum* was identified as being able to catalyse the aldol addition between aryl acetaldehydes and pyridine carboxaldehydes, giving styryl pyridines and hydroxystyryl pyridines. This C–C bond formation is believed to be catalysed by two lysine residues Lys_288.A_ and Lys_90.B_ in the dimeric enzyme. Given the importance of hydroxystyryl pyridines as promising inhibitors for type II diabetes, enzyme cascades to various hydroxystyryl pyridines were established starting from commercially available amino acids. Above all, our data illustrates the potential of native enzymes to achieve promiscuous activities and produce novel products.

## Author contributions

Y. W. and Y. N. performed chemical syntheses, chemical characterizations, and enzymatic assays. D-K. B. carried out the X-ray crystallography experiments and J. W. E. J. generated the enzyme variants. P. A. D., Y. L. and Y. W. performed the molecular dynamic simulations. The manuscript was written through contributions of all authors. The project was conceived by Y. W., J. W. E. J. and H. C. H., and was supervised by J. M. W., J. W. E. J. and H. C. H. All authors have given approval to the final version of the manuscript.

## Conflicts of interest

There are no conflicts to declare.

## Supplementary Material

CY-014-D3CY01370G-s001

CY-014-D3CY01370G-s002

CY-014-D3CY01370G-s003

CY-014-D3CY01370G-s004

CY-014-D3CY01370G-s005
