# Supplementary material for: A transaminase-mediated aldol reaction and applications in cascades to styryl pyridines
Source: Catal Sci Technol. 2024 Mar 1;14(9):2390–9. doi: 10.1039/d3cy01370g (PMC11075132; doi:10.1039/d3cy01370g)

Electronic Supplementary Material (ESI) for Catalysis Science & Technology.
This journal is © The Royal Society of Chemistry 2024

Supporting Information

A Transaminase-Mediated Aldol Reaction and Applications in Cascades to Styryl Pyridines

Yu Wang,[a] Yiwen Li,[b] Yeke Ni,[a] Dejan-Krešimir Bučar,[a] Paul A. Dalby,[b] John M. Ward,[b] Jack W. E. Jeffries,[,b] and Helen C. Hailes[*,a]*

[a]Department of Chemistry, University College London, 20 Gordon Street, London, WC1H 0AJ,

[b]Department of Biochemical Engineering, University College London, Gower Street, London, WC1E 6BT, UK.

Contents

1. Enzyme preparation 4
1.1 Transaminase expression 4
1.2 Transaminase cell lysate preparation 4
1.3 Transaminase purification 4
1.4 Transaminase sequences used in this study 5
2 General analytic methods 7
2.1 Chemicals 7
2.2 Method for high performance liquid chromatography (HPLC) 7
2.2.1 Analytical HPLC Method 1 (achiral) 8
2.2.2 Preparative HPLC Method 2 8
2.3 Methods for mass spectrometry (MS) 9
2.4 Methods for nuclear magnetic resonance (NMR) spectroscopy 9
3 Synthesis and characterisation of styryl pyridines 10
3.1 5-(Hydroxymethyl)-3-(4-hydroxyphenyl)-8-methyl-2H-chromen-2-ol **3a** 10
3.2 5-(Hydroxymethyl)-8-methyl-3-phenyl-2H-pyrano[2,3-c]pyridin-2-ol **3b** 10
3.3 5-(Hydroxymethyl)-3-(2-hydroxyphenyl)-8-methyl-2H-pyrano[2,3-c]pyridin-2-ol **3e** 11
3.4 (E)-2-(4-Hydroxyphenyl)-3-(pyridin-2-yl)acrylaldehyde **7a** 12
3.5 (E)-2-(4-Hydroxyphenyl)-3-(pyridin-4-yl)acrylaldehyde **7b** 13
3.6 (E)-2-Phenyl-3-(pyridin-2-yl)acrylaldehyde **7c** 14
3.7 (E)-2-Phenyl-3-(pyridin-4-yl)acrylaldehyde **7d** 15
3.8 Hydroxystyryl pyridine cascade 1: 5-(Hydroxymethyl)-3-(4-hydroxyphenyl)-8-methyl-2H-pyrano[2,3-c]pyridin-2-ol **3a** 15
3.9 Hydroxystyryl pyridine cascade 2: 3-(3-Fluoro-4-hydroxyphenyl)-5-(hydroxymethyl)-8-methyl-2H-pyrano[2,3-c]pyridin-2-ol **8a** 16
3.10 Hydroxystyryl pyridine cascade 3: 3-Fluoro-5-(2-hydroxy-5-(hydroxymethyl)-8-methyl-2H-pyrano[2,3-c]pyridin-3-yl)benzene-1,2-diol **8b** 16
3.11 Hydroxystyryl pyridine cascade 4: 3-(3-Chloro-4-hydroxyphenyl)-5-(hydroxymethyl)-8-methyl-2H-pyrano[2,3-c]pyridin-2-ol **8c** 17

3.12 Hydroxystyryl pyridine cascade 5: 3-(3-Iodo-4-hydroxyphenyl)-5-(hydroxymethyl)-8-methyl-2H-pyrano[2,3-c]pyridin-2-ol **8d** ... 18

4 Single crystal X-ray diffraction studies ... 18

4.1 Crystal structure refinement process for **3a-OEt** ... 19

4.2 Crystal structure refinement process for **3b** ... 19

5. AutoDock of styryl pyridines with transaminases ... 22

6. Molecular dynamic simulations and MM-PBSA calculation ... 23

7. Kinetic studies with CvTAm ... 25

References ... 27

Appendix ... 28

Analytical HPLC traces for styryl pyridine products ... 28

NMR Spectroscopic data ... 31

Accurate Mass spectrum data ... 47

1. ***Enzyme preparation***

1.1 Transaminase expression

E. coli BL21 (DE3) was used as the expression host and plated out on agar plates supplemented with 50 μg/mL kanamycin. A single colony was then picked to inoculate into 5 mL of 2 x TY media supplemented with 50 μg/mL kanamycin and grown at 37 °C and 250 rpm overnight (8-16 h). 1 mL of the overnight cultures was then added into a 500 mL baffled shaking flask containing 100 mL of 2 x TY media supplemented with 50 μg/mL kanamycin at 37 °C, 250 rpm until an OD_{600} = 0.6. Enzyme expression was induced by the addition of 500 μM IPTG to the culture. Cultures were incubated overnight at 30 °C prior to harvesting, whilst shaking at 250 rpm. Cells were harvested by centrifugation (10,000 rpm, 15 mins) and the cell pellets was stored at −20 °C.

1.2 Transaminase cell lysate preparation

Cell pellets (50 mL culture) were resuspended in 5 mL of 50 mM HEPES buffer (pH 7.5) supplemented with 10 mM PLP and lysed by 10 cycles of sonication on ice (10 s on plus 10 s off, 12 watts output) equipped with a Process Timer. Cells lysates were centrifuged at 4 °C (10,000 rpm, 15 min). The supernatant was collected. The concentration of supernatant protein was measured following the standard Bradford procedure. The samples were duplicated and the average OD_{595} were used for cell lysate concentration calculations.

1.3 Transaminase purification

Cell lysates were filtered through a 0.2 μm cellulose acetate springe filter to remove insoluble cell components. A PD-10 column charged with Ni-NTA (5 mL) was washed with 10 mL of MilliQ^{TM} water, followed by 10 mL of binding buffer (50 mM HEPES, 10 mM imidazole (Sigma-Aldrich), pH 7.5). The filtered supernatant was then passed through the Ni-NTA column, and the column was washed with wash buffer (2 × 5 mL, 50 mM HEPES, 20 mM imidazole, pH 7.5) to remove some background protein. The bound protein was then eluted with elution buffer (50 mM HEPES, 500 mM imidazole, 100 mM NaCl, pH 7.5) until all the protein was collected. The eluent containing pure enzyme was concentrated using a vivaspin (30,000 MW) at 4 °C, (8,000 rpm, 5 min) until 2.5 mL eluent remained. Then the concentrated eluent was desalted into 3 mL of 50 mM HEPES (pH 7.5), using a Sephadex^{TM} G-25 in PD-10 column (GE Healthcare Life Sciences, Germany). To store the pure enzyme, 10% (v/v) glycerol was added. The concentration of the pure protein was measured by OD_{280} using a Nanodrop. The protein was split into different tubes with 0.5 mL/each, and stored at −20 °C. To check

the protein purity, the expression supernatant, flow through, wash, and eluents were examined using an SDS gel (Figure S1).

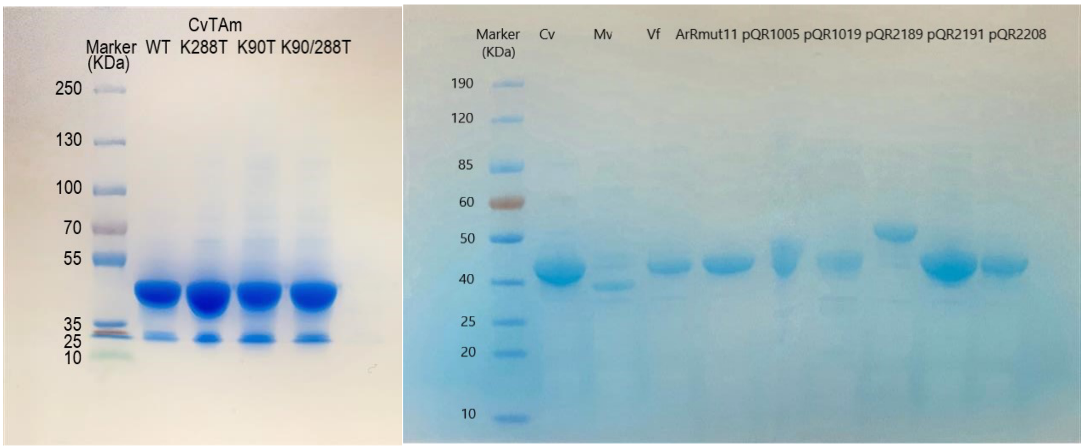

Figure S1. SDS-PAGE for transaminases. **A**. SDS-PAGE for purified *Cv*TAm and mutants (purified); **B**. SDS-PAGE for cell lysates of different transaminases; Protein Marker: Thermo Scientific™ PageRuler™ Plus Prestained Protein Ladder, 10 to 250 kDa; Invitrogen™ BenchMark™ Pre-stained Protein Ladder, 10 to 190 KDa.

1.4 Transaminase sequences used in this study

> Transaminase from *Chromobacterium violaceum* (*Cv*TAm, GenBank: AAQ59973.1)

MAKVYNFSAGPAVLPHQVLAEAQSELLDWHGSGMSVMEMSHRGKEFMEIIHDAEQDLRQLMGIPAGYKVLFLQGGASLQF
AMAPLNLLGDKDSIDIVNTGHWSKLAIKEAKRYAKVNVVASSEDRNFCYVPEEAAWQRDPNAAYLHYTSNETIGGLQFPY
IPAEQHGVPLVCDMSSDFLSREVDVSRFGMIYAGAQKNIGPSGLTVLLIREDLLGKARADIPTMLNYQVHADADSMYNTP
GTYPIYIAGLVFKWLKEQGGVKGIATRNEEKAGLLYHVIDSSGGFYSTHIEQPFRSKMNVVFKLRDEALDEIFLLEARKN
GLAQLKGHRAVGGMRASIYNAMPIEGVKSLVNFMQDFARQYGHHHHHH

> Transaminase from *Vibrio fluvialis* (*Vf*TAm, AEA39183.1)

MNKPQSWEARAETYSLYGFTDMPSLHQRGTVVVTHGEGPYIVDVNGRRYLDANSGLWNMVAGFDHKGLIDAAKAQYERFP
GYHAFFGRMSDQTVMLSEKLVEVSPFDSGRVFYTNSGSEANDTMVKMLWFLHAAEGKPQKRKILTRWNAYHGVTAVSASM
TGKPYNSVFGLPLPGFVHLTCPHYWRYGEEGETEEQFVARLARELEETIQREGADTIAGFFAEPVMGAGGVIPPAKGYFQ
AILPILRKYDIPVISDEVICGFGRTGNTWGCVTYDFTPDAIISSKNLTAGFFPMGAVILGPELSKRLETAIEAIEEFPHG
FTASGHPVGCAIALKAIDVVMNEGLAENVRRLAPRFEERLKHIAERPNIGEYRGIGFMWALEAVKDKASKTPFDGNLSVS
ERIANTCTDLGLICRPLGQSVVLCPPFILTEAQMDEMFDKLEKALDKVFAEVAHHHHHH

> Transaminase mutant from *Arthrobacter sp.* (*Ar*Rmut11, GenBank: BAK39753.1)

MAFSADTPEIVYTHDTGLDYITYSDYELDPANPLAGGAAWIEGAFVPPSEARISIFDQGFYTSDATYTTFHVWNGNAFRL
GDHIERLFSNAESIRLIPPLTQDEVKEIALELVAKTELREAMVTVTITRGYSSTPFERDITKHRPQVYMSACPYQWIVPF
DRIRDGVHLMVAQSVRRTPRSSIDPQVKNFQWGDLIRAIQETHDRGFELPLLLDCDNLLAEGPGFNVVVIKDGVVRSPGR
AALPGITRKTVLEIAESLGHEAILADITPAELYDADEVLGCSTGGGVWPFVSVDGNSISDGVPGPVTQSIIRRYWELNVE
PSSLLTPVQYALEHHHHHHHH

> Transaminase mutant from *Mycobacterium vanbaalenii* (*Mv*TAm, GenBank: WP_011781668.1)

MGIDTGTSNLVAVEPGAIREDTPAGSVIQYSDYEIDYSSPFAGGVAWIEGEYLPAEDAKISIFDTGFGHSDLTYTVAHVW
HGNIFRLGDHLDRLLDGARKLRLDSGYTKDELADITKKCVSLSQLRESFVNLTITRGYGKRKGEKDLSKLTHQVYIYAIP
YLWAFPPAEQIFGTTAVVPRHVRRAGRNTVDPTIKNYQWGDLTAASFEAKDRGARTAILMDADNCVAEGPGFNVCIVKDG

KLASPSRNALPGITRKTVFEIAGAMGIEAALRDVTSHELYDADEIMAVTTAGGVTPINTLDGVPIGDGEPGPVTVAIRDR
FWALMDEPGPLIEAIQYHHHHHH

> Transaminase mutant from *Klebsiella pneumoniae subsp. pneumoniae* (pQR1005, GenBank: ABR75708.1)
MNSNKAMMARRSDAVPRGVGQIHPIFAERAENCRVWDVEGREYLDFAGGIAVLNTGHLHPQVVAAVEDQLKKLSHTCFQV
LAYEPYLALCEKMNQKVPGDFAKKTLLVTTGSEAVENAVKIARAATGRSGAIAFTGAYHGRTHYTLSLTGKVNPYSAGMG
LMPGHVYRALYPCALHGVSDDEAIASIHRIFKNDAAPEDIAAIIIEPVQGEGGFYAASPAFMQRLRALCDEHGIMLIADE
VQSGAGRTGTLFAMEQMGVAADITTFAKSIAGGFPLAGVTGRAEVMDAIAPGGLGGTYAGNPIACAAALAVLQIFEQENL
LEKANQLGDTLRQGLLAIAEDHPEIGDVRGLGAMIAIELFEEGDHSRPNARLTADIVARARDKGLILLSCGPYYNVLRIL
VPLTIEEAQIEQGLKIIADCFSEAKQAHHHHHH

> Transaminase mutant from *Rhodobacter sphaeroides* (pQR1019, GenBank: ABP71721.1)
MALNDAAKAVGAVGAAMRDHVLLPAQEMAKLGKAAQPVLTHAEGIYVYVEDGRRLIDGPAGMWCAQVGYGRREIVDAMAH
QAMVLPYASPWYMASSPAARLAQKIATLTPGDLNRIFFTTGGSTAVDSALRFSEFYNNVLGRPQKKRIIVRYDGYHGSTA
LTAACTGRTGNWPNFDIAQDRISFLSSPNPRHAGNRSQEAFLDDLVQEFEDRIESLGPDTIAAFLAEPILASGGVIIPPK
GYHARFKAICEKHDILYISDEVVTGFGRCGEWFASEKVFGVVPDIITFAKGVTSGYVPLGGLAISEAVLARISGENARGS
WFTNGYTYSNQPVACAAALANIELMEREGLVDQAREMADYFAAALASLRDLPGVAETRSVGLVGCVQCLLDPTRADGTAE
DKAFTLKIDERCFELGLIVRPLGDLCVISPPLIISRAQIDDMVAIMRQAITEVGAAHGLTAKEPAAVHHHHHH

pDB:3I5T(96%identical to pQR1019)
MSLRNDATNAAGAVGAAMRDHILLPAQEMAKLGKSAQPVLTHAEGIYVHTEDGRRLIDGPAGMWCAQVGYGRREIVDAMA
HQAMVLPYASPWYMATSPAARLAEKIATLTPGDLNRIFFTTGGSTAVDSALRFSEFYNNVLGRPQKKRIIVRYDGYHGST
ALTAACTGRTGNWPNFDIAQDRISFLSSPNPRHAGNRSQEAFLDDLVQEFEDRIESLGPDTIAAFLAEPILASGGVIIPP
AGYHARFKAICEKHDILYISDEVVTGFGRCGEWFASEKVFGVVPDIITFAKGVTSGYVPLGGLAISEAVLARISGENAKG
SWFTNGYTYSNQPVACAAALANIELMEREGIVDQAREMADYFAAALASLRDLPGVAETRSVGLVGCVQCLLDPTRADGTA
EDKAFTLKIDERCFELGLIVRPLGDLCVISPPLIISRAQIDEMVAIMRQAITEVSAAHGLTAKEPAAVEGHHHHHH

Alignment of enzyme residues

```
pQR1019    -MALNDAAKAVGAVGAAMRDHVLLPAQEMAKLGKAAQPVLTHAEGIYVYVEDGRRLIDGPAGMWCAQVGYGRREIVDAMA  79
pDB: 3I5T  MSLRNDATNAAGAVGAAMRDHILLPAQEMAKLGKSAQPVLTHAEGIYVHTEDGRRLIDGPAGMWCAQVGYGRREIVDAMA  80
pQR1019    HQAMVLPYASPWYMASSPAARLAQKIATLTPGDLNRIFFTTGGSTAVDSALRFSEFYNNVLGRPQKKRIIVRYDGYHGST 159
pDB: 3I5T  HQAMVLPYASPWYMATSPAARLAEKIATLTPGDLNRIFFTTGGSTAVDSALRFSEFYNNVLGRPQKKRIIVRYDGYHGST 160
pQR1019    ALTAACTGRTGNWPNFDIAQDRISFLSSPNPRHAGNRSQEAFLDDLVQEFEDRIESLGPDTIAAFLAEPILASGGVIIPP 239
pDB: 3I5T  ALTAACTGRTGNWPNFDIAQDRISFLSSPNPRHAGNRSQEAFLDDLVQEFEDRIESLGPDTIAAFLAEPILASGGVIIPP 240
pQR1019    KGYHARFKAICEKHDILYISDEVVTGFGRCGEWFASEKVFGVVPDIITFAKGVTSGYVPLGGLAISEAVLARISGENARG 319
pDB: 3I5T  AGYHARFKAICEKHDILYISDEVVTGFGRCGEWFASEKVFGVVPDIITFAKGVTSGYVPLGGLAISEAVLARISGENAKG 320
pQR1019    SWFTNGYTYSNQPVACAAALANIELMEREGLVDQAREMADYFAAALASLRDLPGVAETRSVGLVGCVQCLLDPTRADGTA 399
pDB: 3I5T  SWFTNGYTYSNQPVACAAALANIELMEREGIVDQAREMADYFAAALASLRDLPGVAETRSVGLVGCVQCLLDPTRADGTA 400
pQR1019    EDKAFTLKIDERCFELGLIVRPLGDLCVISPPLIISRAQIDDMVAIMRQAITEVGAAHGLTAKEPAAVHHHHHH-- 473
pDB: 3I5T  EDKAFTLKIDERCFELGLIVRPLGDLCVISPPLIISRAQIDEMVAIMRQAITEVSAAHGLTAKEPAAVEGHHHHHH 476
```

* Residues that are identical among the sequences are given a black background, and those that are similar among the sequences are given a green background. The remaining residues receive a yellow background.

> pQR2189

MGSSHHHHHHSSGLVPRGSHMPRNHDIAELRRLDVAHHLPAQADWAEIEKLGGSRIITHAEGCYIHDGDGHRILDGMAGL
WCVNVGYGREELVEAAAAQMRELPFYNTFFKTATPPTVTLAAKIASLTGNRLPHIFFNASGSEANDTVFRMVRHYWKLKG
EPKRTVFISRWNAYHGSTVAGVSLGGMKAMHAQGDLPIPGIEHVRQPYSFGEGQGMTEEEFCDACVHAIEDKILEVGPEN
CAAFIGEPVQGAGGVVIPPKGYWPKVEAVARKYGLLVVSDEVICGFGRTGKMWGHETMGFTPDLMSMAKGLSSGYLPISA
TAVATHVVDVLKTGGDFVHGFTYSGHPVAAAVALKNIEIIEREGLVERTGSVTGPHLAKALATLNDHPLVGETRSIGLLG
AVEIVGEKVTRARFGGAEGTAGPMARDACIANGLMVRGIRDSLVMCPPLIISTEQIDEMVAIIRKSLDEVMPKLRALEHH
HHHH

> pQR2191

MGSSHHHHHHSSGLVPRGSHMSGQRDQELRARAAKVMPSSAFGHVGTALLPANYPQFFERAEGAYVWDADGNRYLDYMCA
FGPNLLGYRDPRVESAASAQAARGDVMTGPSPLAVELAEKFVEIVSHADWAFFCKNGTDATTIARTIARAQTGRRKILIA
EGSYHGAAPWCNPFPAGTVPEDRAHMLTFTFNDIASLEAAVAEAGDDLAGIIATPFKHEAFANQEFPTQDYARRCREICD
ASGAVLVVDDVRAGFRLAVDCSWATVGVKPDLSCWGKCFANGYSISAVMGSNRVKQGADSIFATGSFWQSAISMAAALAT
LDIIRDGKVIEKTVRLGQRLRDGLDEVSRRHGFTLNQTGPVQMPQILFEGDPDFRVGFAWTSAMIDRGFYLHPWHNMFLC
DAMTEEDIDQTIEAADSAFATVRAALPTLQPHERVLALFSARAEHHHHHH

> pQR2208

MTLRNYDMAELKRLDLAHHLPAQASYGLIRDLGGSRIITRAEGSTIWDAEGNAILDGMAGLWCVDVGYGRAELAEVAREQ
MLELPYYNTFFRTATPPPVKLAAKIAGLLGGSLQHIFFNSSGSESNDTVFRLVRTYWALKGQPERTIFISRRNAYHGSTV
AGVSLGGMAAMHAQGGLPIAGIEHVMQPYAFGEGFGEDPEAFAARAAQEIEDRILAVGPEKVAAFIGEPVQGAGGVIIPP
PGYWPRVDAICRKYGILLVSDEVICGFGRLGEWFGFQKYGYTPDIVSMAKGLSSGYLPISATGVSSEIVETLRASGDDFV
HGYTYSGHPVAAAVALRNLEIIKREGLVDRVRDDLAPYFAKALATLDDHPLVGEARSVGLLGAVEIVSEKGTNHRFGGKE
GTAGPVVRDHCIAGGLMVRAIRDSIVMCPPYVITHDEIDRMVAIIRSALDKAAVDLGGGAHHHHHH

2 General analytic methods

2.1 Chemicals

Compounds PLP **1**, L-tyrosine **2a**, tyramine **4a**, dopamine **4c**, *ortho*-tyramine **4e**, phenylacetaldehyde **5b**, 2-pyridinecarboxaldehyde **6a**, 4-pyridinecarboxaldehyde **6b**, pyrrole-2-carboxaldehyde **6c**, pyrrole-3-carboxaldehyde **6d**, 3-Cl-L-tyrosine **2c**, 3-I-L-tyrosine **2d**, kanamycin and sodium pyruvate were purchased from Sigma-Aldrich (Germany). Compounds *meta*-tyramine **4c**, 3-F-L-tyrosine **2b** and IPTG were purchased from Alfa Aesar (Thermo Fisher Scientific, USA). All chemicals were purchased in the highest purity available.

2.2 Method for high performance liquid chromatography (HPLC)

These were performed with a Dionex™ UltiMate™ 3000 HPLC System, with a Dionex™ UltiMate™ 3000 RS Pump, a Dionex™ UltiMate™ 3000 Autosampler, a Dionex™ UltiMate™ 3000 Column Compartment and a UltiMate™ 3000 RS Diode Array Detector (Thermofisher Scientific, US).

2.2.1 Analytical HPLC Method 1 (achiral)

Achiral quantitative analyses adopted a reverse phase analysis method. Separation was achieved with an ACE 5 C18 column (150 × 4.6 mm) with a flow speed of 1 mL/min at 30 °C. The injection volume was 10 µL. Substrates and products were measured via UV absorbance at 280 nm. Eluent A (H_2O with (v/v) 0.1% TFA) and eluent B (acetonitrile) were used as a mobile phase over 10 mins. The gradient is shown below (Figure S2).

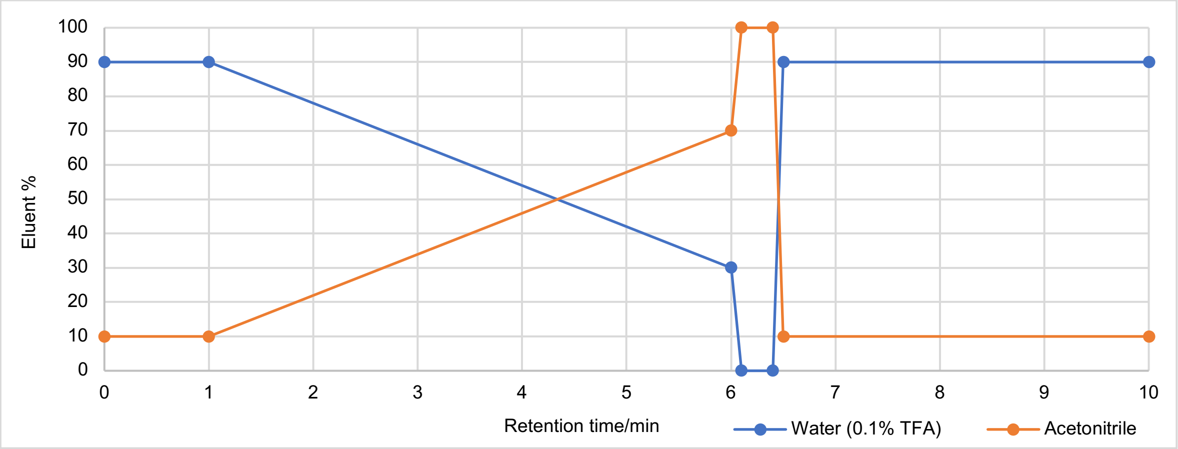

Figure S2. Gradient of achiral analytical HPLC method 1.

2.2.2 Preparative HPLC Method 2

Methods were developed with a Agilent 1260 Infinity^{TM} HPLC System, with a 1260 Infinity^{TM} Preparative Pump, a 1260 Infinity^{TM} Preparative-scale Fraction Collector, a 1260 Infinity^{TM} Multiple Wavelength Detector and a 1260 Infinity^{TM} Preparative Autosampler.

The separation was achieved with a Vydac^{TM} 218TP1022 (C18, 10 µm, 2.2 cm ID x 25 cm L) preparative column or a Supelco^{TM} Discovery BIO wide pore (C18, 10 µm, 2.12 cm x 25 cm) preparative column and a flow speed of 8 mL/min at 25 °C. The injection volume was 900 µL. Products were identified via UV absorbances at 214 nm and 280 nm. Eluent A (H_2O with 0.1% (v/v) TFA) and eluent B (acetonitrile with 0.1% (v/v) TFA) were used as a mobile phase over 28 mins. The gradient is shown below (Figure S3).

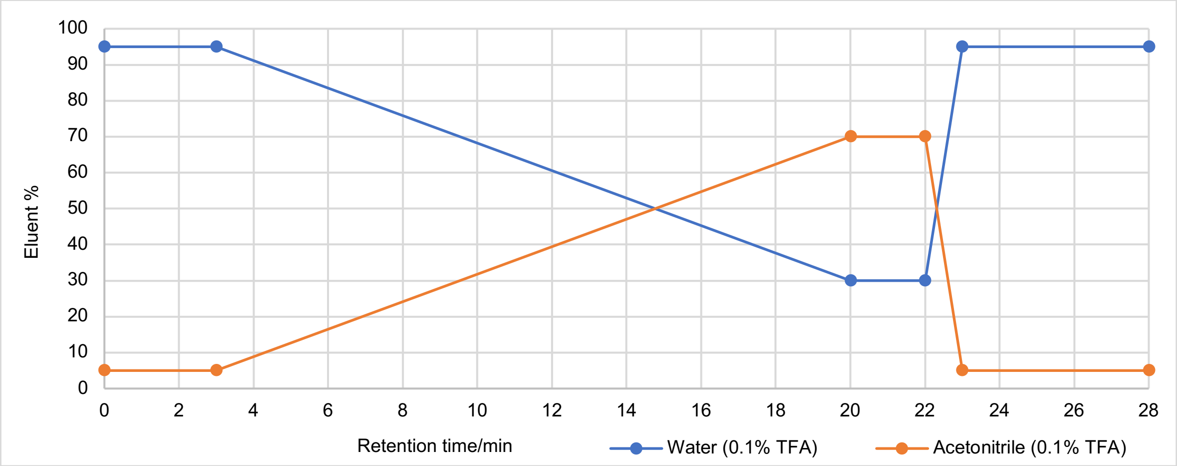

Figure S3. Gradient of preparative HPLC method 2.

2.3 Methods for *mass spectrometry (MS)*

The molecular masses of new compounds were measured on an Agilent 1100 Series System with a Finnigan LTQ mass spectrometer. An ACE 5 C18 reverse phase column (50 mm × 2.1 mm, 5 μm) was adopted with a mobile phase of eluent A (H_2O with 0.1% (v/v) formic acid) and eluent B (acetonitrile) over 5 min with a flow rate of 0.6 mL/min. The sample injection volume was 10 μL. Chemical compounds were measured in a positive ion mode, and the operating conditions of the ESI interface were set to a capillary temperature 300 °C, capillary voltage 9 V, spray voltage 4 kV, sheath gas 40, auxillary gas 10, sweep gas 0 arbitrary units. The gradient of eluents was as follows (Figure S4).

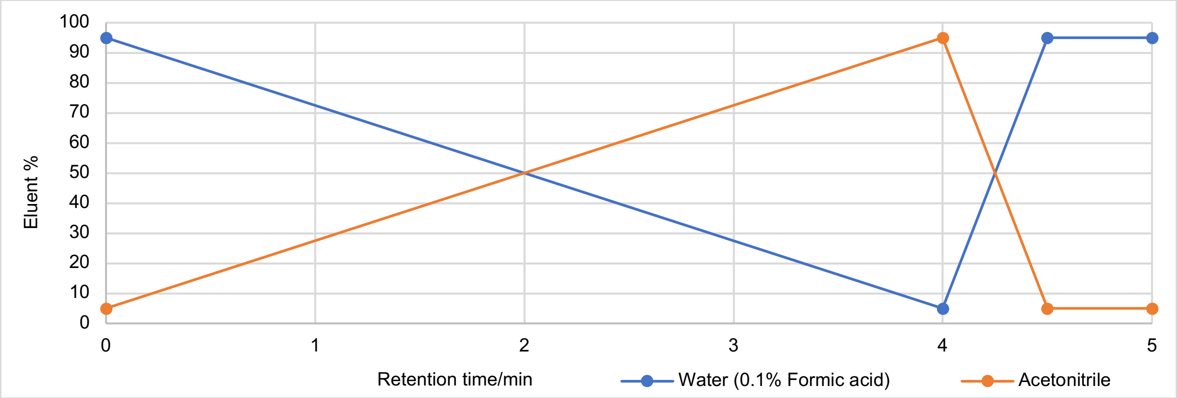

Figure S4. Gradient of the LC-MS method.

2.4 Methods for nuclear magnetic resonance *(NMR) spectroscopy*

^{1}H and ^{13}C NMR spectra were recorded respectively at 600 MHz and 150 MHz on a Bruker Avance 600 spectrometer or at 700 MHz and 175 MHz on a Bruker Avance 700 spectrometer in the stated solvent. Chemical shifts (in ppm) are quoted relative to tetramethylsilane and referenced to residual

protonated solvent. Coupling constants (*J*) are measured in Hertz (Hz) and multiplicities for ^{1}H NMR couplings are shown as s (singlet), d (doublet), t (triplet), and m (multiplet).

3 Synthesis and characterisation of styryl pyridines

*3.1 5-(Hydroxymethyl)-3-(4-hydroxyphenyl)-8-methyl-2H-chromen-2-ol **3a***

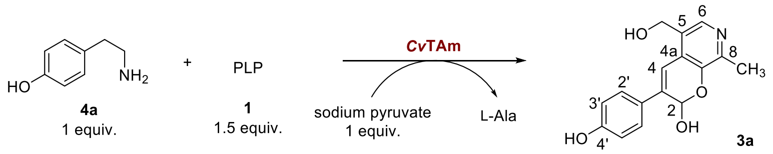

Tyramine **4a** (10 mM), PLP **1** (15 mM) and sodium pyruvate (10 mM) were dissolved in 10 mL HEPES buffer (50 mM, pH 7.5). To initiate the aldol addition, 10% (v/v) of *Cv*TAm lysate was added to the solution and the reaction was performed at 37 °C for 16 h. The reaction was quenched by adding 1% TFA. The product was purified using preparative HPLC (method 2, Supelco™ Discovery BIO wide pore (C18, 10 µm, 2.12 cm x 25 cm) preparative column, retention time: 14.8 min, run time: 28 mins, flow rate: 8 mL/min)). Fractions containing the desired product were freeze-dried to give product **3a** as an off-white powder (yield by HPLC against standards (calibration curve) 44% (method 1, ACE 5 C18 column (150 × 4.6 mm), retention time: 5.1 min, run time: 10 mins, flow rate: 1 mL/min); final isolated yield 10.3 mg, 36%. ^{1}H NMR (700 MHz; CD_3OD) δ 8.18 (1H, s, py-6-H), 7.67-7.65 (2H, m, 2 x 2’-H), 7.27 (1H, s, 4-H), 6.89-6.87 (2H, m, 2 x 3’-H), 6.57 (1H, s, 2-H), 4.97-4.92 (2H, m, C*H*$_2$OH), 2.68 (3H, s, py-8-CH_3); ^{13}C NMR (175 MHz; CD_3OD) δ 161.2, 146.6, 144.2, 142.9, 134.6, 132.5, 131.4, 129.5, 126.9, 116.9, 111.3, 92.5, 59.3, 14.3; *m/z* [ES+] 286 ($[M+H]^+$, 100%), *m/z* [HRMS ES+] found $[M+H]^+$ 286.1072; $[C_{16}H_{15}NO_4+H]^+$ requires 286.1074.

*3.2 5-(Hydroxymethyl)-8-methyl-3-phenyl-2H-pyrano[2,3-c]pyridin-2-ol **3b***

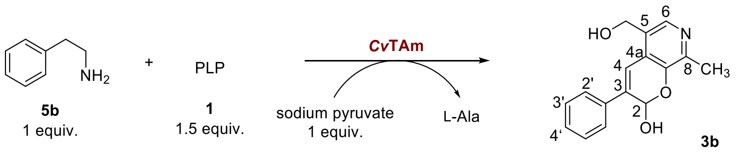

Phenylacetaldehyde **5b** (10 mM) and PLP **1** (15 mM) were dissolved in 10 mL HEPES buffer (50 mM, pH 7.5). To initiate the aldol addition, 10% (v/v) of *Cv*TAm lysate was added to the solution and the reaction was performed at 37 °C for 16 h. The reaction was quenched by adding 1% TFA. The product was purified using preparative HPLC (method 2, Supelco™ Discovery BIO wide pore (C18, 10 µm, 2.12 cm x 25 cm) preparative column, retention time: 16.4 min, run time: 28 mins, flow rate: 8 mL/min)). Fractions containing the desired product were freeze-dried to give product **3b** as an off-

white powder (yield by HPLC against standards (calibration curve) 42% (method 1, ACE 5 C18 column (150 × 4.6 mm), retention time: 6.0 min, run time: 10 mins, flow rate: 1 mL/min); final isolated yield 10.2 mg, 38%. ^{1}H NMR (600 MHz; CD_3OD) δ 8.24 (1H, s, py-6-H), 7.80-7.78 (2H, m, 2 x 2'-H), 7.51-7.46 (3H, m, 2 x 3'-H and 4'-H), 7.43 (1H, s, 4-H), 6.57 (1H, s, 2-H), 4.99-4.89 (2H, m, CH_2OH), 2.70 (3H, s, py-8-CH_3); ^{13}C NMR (151 MHz; CD_3OD) δ 147.3, 144.3, 143.5, 136.1, 135.4, 134.3, 132.4, 131.4, 130.2, 127.8, 114.5, 92.7, 59.3, 14.4; *m/z* [ES+] 270 ([M+H]$^+$, 100%), *m/z* [HRMS ES+] found [M+H]$^+$ 270.1124; [$C_{16}H_{15}NO_4$+H]$^+$ requires 270.1125.

*3.3 5-(Hydroxymethyl)-3-(2-hydroxyphenyl)-8-methyl-2H-pyrano[2,3-c]pyridin-2-ol **3e***

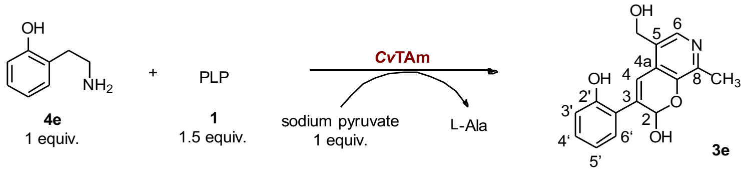

Ortho-Tyramine **4e** (10 mM), PLP **1** (15 mM) and sodium pyruvate (10 mM) were dissolved in 10 mL HEPES buffer (50 mM, pH 7.5). To initiate the aldol addition, 10% (v/v) of *Cv*TAm lysate was added to the solution and the reaction was carried out at 37 °C for 16 h. The reaction was quenched by adding 1% TFA. The product was purified using preparative HPLC (method 2, Supelco^{TM} Discovery BIO wide pore (C18, 10 μm, 2.12 cm x 25 cm) preparative column, retention time: 14.6 min, run time: 28 mins, flow rate: 8 mL/min)). Fractions containing the desired product were freeze-dried to give product **3e** as an off-white powder (yield by HPLC against standards (calibration curve) 37% (method 1, ACE 5 C18 column (150 × 4.6 mm), retention time: 5.1 min, run time: 10 mins, flow rate: 1 mL/min); final isolated yield 8.3 mg, 29%. ^{1}H NMR (600 MHz; CD_3OD) δ 8.26 (1H, s, py-6-H), 7.53 (1H, dd, *J* = 7.7 Hz & 1.7 Hz, 6'-H), 7.46-7.44 (1H, m, 4'-H), 7.27-7.25 (1H, m, 5'-H), 6.93-6.89 (2H, m, 3'-H and 4-H), 6.71 (1H, s, 2-H), 5.19-5.15 (2H, m, CH_2OH), 2.68 (3H, s, py-8-CH_3); ^{13}C NMR (151 MHz; CD_3OD) δ 157.4, 147.5, 145.5, 143.9 135.7, 132.9, 132.5, 131.2, 123.3, 121.2, 117.3, 116.9, 105.0, 92.9, 62.9, 14.3; *m/z* [ES+] 286 ([M+H]$^+$, 100%), *m/z* [HRMS ES+] found [M+H]$^+$ 286.1072; [$C_{16}H_{15}NO_4$+H]$^+$ requires 286.1074.

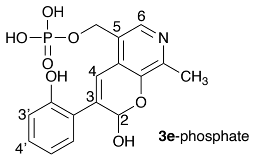

Note: 3e-phosphate was observed if an alternative purification system was used in the absence of

acidic conditions: *Ortho*-Tyramine **4e** (10 mM), PLP **1** (15 mM) and sodium pyruvate (10 mM) were dissolved in 10 mL HEPES buffer (50 mM, pH 7.5). To initiate the aldol addition, 10% (v/v) of *Cv*TAm lysate was added to the solution and the reaction was carried out at 37 °C for 16 h. The reaction was quenched by freeze drying. The product was purified using preparative HPLC (method 2, solvents without acid addition, Supelco™ Discovery BIO wide pore (C18, 10 µm, 2.12 cm x 25 cm) preparative column, retention time: 14.4 min, run time: 28 mins, flow rate: 8 mL/min)). Fractions containing the desired product were freeze-dried to give product **3e**-phosphate as an off-white powder (yield by HPLC against standards (calibration curve) 35% (method 1, ACE 5 C18 column (150 × 4.6 mm), retention time: 5.1 min, run time: 10 mins, flow rate: 1 mL/min); final isolated yield 10.2 mg, 28%. ^{1}H NMR (600 MHz; CD_3OD) δ 8.30 (1H, s, py-6-H), 7.53 (1H, d, *J* = 7.7 Hz, 6’-H), 7.49-7.45 (1H, m, 4’-H), 7.27 (1H, t, *J* = 7.7 Hz, 5’-H), 6.95-6.87 (2H, m, 3’-H and 4-H), 6.74 (1H, s, 2-H), 5.82-5.78 (2H, m, $CH_2OPO_3H_2$), 2.68 (3H, s, py-8-CH_3); ^{13}C NMR (151 MHz; CD_3OD) δ 157.4, 147.2, 145.0, 143.7, 135.4, 132.4, 131.2, 130.7, 124.9, 121.1, 117.3, 117.0, 107.1, 92.9, 65.7 ($J_{p\text{-}c}$ = 7.1 Hz), 17.2; *m/z* [ES+] 366 ($[M+H]^+$, 100%), *m/z* [HRMS ES+] found $[M+H]^+$ 366.0733; $[C_{16}H_{16}NO_7P+H]^+$ requires 366.0737.

*3.4 (E)-2-(4-Hydroxyphenyl)-3-(pyridin-2-yl)acrylaldehyde **7a***

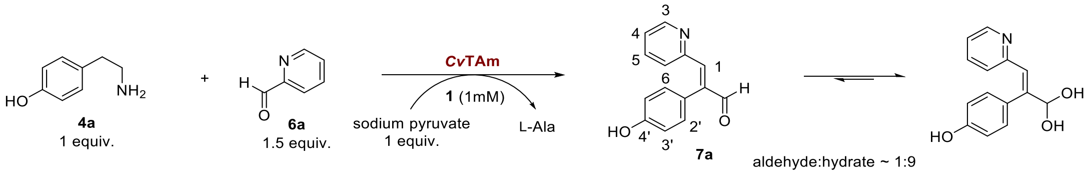

Tyramine **4a** (10 mM), 2-pyridinecarboxaldehyde **6a** (10 mM), PLP **1** (1 mM) and sodium pyruvate (10 mM) were dissolved in 10 mL HEPES buffer (50 mM, pH 7.5). To initiate the aldol addition, 10% (v/v) of *Cv*TAm lysate was added to the solution and the reaction was carried out at 37 °C for 16 h. The reaction was quenched by adding 1% TFA. The product was purified using preparative HPLC (method 2, Supelco™ Discovery BIO wide pore (C18, 10 µm, 2.12 cm x 25 cm) preparative column, retention time: 15.3 min, run time: 28 mins, flow rate: 8 mL/min)). Fractions containing the desired product were freeze-dried to give product **7a** as a dark red powder (yield by HPLC (calibration curve) 35% (method 1, ACE 5 C18 column (150 × 4.6 mm), retention time: 5.3 min, run time: 10 mins, flow rate: 1 mL/min); final isolated yield 5.5 mg, 24%. The aldehyde also formed the hydrate in CD_3OD, ratio aldehyde:hydrate, ~1:9.

Aldehyde: ^{1}H NMR (600 MHz; CD_3OD) δ 9.82 (1H, s, CHO) 8.63-8.61 (1H, m, py-3-H), 7.71-7.70 (1H, m, py-6-H), 7.48 (1H, s, 1-H), 7.39-7.37 (1H, m, py-4-H), 7.17-7.16 (1H, m, py-5-H), 7.11-7.08 (2H, m, 2 x 2'-H), 6.82-6.78 (2H, m, 2 x 3'-H); ^{13}C NMR (151 MHz; CD_3OD) δ 195.8 (CHO), 159.5, 154.5, 149.8, 147.1, 146.6, 142.8, 139.0, 132.0, 126.9, 125.5, 116.7;

Hydrate: ^{1}H NMR (600 MHz; CD_3OD) δ 8.63-8.61 (1H, m, py-3-H), 8.11 (1H, m, py-5-H), 7.68 (1H, ddd, *J* = 8.4 Hz, 6.0 Hz, 1.2 Hz, py-4-H), 7.27 (1H, d, *J* = 8.4 Hz, py-6-H), 7.11-7.08 (2H, m, 2 x 2'-H), 6.99 (1H, s, 1-H), 6.81-6.78 (2H, m, 2 x 3'-H), 5.18 (1H, d, *J* = 1.2 Hz, $CH(OH)_2$, coupling to 1-H); ^{13}C NMR (151 MHz; CD_3OD) δ 159.9, 152.9, 151.9, 145.1, 143.4, 131.3, 128.1, 127.3, 125.6, 121.2, 117.0, 105.2 ($CH(OH)_2$); *m/z* [ES+] 226 ($[M+H]^+$, 100%), *m/z* [HRMS ES+] found $[M+H]^+$ 226.0857; $[C_{14}H_{11}NO_2+H]^+$ requires 226.0863.

*3.5 (E)-2-(4-Hydroxyphenyl)-3-(pyridin-4-yl)acrylaldehyde **7b***

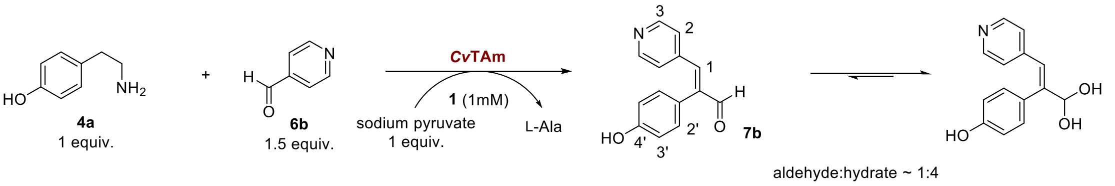

Tyramine **4a** (10 mM), 4-pyridinecarboxaldehyde **6b** (10 mM), PLP **1** (1 mM) and sodium pyruvate (10 mM) were dissolved in 10 mL HEPES buffer (50 mM, pH 7.5). To initiate the aldol addition, 10% (v/v) of *Cv*TAm lysate was added to the solution and the reaction was performed at 37 °C for 16 h. The reaction was quenched by adding 1% TFA. The product was purified using preparative HPLC (method 2, Supelco™ Discovery BIO wide pore (C18, 10 μm, 2.12 cm x 25 cm) preparative column, retention time: 15.1 min, run time: 28 mins, flow rate: 8 mL/min)). Fractions containing the desired product were freeze-dried to give product **7b** as an off-white powder (yield by HPLC (calibration curve) 36% (method 1, ACE 5 C18 column (150 × 4.6 mm), retention time: 5.2 min, run time: 10 mins, flow rate: 1 mL/min); final isolated yield 5.8 mg, 25%. The aldehyde also formed the hydrate in CD_3OD, ratio aldehyde:hydrate, ~1:4. Aldehyde: ^{1}H NMR (600 MHz; CD_3OD) δ 9.85 (1H, s, CHO), 8.61 (2H, d, *J* = 6.6 Hz, 2 x py-3-H), , 7.64 (2H, d, *J* = 6.6 Hz, 2 x py-2-H), 7.56 (1H, s, 1-H), 7.02-7.01 (2H, m, 2 x 2'-H), 6.83-6.82 (2H, m, 2 x 3'-H); ^{13}C NMR (151 MHz; CD_3OD) δ 195.0 (CHO), 160.1, 157.4, 154.7, 149.0, 145.1, 143.0, 131.4, 127.5, 117.0;

Hydrate: δ 8.52 (2H, d, *J* = 6.6 Hz, 2 x py-3-H), 7.57 (2H, d, *J* = 6.6 Hz, 2 x py-2-H), 7.09-7.07 (2H, m, 2 x 2'-H), 7.00 (1H, s, 1-H), 6.81-6.78 (2H, m, 2 x 3'-H), 5.14 (1H, d, *J* = 1.2 Hz, $CH(OH)_2$, coupling

to 1-H); ^{13}C NMR (151 MHz; CD_3OD) δ 152.0, 156.9, 151.6, 142.2, 132.2, 131.4, 128.0, 125.2, 117.1, 105.8 ($CH(OH)_2$); *m/z* [ES+] 226 ($[M+H]^+$, 100%), *m/z* [HRMS ES+] found $[M+H]^+$ 226.0866; $[C_{14}H_{11}NO_2+H]^+$ requires 226.0863.

3.6 (E)-2-Phenyl-3-(pyridin-2-yl)acrylaldehyde ***7c***

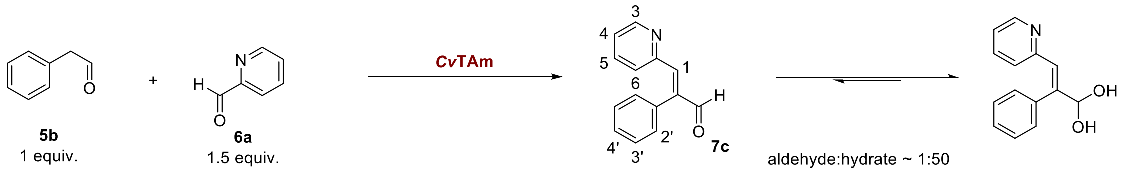

Phenylacetaldehyde **5b** (10 mM) and 2-pyridinecarboxaldehyde **6a** (15 mM) were dissolved in 10 mL HEPES buffer (50 mM, pH 7.5). To initiate the aldol addition, 10% (v/v) of *Cv*TAm cell lysate was added to the solution and the reaction was performed at 37 °C for 16 h. The reaction was quenched by adding 1% TFA. The product was purified using preparative HPLC (method 2, Supelco™ Discovery BIO wide pore (C18, 10 μm, 2.12 cm × 25 cm) preparative column, retention time: 18.1 min, run time: 28 mins, flow rate: 8 mL/min)). Fractions containing the desired product were freeze-dried to give product **7c** as a dark red powder (yield by HPLC (calibration curve) 39% (method 1, ACE 5 C18 column (150 × 4.6 mm), retention time: 6.3 min, run time: 10 mins, flow rate: 1 mL/min); final isolated yield 5.5 mg, 26%. The aldehyde also formed the hydrate in CD_3OD, ratio aldehyde:hydrate, ~1:50.

Aldehyde: ^{1}H NMR (600 MHz; CD_3OD) δ 9.85 (1H, s, CHO), 8.67-8.66 (1H, m, py-3-H), 7.60 (1H, s, 1-H), 7.32-7.30 (6H, m, py-4-H, py-5-H, py-6-H, 2 x 3'-H and 4'-H), 7.31-7.30 (2H, m, 2 x 2'-H); ^{13}C NMR (151 MHz; CD_3OD) δ 195.3 (CHO), 158.5, 156.9, 146.9, 143.0, 139.9, 138.8, 130.2, 129.6, 128.5, 126.2, 124.8;

Hydrate: ^{1}H NMR (600 MHz; CD_3OD) δ 8.67-8.66 (1H, m, py-3-H), 8.09 (1H, td, *J* = 8.4 Hz, 1.8 Hz, py-5-H), 7.71 (1H, ddd, *J* = 8.4 Hz, 6.0 Hz, 1.2 Hz, py-4-H), 7.42-7.39 (3H, m, 2 x 3'-H and 4'-H), 7.28-7.26 (2H, m, 2 x 2'-H), 7.17 (1H, d, *J* = 8.4 Hz, py-6-H), 7.10 (1H, s, 1-H), 5.21 (1H, s, C*H*$(OH)_2$); ^{13}C NMR (151 MHz; CD_3OD) δ = 152.0, 145.4, 143.4, 136.7, 130.3, 130.2, 129.8, 129.5, 128.0, 126.0, 122.0, 105.2 ($CH(OH)_2$); *m/z* [ES+] 210 ($[M+H]^+$, 100%), *m/z* [HRMS ES+] found $[M+H]^+$ 210.0917; $[C_{14}H_{11}NO_2+H]^+$ requires 210.0913.

*3.7 (E)-2-Phenyl-3-(pyridin-4-yl)acrylaldehyde **7d***

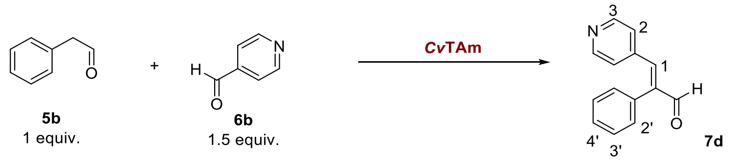

Phenylacetaldehyde **5b** (10 mM) and 4-pyridinecarboxaldehyde **6b** (15 mM) were dissolved in 10 mL HEPES buffer (50 mM, pH 7.5). To initiate the aldol addition, 10% (v/v) of *Cv*TAm cell lysate was added to the solution and the reaction was performed at 37 °C for 16 h. The reaction was quenched by adding 1% TFA. The product was purified using preparative HPLC (method 2, Supelco™ Discovery BIO wide pore (C18, 10 µm, 2.12 cm x 25 cm) preparative column, retention time: 16.1 min, run time: 28 mins, flow rate: 8 mL/min)). Fractions containing the desired product were freeze-dried to give product **7d** as an off-white powder (yield by HPLC (calibration curve) 46% (method 1, ACE 5 C18 column (150 × 4.6 mm), retention time: 5.7 min, run time: 10 mins, flow rate: 1 mL/min); final isolated yield 7.3 mg, 35%.^{1}H NMR (600 MHz; CD_3OD) δ 9.83 (1H, s, CHO), 8.41 (2H, d, *J* = 6.0 Hz, 2 x py-3-H), 7.61 (1H, s, 1-H), 7.42-7.41 (3H, m, 2 x 3'-H and 4'-H), 7.18 (2H, d, *J* = 6.0 Hz, 2 x py-2-H), 7.15-7.14 (2H, m, 2 x 2'-H); ^{13}C NMR (151 MHz; CD_3OD) δ 195.4, 150.3, 147.4, 147.1, 144.2, 133.7, 130.4, 129.9, 129.8, 125.7; *m/z* [ES+] 210 ($[M+H]^+$, 100%), *m/z* [HRMS ES+] found $[M+H]^+$ 210.0913; $[C_{14}H_{11}NO_2+H]^+$ requires 210.0913.

*3.8 Hydroxystyryl pyridine cascade 1: 5-(Hydroxymethyl)-3-(4-hydroxyphenyl)-8-methyl-2H-pyrano[2,3-c]pyridin-2-ol **3a***

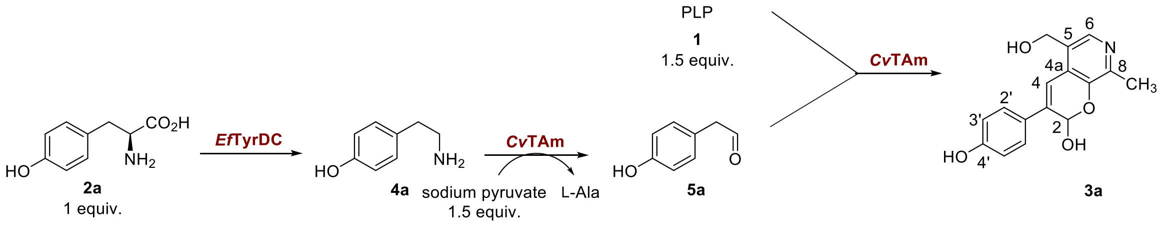

The reaction mixture (40 mL, pH 7.5) consisted of 50 mM HEPES, 2.5 mM L-tyrosine **2a**, 4 mM PLP **1** and 2.5 mM sodium pyruvate. To initiate the reaction, 10% (v/v) of *Ef*TyrDC lysate and 10% (v/v) of *Cv*TAm lysate was added to the solution which was incubated at 37 °C, 250 rpm for 16 h. The reaction was quenched by adding 1% TFA. The product was purified using preparative HPLC (method 2, Supelco™ Discovery BIO wide pore (C18, 10 µm, 2.12 cm x 25 cm) preparative column, retention time: 14.8 min, run time: 28 mins, flow rate: 8 mL/min)). Fractions containing the desired product were freeze-dried to give product **3a** as a yellow powder (yield by HPLC (calibration curve)

48% (method 1, ACE 5 C18 column (150 × 4.6 mm), retention time: 5.1 min, run time: 10 mins, flow rate: 1 mL/min); final isolated yield 11.1 mg, 39%.

*3.9 Hydroxystyryl pyridine cascade 2: 3-(3-Fluoro-4-hydroxyphenyl)-5-(hydroxymethyl)-8-methyl-2H-pyrano[2,3-c]pyridin-2-ol **8a***

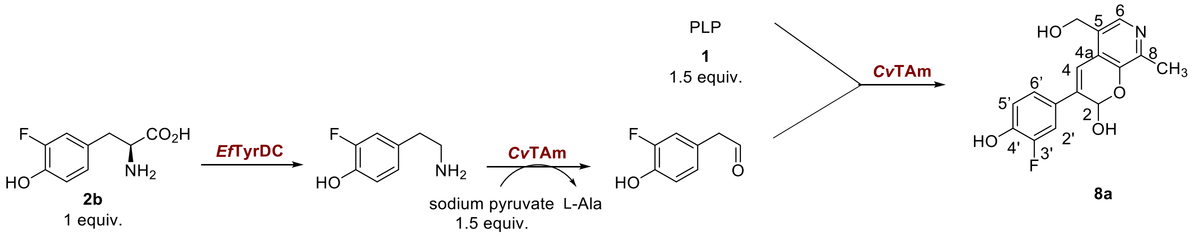

The reaction mixture (10 mL, pH 7.5) consisted of 50 mM HEPES, 10 mM 3-F-L-tyrosine **2b**, 15 mM PLP **1** and 10 mM sodium pyruvate. To initiate the reaction, 10% (v/v) of *Ef*TyrDC lysate and 10% (v/v) of *Cv*TAm lysate were added to the solution which was incubated at 37 °C, 250 rpm for 16 h. The reaction was quenched by adding 1% TFA. The product was purified using preparative HPLC (method 2, Supelco™ Discovery BIO wide pore (C18, 10 μm, 2.12 cm × 25 cm) preparative column, retention time: 16.2 min, run time: 28 mins, flow rate: 8 mL/min)). Fractions containing the desired product were freeze-dried to give product **8a** as a yellow powder (yield by HPLC (calibration curve) 51% (method 1, ACE 5 C18 column (150 × 4.6 mm), retention time: 5.2 min, run time: 10 mins, flow rate: 1 mL/min); final isolated yield 11.2 mg, 37%. ^{1}H NMR (600 MHz; CD_3OD) δ 8.21 (1H, s, py-6-H), 7.57 (1H, dd, *J* = 12 Hz, 2.4 Hz, 2'-H), 7.48 (1H, ddd, *J* = 8.4 Hz, 2.4 Hz, 0.6 Hz, 6'-H), 7.32 (1H, s, 4-H), 7.02 (1H, t, *J* = 8.4 Hz, 5'-H), A6.58 (1H, s, 2-H), 4.98-4.89 (2H, m, CH_2OH), 2.69 (3H, s, py-8-CH_3); ^{13}C NMR (151 MHz; CD_3OD) δ 152.5 ($^{1}J_{CF}$ = 241.6 Hz), 147.0, 142.9 ($^{2}J_{CF}$ = 15.1 Hz), 142.1, 138.1 ($^{3}J_{CF}$ = 4.5 Hz), 135.2, 134.8 ($^{4}J_{CF}$ = 1.5 Hz), 132.0, 128.4, 127.7 ($^{3}J_{CF}$ = 6.0 Hz), 119.3, 115.6 ($^{2}J_{CF}$ = 19.6 Hz), 112.7, 92.6, 59.3, 14.2; *m/z* [ES+] 304 ([M+H]$^+$, 100%); *m/z* [HRMS ES+] found [M+H]$^+$ 304.0979; [$C_{16}H_{14}FNO_4$+H]$^+$ requires 304.0980.

*3.10 Hydroxystyryl pyridine cascade 3: 3-Fluoro-5-(2-hydroxy-5-(hydroxymethyl)-8-methyl-2H-pyrano[2,3-c]pyridin-3-yl)benzene-1,2-diol **8b***

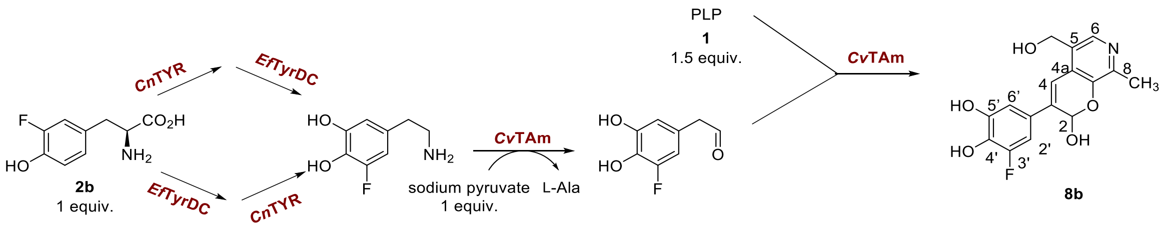

The reaction mixture (10 mL, pH 7.5) consisted of 50 mM HEPES, 10 mM 3-F-L-tyrosine **2b**, 5 μM $CuSO_4·5H_2O$, 15 mM PLP **1** and 10 mM sodium pyruvate. To initiate the reaction, 10% (v/v) of *Cn*TYR lysate, 10% (v/v) of *Ef*TyrDC lysate and 10% (v/v) of *Cv*TAm lysate were added to the solution which was then incubated at 37 °C, 250 rpm for 16 h. The reaction was quenched by adding 1% TFA. The product was purified using preparative HPLC (method 2, Supelco™ Discovery BIO wide pore (C18, 10 μm, 2.12 cm × 25 cm) preparative column, retention time: 15.5 min, run time: 28 mins, flow rate: 8 mL/min)). Fractions containing the desired product were freeze-dried to give product **8b** as an orange powder (yield by HPLC (calibration curve) 42% (method 1, ACE 5 C18 column (150 × 4.6 mm), retention time: 4.8 min, run time: 10 mins, flow rate: 1 mL/min); final isolated yield 11.2 mg, 35%. ^{1}H NMR (600 MHz; CD_3OD) δ 8.19 (1H, s, py-6-H), 7.24 (1H, s, 4-H), 7.08 (1H, dd, *J* = 12 Hz, 2.4 Hz, 2'-H), 7.05-7.04 (1H, m, 6'-H), 6.51 (1H, s, 2-H), 4.95-4.93 (2H, m, CH_2OH), 2.68 (3H, s, py-8-CH_3); ^{13}C NMR (151 MHz; CD_3OD) δ 153.3 ($^1J_{CF}$ = 226.5 Hz), 149.1 ($^3J_{CF}$ = 6.0 Hz), 147.0, 143.7, 142.9, 137.4 ($^2J_{CF}$ = 15.1 Hz), 135.1, 134.8, 132.0, 127.0 ($^3J_{CF}$ = 9.0 Hz), 112.8 ($^4J_{CF}$ = 1.5 Hz), 110.8, 107.1 ($^2J_{CF}$ = 21.1 Hz), 92.6, 59.3, 14.2; *m/z* [ES+] 320 ([M+H]$^+$, 100%); *m/z* [HRMS ES+] found [M+H]$^+$ 320.0929; [$C_{16}H_{14}FNO_5$+H]$^+$ requires 320.0929.

3.11 Hydroxystyryl pyridine cascade 4: 3-(3-Chloro-4-hydroxyphenyl)-5-(hydroxymethyl)-8-methyl-2H-pyrano[2,3-c]pyridin-2-ol ***8c***

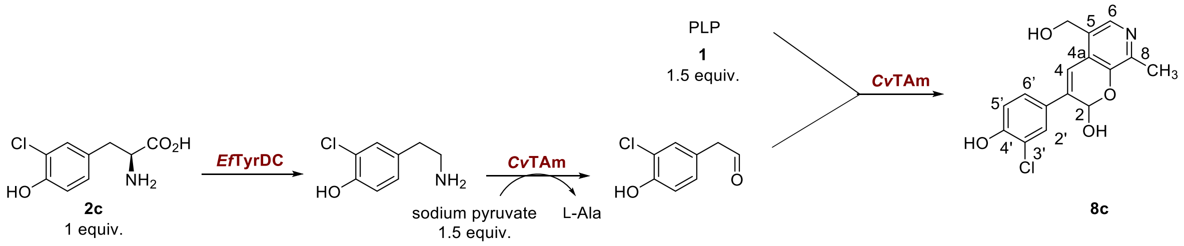

The reaction mixture (10 mL, pH 7.5) consisted of 50 mM HEPES, 10 mM 3-Cl-L-tyrosine **2c**, 15 mM PLP **1** and 10 mM sodium pyruvate. To initiate the reaction, 10% (v/v) of *Ef*TyrDC lysate and 10% (v/v) of *Cv*TAm lysate were added to the solution which was incubated at 37 °C, 250 rpm for 16 h. The reaction was quenched by adding 1% TFA. The product was purified using preparative HPLC (method 2, Supelco™ Discovery BIO wide pore (C18, 10 μm, 2.12 cm x 25 cm) preparative column, retention time: 18.1 min, run time: 28 mins, flow rate: 8 mL/min)). Fractions containing the desired product were freeze-dried to give product **8c** as a yellow powder (yield by HPLC (calibration curve) 48% (method 1, ACE 5 C18 column (150 × 4.6 mm), retention time: 5.5 min, run time: 10 mins, flow

rate: 1 mL/min); final isolated yield 11.6 mg, 36%. ^{1}H NMR (700 MHz; CD_3OD) δ 8.18 (1H, s, py-6-H), 7.78 (1H, d, *J* = 2.1 Hz, 2'-H), 7.60 (1H, dd, *J* = 8.7 Hz, 2.1 Hz, 6'-H), 7.31 (1H, s, 4-H), 7.01 (1H, d, *J* = 8.7 Hz, 5'-H), 6.54 (1H, s, 2-H), 4.95-4.88 (2H, m, C*H*$_2$OH), 2.67 (3H, s, py-8-CH_3); ^{13}C NMR (175 MHz; CD_3OD) δ 156.4, 146.7, 143.4, 142.5, 134.6, 133.8, 133.0, 129.5, 128.3, 127.7, 122.5, 117.9, 112.9, 92.3, 59.3, 14.6; *m/z* [ES+] 320 ([M+H]$^+$, 100%), *m/z* [HRMS ES+] found [M+H]$^+$ 320.0684; $[C_{16}H_{14}{}^{35}ClNO_4+H]^+$ requires 320.0682.

*3.12 Hydroxystyryl pyridine cascade 5: 3-(3-Iodo-4-hydroxyphenyl)-5-(hydroxymethyl)-8-methyl-2H-pyrano[2,3-c]pyridin-2-ol **8d***

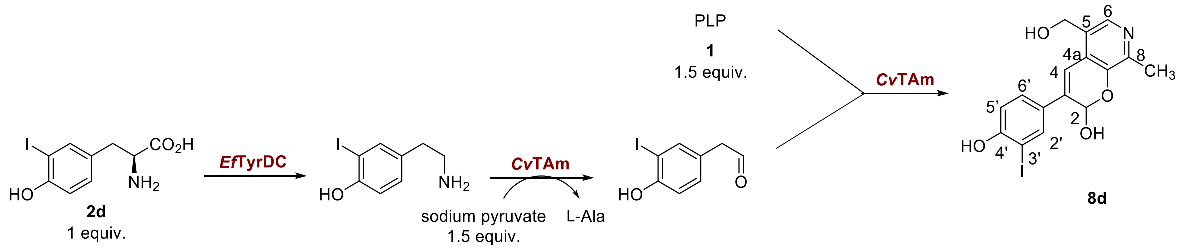

The reaction mixture (10 mL, pH 7.5) consisted of 50 mM HEPES, 10 mM 3-I-L-tyrosine **2d**, 15 mM PLP **1** and 10 mM sodium pyruvate. To initiate the reaction, 10% (v/v) of *Ef*TyrDC lysate and 10% (v/v) of *Cv*TAm lysates were added to the solution which was incubated at 37 °C, 250 rpm for 16 h. The reaction was quenched by adding 1% TFA. The product was purified using preparative HPLC (method 2, Supelco^{TM} Discovery BIO wide pore (C18, 10 µm, 2.12 cm x 25 cm) preparative column, retention time: 21.2 min, run time: 28 mins, flow rate: 8 mL/min)). Fractions containing the desired product were freeze-dried to give product **8d** as a yellow powder (yield by HPLC (calibration curve) 41% (method 1, ACE 5 C18 column (150 × 4.6 mm), retention time: 5.9 min, run time: 10 mins, flow rate: 1 mL/min); final isolated yield 12.1 mg, 29%. ^{1}H NMR (600 MHz; CD_3OD) δ 8.21 (1H, s, py-6-H), 8.16 (1H, d, *J* = 2.4 Hz, 2'-H), 7.67 (1H, dd, *J* = 8.4 Hz, 2.4 Hz, 6'-H), 7.30 (1H, s, 4-H), 6.94 (1H, d, *J* = 8.4 Hz, 5'-H), 6.56 (1H, s, 2-H), 4.98-4.88 (2H, m, C*H*$_2$OH), 2.69 (3H, s, py-8-CH_3); ^{13}C NMR (175 MHz; CD_3OD) δ 160.5, 147.0, 143.0, 142.8, 139.1, 135.9, 134.6, 132.0, 130.6, 129.5, 129.0, 116.0, 112.5, 92.5, 59.3, 14.3; *m/z* [ES+] 412 ([M+H]$^+$, 100%), *m/z* [HRMS ES+] found [M+H]$^+$ 412.0037; $[C_{16}H_{14}INO_4+H]^+$ requires 412.0040.

4 Single crystal X-ray diffraction studies

The diffraction data for compounds **3a-OEt** and **3b** were collected on a four-circle *Agilent SuperNova*

(Dual Source) single crystal X-ray diffractometer using a micro-focus CuK_α X-ray beam (λ = 1.54184 Å) and an *Atlas* CCD detector. The sample temperatures were controlled with an *Oxford Instruments* cryojet. All data were processed using the *CrysAlis^{Pro}* programme package from *Rigaku Oxford Diffraction*.[1] The crystal structures were solved with the *ShelXT* programme[2] and refined by least squares on the basis of F^2 with the *ShelXL* programme.[3] Both programmes were used within the *Olex^2* software suite.[4,5]

We note that only the crystal structure of **3b** passes the *checkCIF* validations for data completeness and consistency. The single crystals of **3a-OEt** were too small and diffracted only up to a data resolution of 0.99 Å. And since the collection of a full data set was not possible, and the available data was only used to guide NMR data interpretation.

4.1 Crystal structure refinement process for ***3a-OEt***

All non-hydrogen atoms were refined anisotropically by the full-matrix least-squares method. Hydrogen atoms affiliated with oxygen and nitrogen atoms were refined isotropically [U_{iso}(H) = 1.5U_{eq}(O)] in geometrically constrained positions. Hydrogen atoms associated with carbon and nitrogen atoms were refined isotropically [U_{iso}(H) = 1.2U_{eq}(C/N)] in geometrically constrained positions.

The crystal structure of **3a** is shown in Figure S5, and its crystallographic and refinement parameters are shown in Table S1.

4.2 Crystal structure refinement process for ***3b***

All non-hydrogen atoms were refined anisotropically by the full-matrix least-squares method. The locations of the hydrogen atoms affiliated with the N1 nitrogen atom and the C10 carbon atom were identified from the difference map and were refined isotropically [U_{iso}(N–**H**) = 1.2U_{eq}(N) and U_{iso}(C–**H**) = 1.2U_{eq}(C)], as calculated positions resulted in molecular geometries with an unreasonably short C−H···H-O contact. All other hydrogen atoms associated with carbon atoms were refined isotropically [U_{iso}(H) = 1.2U_{eq}(C)] in geometrically constrained positions.

The structure of the disordered trifluoroacetate anion in the major occupancy site was modelled using rigid-body fragment fitting (with the FRAG and FEND commands in *SHELX*[3]). The structures of the anion in the other two minor occupancy sites were refined using the SAME restraints command

in *SHELX*.[3]

The crystal structure of **3b** is shown in Figure S6. The crystallographic and refinement parameters are shown in Table S1.

Table S1. Crystal data and structure refinement for compounds **3a-OEt** and **3b**.

Identification code	**3a**	**3b**
Empirical formula	$C_{40}H_{40}F_6N_2O_{14}$	$C_{18}H_{16}F_3NO_5$
Formula weight	886.74	383.37
Temperature/K	150.01(10)	150.01(10)
Crystal system	triclinic	monoclinic
Space group	*P*-1	$P2_1/n$
a / Å	7.3439(13)	4.38440(10)
b / Å	14.008(3)	16.8800(3)
c / Å	21.309(5)	23.4788(4)
α / °	102.02(2)	90
β / °	96.823(17)	91.0715(19)
γ / °	103.241(18)	90
V / Å^3	2054.9(8)	1737.34(6)
Z	2	4
ρ_{calc} / g cm^{-3}	1.433	1.465
μ / mm^{-1}	1.091	1.109
F(000)	920.0	792.1
Radiation	CuK_α (λ = 1.54184)	CuK_α (λ = 1.54184)
2Θ range for data collection / °	19.548 − 102.13	3.225° − 66.591°
Index ranges	$-4 \leqslant h \leqslant 7$<br>$-13 \leqslant k \leqslant 14$<br>$-21 \leqslant l \leqslant 14$	$-5 \leqslant h \leqslant 5$<br>$-20 \leqslant k \leqslant 20$<br>$-27 \leqslant l \leqslant 27$
Reflections collected	3741	26141
Independent reflections	3464 [R_{int} = 0.0461]	3070 [R_{int} = 0.0348]
Data/restraints/parameters	3464 / 12 / 573	2748 / 46 / 283
Goodness-of-fit on F^2	1.208	1.094
Final *R* indexes [$I \geqslant 2\sigma(I)$]	R_1 = 0.1486, wR_2 = 0.3441	R_1 = 0.0450, wR_2 = 0.1100
Final *R* indexes [all data]	R_1 = 0.2166, wR_2 = 0.4018	R_1 = 0.0501, wR_2 = 0.1134
Largest diff. peak/hole / e Å^{-3}	0.49 / −0.48	0.232 / −0.313
CCDC deposition number	−	2271746

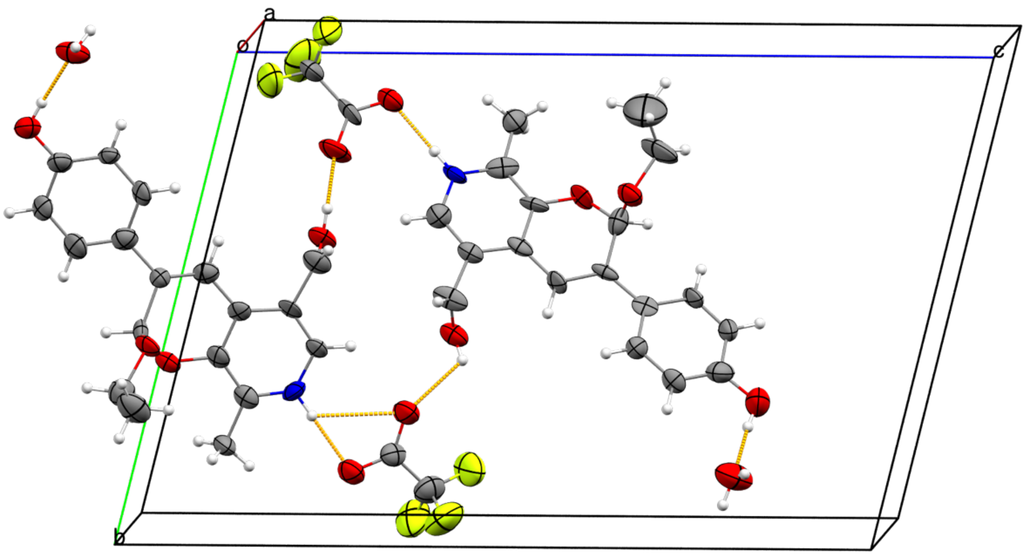

Figure S5. Single crystal X-ray structure of compound **3a**. (colour scheme: grey – carbon, red – oxygen, blue – nitrogen, green – fluorine; white – hydrogen; orange lines – hydrogen bonds). The thermal ellipsoids are draws at the 50% probability level.

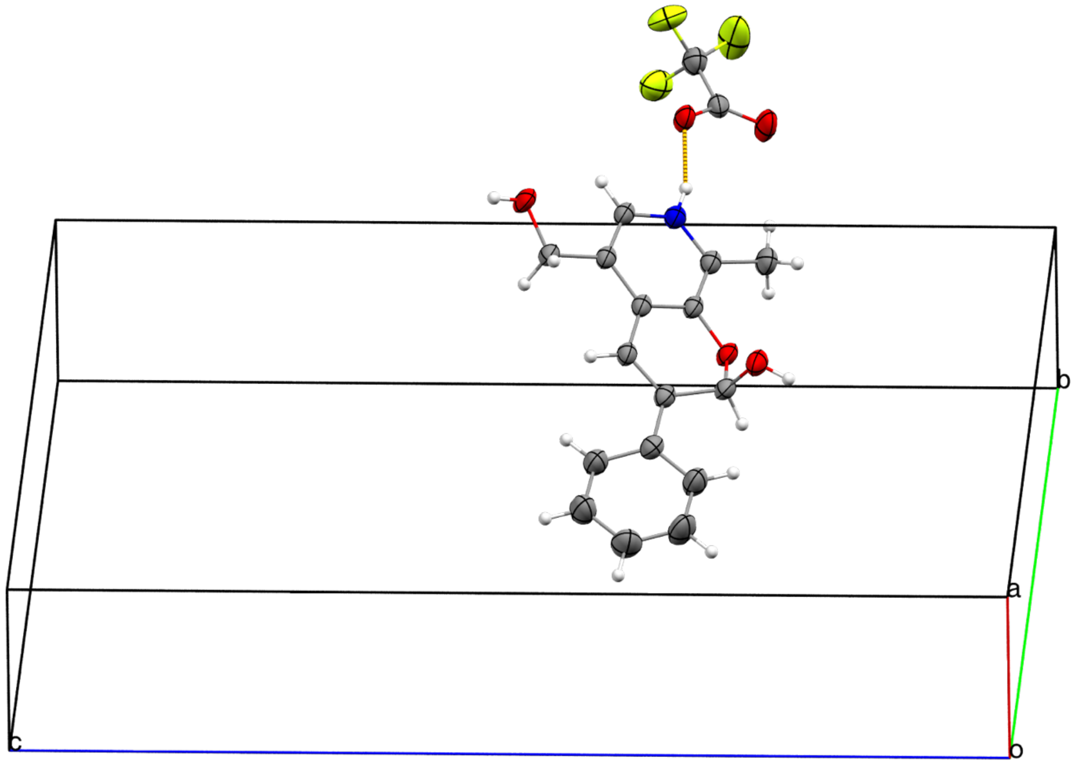

Figure S6. Single crystal X-ray structure of compound **3b**. Colour scheme: grey – carbon, red – oxygen, blue – nitrogen, yellow – fluorine, white – hydrogen; orange lines – hydrogen bonds). The thermal ellipsoids are draws at the 50% probability level.

5. AutoDock of styryl pyridines with transaminases

Docking studies of styryl pyridines with different transaminases was performed by Autodock Vina (v.1.2.0).[6,7] Docking results (binding modes) were viewed with UCSF ChimeraX.[8,9]

Table S2. Global docking of **3b** with *Cv*TAm.

Ligand and Enzyme	Affinity (kcal/mol)	Ranking[a]	In the cliff between two subunits
**3b** with *Cv*TAm (PDB: 4BA4)[10]	-7.1	1	Yes
	-7.0	2	Yes
	-6.9	3	Yes
	-6.8	4	Yes
	-6.6	5	No
	-6.6	6	Yes
	-6.3	7	No
	-6.2	8	No
	-6.0	9	No

a. The ranking order followed the affinity energy.

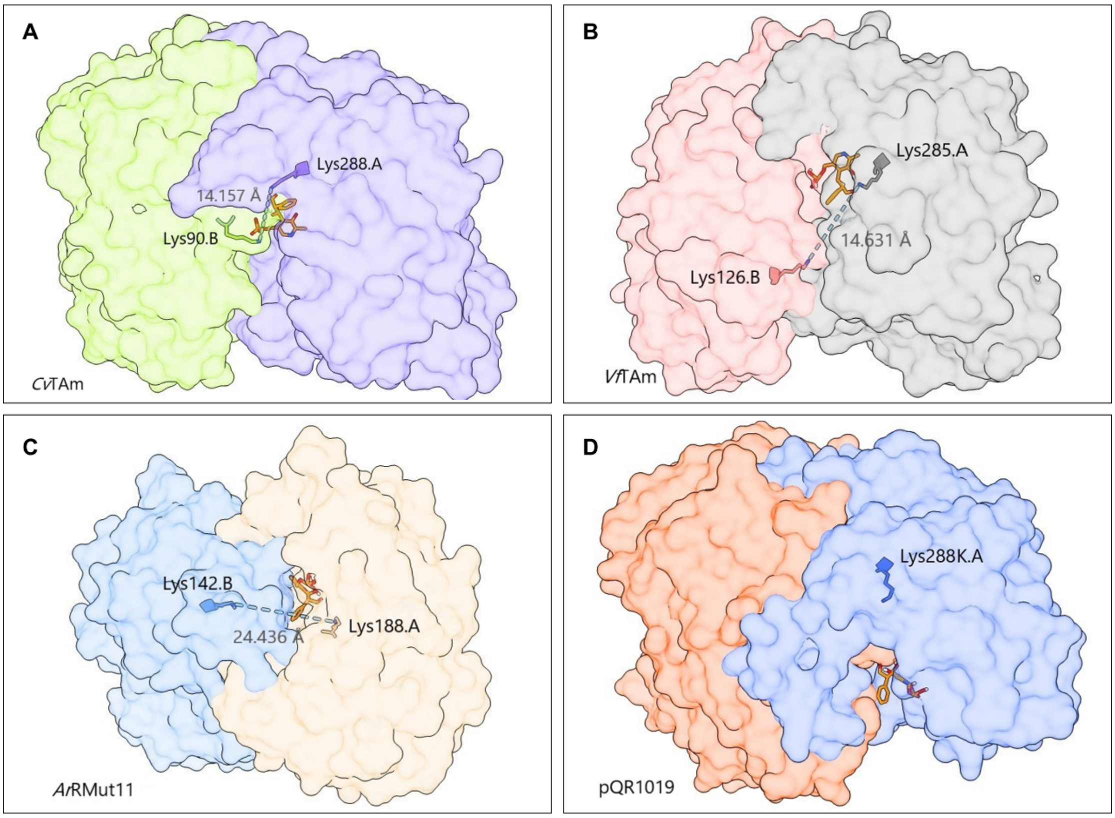

Figure S7. Docking of hydroxystyryl pyridine **3a** with different transaminases and the distances of two lysine residues. **A.** Docking of **3a** with *Cv*TAm (PDB: 4BA4)[10]. The distances of Lys288.A and Lys90.B are 14.157 Å. **B.** Docking of **3a** with *Vf*TAm (PDB: 5ZTX)[11]. The distances of Lys285.A and Lys126.B are 14.631 Å. **C.** Docking of **3a** with *Ar*Rmut11 (PDB: 3WWJ)[12]. The distances of Lys188.A and Lys142.B are 24.436 Å. **D.** Docking of **3a** with pQR1019-*Kp*TAm (PDB: 3I5T)[13].No equivalent lysine residue was found on the subunit B. Figures were generated using UCSF ChimeraX.[8,9]

6. Molecular dynamic simulations and MM-PBSA calculation

To investigate *in silico* whether the hydroxystyryl pyridines generated had the potential to inhibit human pancreatic amylase (HPA), molecular dynamics modeling of **3a** and HPA (PDB: 2QMK44) was conducted with GROMACS 2020.4,45-47 using the AMBER99SB-ILDN forcefield to investigate the structural flexibility of predicted protein-ligand complexes.48 As comparisons, the natural HPA inhibitor montbretin A (MbA, PDB: 4W93)49 and dehydrodieugenol B (DDEB)50 were also investigated. A 30 ns molecular dynamics simulation for each ligand was performed in triplicate on the entire system at 300 K. Root-mean-square deviation (RMSD) of the backbone group was calculated using the initial structure as a reference to assess structural stability, and the molecular mechanics Generalised Born surface area (MM-GBSA) method was employed to investigate changes in binding free energy within a protein-ligand system. According to the dynamic simulation, **3a** bound to HPA tightly and the RMSD fluctuated between 0.1 and 0.2 with the totalΔGBSA at −29.56 (Figure S8A). DDEB can bind to HPA with a slightly higher fluctuation between 0.1 and 0.25, and the total ΔGBSA was −18.62 (Figure S8B). Interestingly, MbA dissociated from HPA after 1 ns and the ΔGBSA reached up to 10.76 (Figure S8C and movie 3). These dynamic simulations indicated that the hydroxystyryl pyridine 3a could potentially be an inhibitor of such amylases.

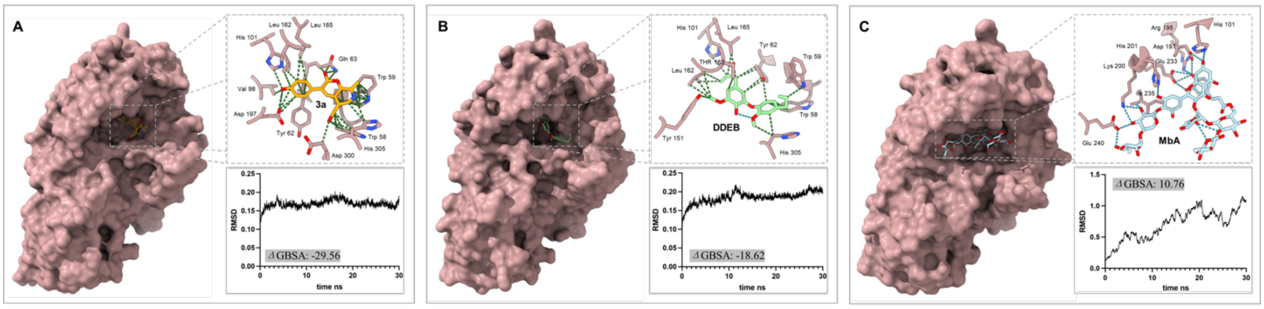

Figure S8. Dynamic simulation of ligands (**3a**, DDEB AND MbA) with HPA. A. Dynamic simulation of 3a with HPA. B. Dynamic simulation of DDEB with HPA with DDEB fitted into the active site of HPA. C. Dynamic simulation of MbA with HPA.

Molecular dynamic simulation software GROMACS 2020.4 with AMBER99SB-ILDN forcefield[14-17] was used to investigate the structural flexibility of predicted protein-ligand complexes. The complex topology files were prepared using Ambertools and ACPYPE.[18] The starting structure for the molecular dynamic simulation was solvated in a cubic simulation box with water and neutralized using an adequate amount of Na^+. The entire system was energy-minimized using the steepest descent method (2000 steps) followed by the conjugate gradient method (5000 steps). Two-phase equilibration was carried out under the NVT and NPT ensembles for 50 ns each. Finally, a 30-ns molecular dynamics simulation was performed in triplicate on the entire system at 300 K. RMSD of the backbone group was calculated using the initial structure as a reference to assess structural stability.

Molecular mechanics Generalised Born surface area (MM-GBSA) method was employed to investigate changes in binding free energy within a protein-ligand system. Trajectories derived from molecular dynamics simulations were analysed using the gmx_MMPBSA tool[19] after the elimination of PBC conditions. The per-residue effective free energy decomposition (prEFED) protocol was utilized to identify energetically significant residues located within 4 Å of the protein-ligand interface. The AMBER99SB force field was utilized to compute the internal energy term (ΔEint) as well as the

van der Waals (ΔEvdW) and electrostatic (ΔEele) energies (Table S3).

Table S3. MM-GBSA analysis for HPA-ligand complex.

a. HPA-3a complex

Delta (Complex - Receptor - Ligand):

Energy Component	Average	SD(Prop.)	SD
ΔBOND	-0.00	2.69	0.00
ΔANGLE	0.00	5.25	0.00
ΔDIHED	0.00	1.02	0.00
ΔVDWAALS	-31.43	0.51	3.25
ΔEEL	-29.41	0.13	4.18
Δ1-4 VDW	-0.00	2.19	0.00
Δ1-4 EEL	-0.00	0.61	0.00
ΔEGB	35.21	0.33	2.58
ΔESURF	-3.94	0.02	0.13
ΔGGAS	-60.84	0.53	4.86
ΔGSOLV	31.27	0.33	2.53
ΔTOTAL	-29.56	0.63	2.68

b. HPA-DDEB complex

Delta (Complex - Receptor - Ligand):

Energy Component	Average	SD(Prop.)	SD
ΔBOND	0.00	1.60	0.00
ΔANGLE	0.00	2.52	0.00
ΔDIHED	-0.00	2.13	0.00
ΔVDWAALS	-29.55	0.81	1.71
ΔEEL	-8.09	1.17	1.92
Δ1-4 VDW	-0.00	0.78	0.00
Δ1-4 EEL	0.00	0.36	0.00
ΔEGB	23.61	0.14	1.96
ΔESURF	-4.59	0.06	0.17
ΔGGAS	-37.63	1.42	3.01
ΔGSOLV	19.02	0.15	1.86
ΔTOTAL	-18.62	1.43	1.87

c. HPA-MbA complex

Delta (Complex - Receptor - Ligand):

Energy Component	Average	SD(Prop.)	SD
ΔBOND	0.00	6.99	0.00
ΔANGLE	-0.00	4.49	0.00
ΔDIHED	-0.00	2.62	0.00
ΔVDWAALS	-2.02	0.56	0.85
ΔEEL	565.92	14.56	49.22
Δ1-4 VDW	0.00	1.86	0.00
Δ1-4 EEL	-0.00	4.05	0.00
ΔEGB	-553.06	22.82	45.48
ΔESURF	-0.09	0.03	0.05
ΔGGAS	563.90	14.57	48.47
ΔGSOLV	-553.14	22.82	45.50
ΔTOTAL	10.76	27.07	3.28

7. Kinetic studies with CvTAm

Different equivalents of pyruvate (0.1, 0.5, 1, 1.5, 2 eq.) to **4a** (1 eq., 10 mM) were tested with purified wildtype *Cv*TAm (0.1 mg/mL) and **1** was used at 1 mM (0.1 eq.) and 15 mM (1.5 eq.). As shown in Figure S8, when using 1 mM **1**, yields of **5a** increased with increasing amounts of pyruvate and reached the maximum yield (93%) at 1 eq. of pyruvate. No **3a** was generated in the reactions. When using 15 mM **1**, yields of both **5a** and **3a** increased with increasing amounts of pyruvate and reached the maximum yield at 1 eq. of pyruvate (47% for **3a** and 41% of **5a**, Figure S9).

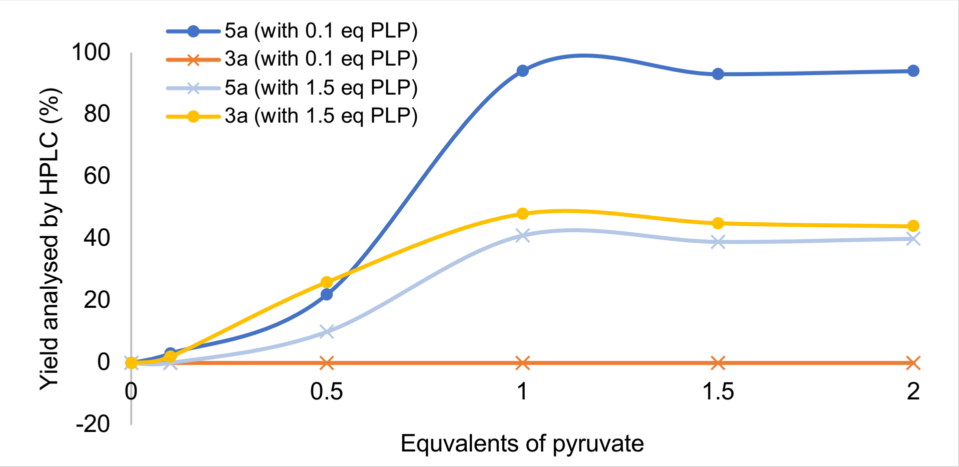

Figure S9. Production of **3a** and **5a** using different equivalents of pyruvate. Reactions were performed with **4a** (10 mM, 1 eq,), sodium pyruvate (0.1-2 eq,), **1** (0.1 and 1.5 eq.) and purified *Cv*TAm (0.1 mg/mL) in HEPES buffer (50 mM, pH 7.5) for 16 h. The yield of **5a** refers to the yield at equilibrium.

Different ratios of **1** (0 - 2.5 eq.) to **4a** (1 eq., 10 mM) were also tested with purified wildtype *Cv*TAm (0.1 mg/mL). With lower equivalents of **1** (<0.1 eq.), only the aldol product **3a** was observed. The product **3b** was formed at higher equivalents of PLP **1** (>0.2 eq.) and reached a maximum at 1.5 eq. of **1** (48% yield by HPLC analysis, Figure S10).

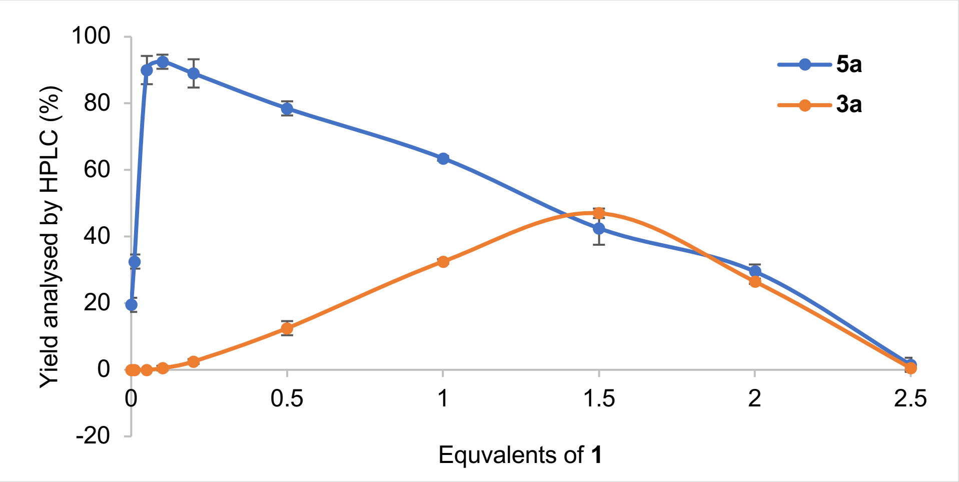

Figure S10. Production of **3a** and **5a** using different equivalents of PLP **1**. Reactions were performed with **4a** (10 mM, 1 eq,), sodium pyruvate (1 eq,), **1** (0 - 2.5 eq.) and purified *Cv*TAm (0.1 mg/mL) in HEPES buffer (50 mM, pH 7.5) for 16 h. The yield of **5a** refers to the yield at equilibrium.

For the kinetic study of **transamination activities** (here conversion of the amine to the aldehyde), the concentration of **4a** was varied from 0.5 mM to 20 mM (1 eq.). Reactions contain 1 eq. of pyruvate and 0.1 eq. of **1** in HEPES buffer (50 mM, pH 7.5) and purified *Cv*TAm at a final concentration of 10 μg/mL was used. Reactions were performed at 37 °C, 700 rpm. Samples of each reaction were obtained at 2 min, 5 min, 10 min and 20 min, and quenched by flash freeze-drying. Samples were then measurement by HPLC at 280 nm. The apparent $K_{m.app}$ is 1.69 mM and $k_{cat.app}$ is 6.04 s^{-1}, giving $k_{cat.app}/K_{m.app}$ = 3.57 s^{-1} mM^{-1}.

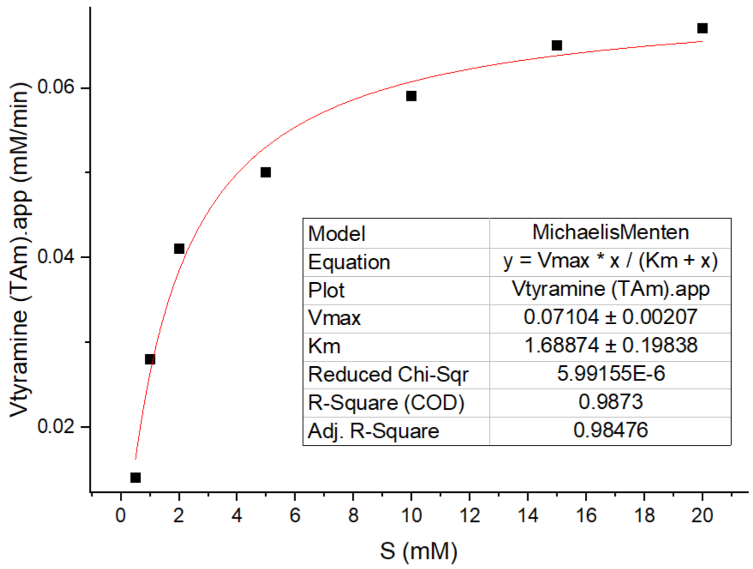

For the kinetic study of aldol addition activities, the concentration of **4a** was varied from 0.1 mM to 15 mM (1 eq.). Reactions contained 1 eq. of pyruvate and 1.5 eq. of **1** in HEPES buffer (50 mM, pH 7.5) and purified *Cv*TAm at a final concentration of 10 μg/mL was used. Reactions were performed at 37 °C, 700 rpm. Samples of each reaction were obtained at 2 min, 5 min, 10 min, 20 min, 30 min and 40 min, and quenched by flash freeze-drying. Samples were then measurement by HPLC at 280 nm. The apparent $K_{m.app}$ is 9.84 mM and $k_{cat.app}$ is 1.75 s^{-1}, giving $k_{cat.app}/K_{m.app}$ = 0.18 s^{-1} mM^{-1}.

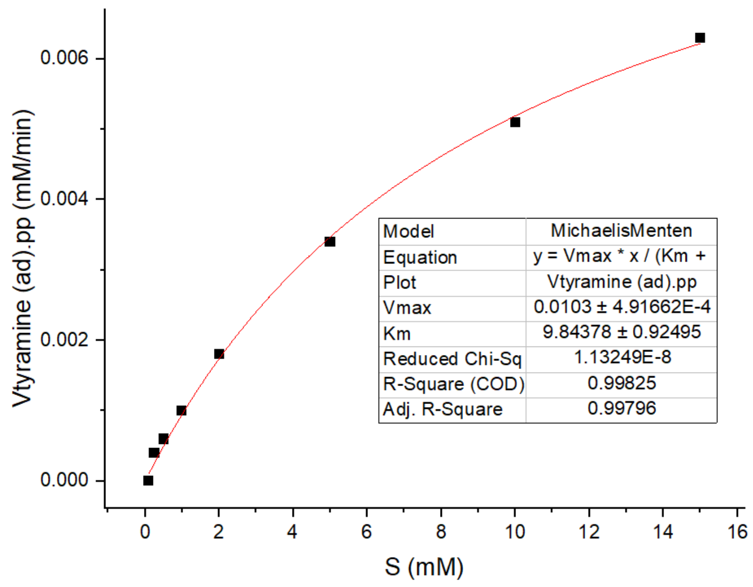

References

1 *CrysAllisPro*. Agilent Technologies, Inc. **2013**.

2 G. M Sheldrick, *Acta Crystallogr A.,* 2015, **64**, 3-8.

3 G. M. Sheldrick, *Acta Crystallogr C*., 2015, ***71***, 3-8.

4 O. V. Dolomanov,L. J. Bourhis, R. J. Gildea, J. A. K. Howard and H. Puschmann, *J. Appl. Crystallogr.,* 2009, **42**, 339-341.

5 C. B. Hübschle, G. M. Sheldrick and B. Dittrich, *J. Appl. Crystallogr.,* 2011, **44**, 1281-1284.

6 J. Eberhardt, D. Santos-Martins, A. F. Tillack and S. Forli, *J. Chem. Inf. Model*., 2021, **61**, 3891-3898.

7 O. Trott and A. J. Olson, *J. Comput. Chem*., 2010, **31**, 455-461.

8 E. F. Pettersen, T. D. Goddard, C. C. Huang, E. C. Meng, G. S. Couch, T. I. Croll, J. H. Morris and T. E. Ferrin, *Protein Sci*., 2021, **30**, 70-82.

9 T. D. Goddard, C. C. Huang, E. C. Meng, E. F. Pettersen, G. S. Couch, J. H. Morris and T. E. Ferrin, *Protein Sci*., 2018, **27**, 14-25.

10 C. Sayer, M. N. Isupov, A. Westlake A, J. A. Littlechild, *Acta Crystallogr D Biol Crystallogr*., 2013, **69**, 564-576.

11 Y. C. Shin, H. Yun and H. H. Park, *Sci. Rep.,* 2018, **8**, 11454.

12 L. J. Guan, J. Ohtsuka, M. Okai, T. Miyakawa, T. Mase, Y. Zhi, F. Hou, N. Ito, A. Iwasaki, Y. Yasohara and M. Tanokura, *Sci. Rep.,* 2015, **5**, 10753.

13 Y. Patskovsky, R. Toro, J. Freeman, J. Do, J. M. Sauder, S. K. Burley and S. C. Almo, RCSB Protein Data Bank. https://www.rcsb.org/structure/3I5T (accessed Feb 04, 2023)

14 E. Lindahl, M. J. Abraham, B. Hess and D. van der Spoel, *GROMACS 2020.4 Manual*. Zenodo: 2020. https://doi.org/10.5281/zenodo.4054996.

15 H. J .C. Berendsen, D. van der Spoel and R. van Drunen, *Comp. Phys. Comm.,* 1995, **91**, 43-56.

16 M. J. Abraham, T. Murtola, R. Schulz, S. Páll, J. C. Smith, B. Hess and E. Lindahl, *SoftwareX*., 2015, **1-2**, 19-25.

17 K. Lindorff-Larsen, S. Piana, K. Palmo, P. Maragakis, J. L. Klepeis, R. O. Dror and D. E. Shaw, *Proteins.,* 2010, **78**, 1950-1958.

18 A. W. Sousa da Silva and W. F. Vranken, *BMC Res Notes*., 2012, **5**, 367.

19 M. S. Valdés-Tresanco, M. E. Valdés-Tresanco, P. A. Valiente and E. Moreno, *J. Chem. Theory Comput*., 2021, **17**, 6281-6291.

Appendix

Analytical HPLC traces for styryl pyridine products

3a

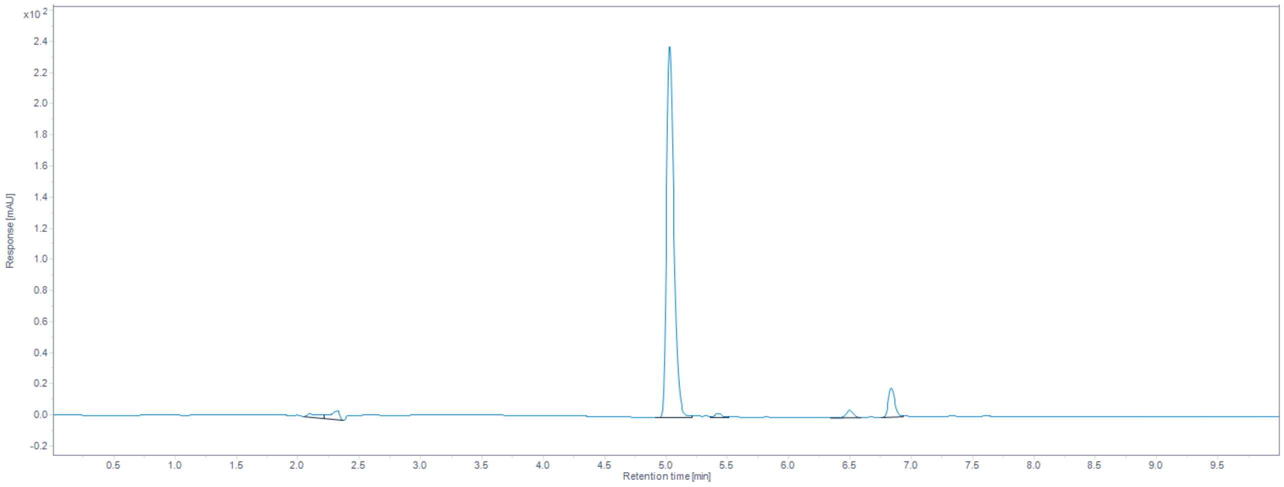

3b

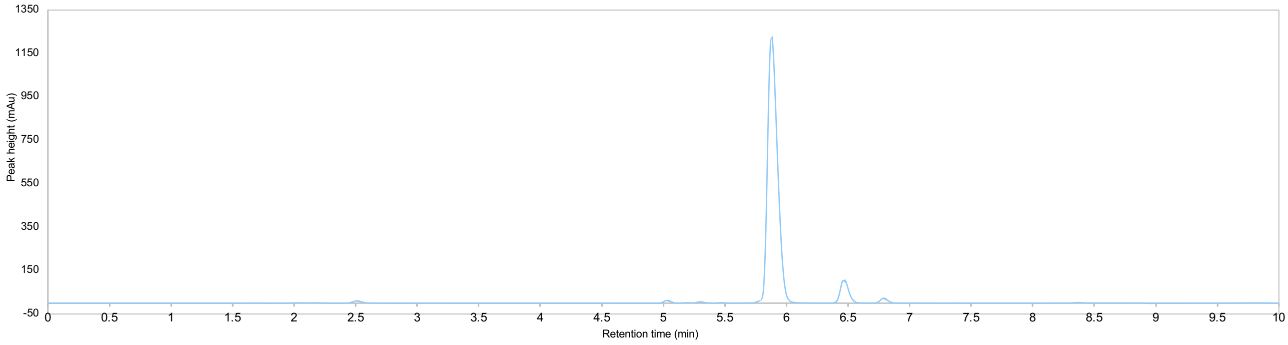

3e

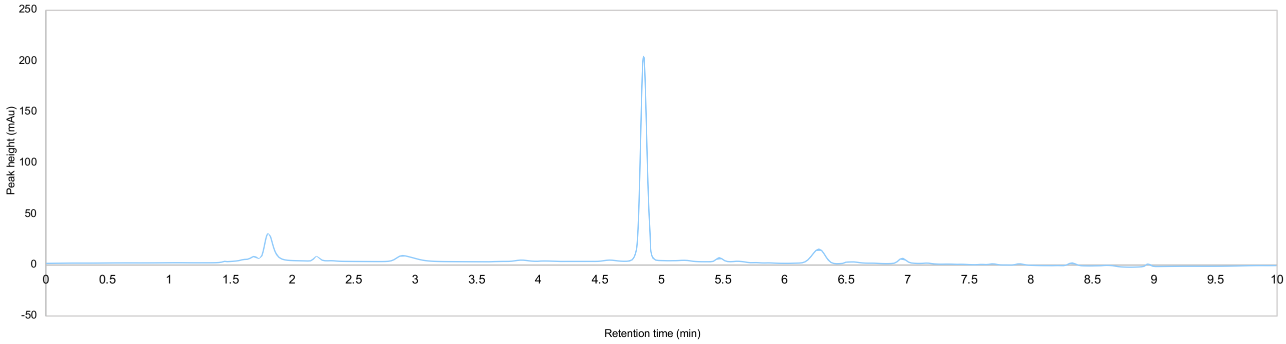

7a

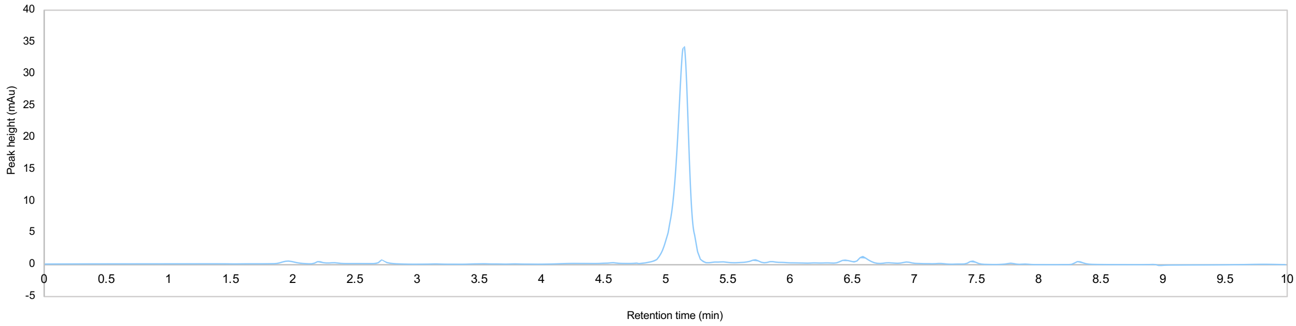

7b

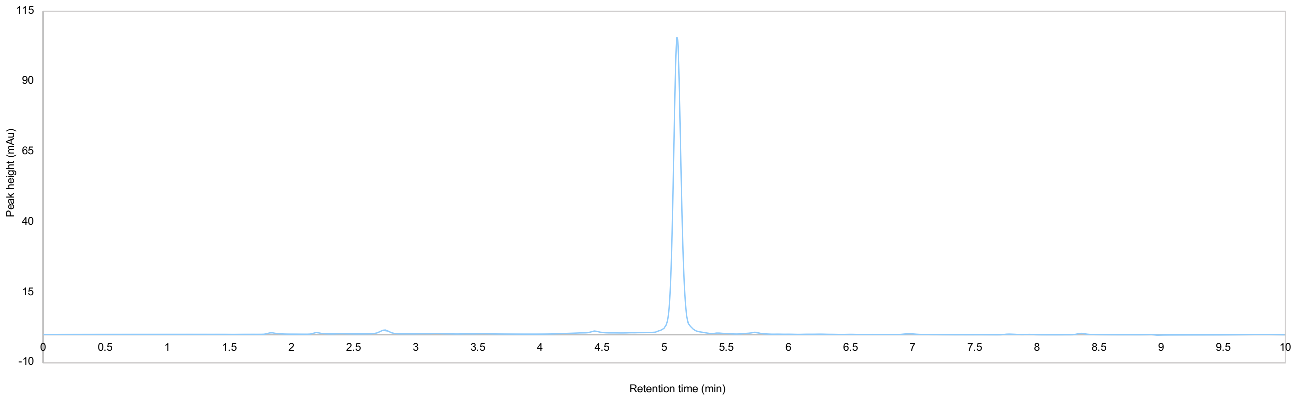

Peak height (mAu)
Retention time (min)

7c

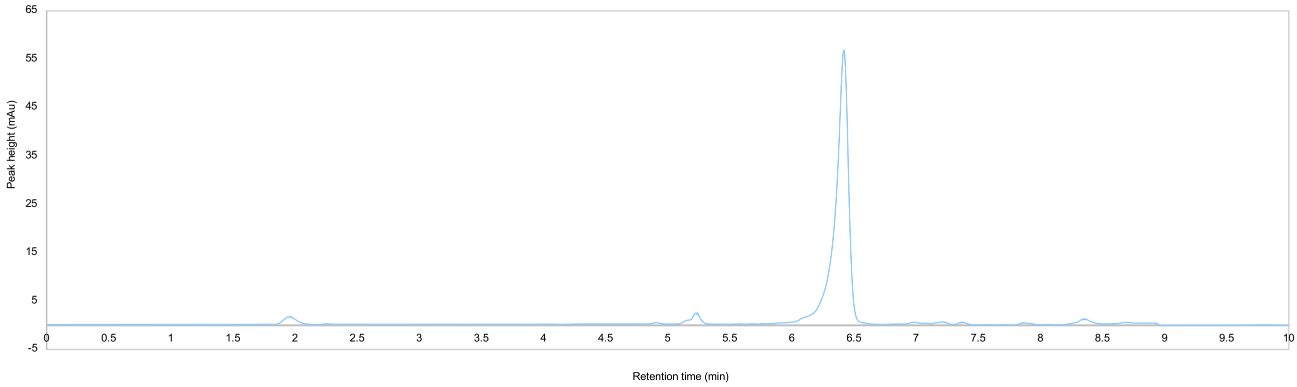

Peak height (mAu)
Retention time (min)

7d

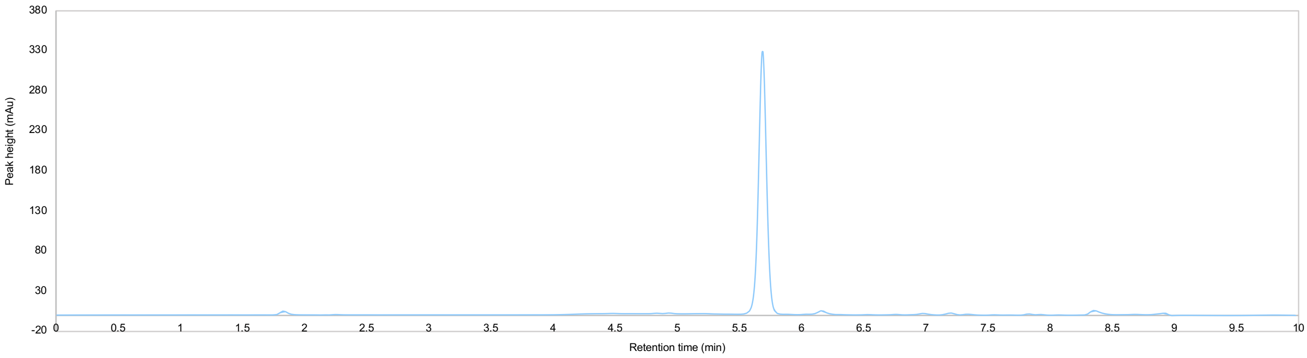

Peak height (mAu)
Retention time (min)

8a

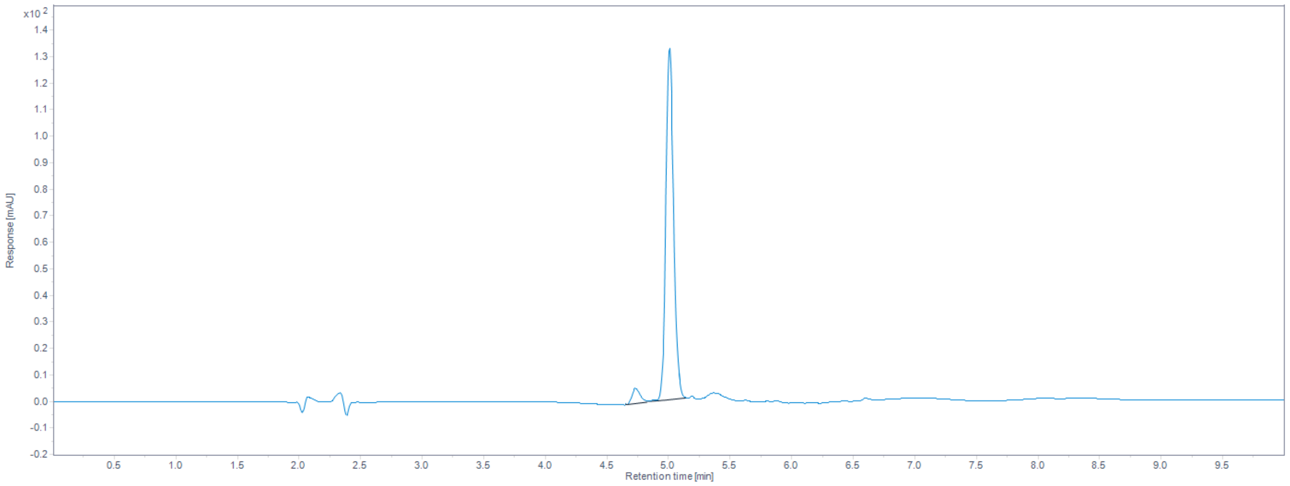

Response [mAU]
Retention time [min]

8b

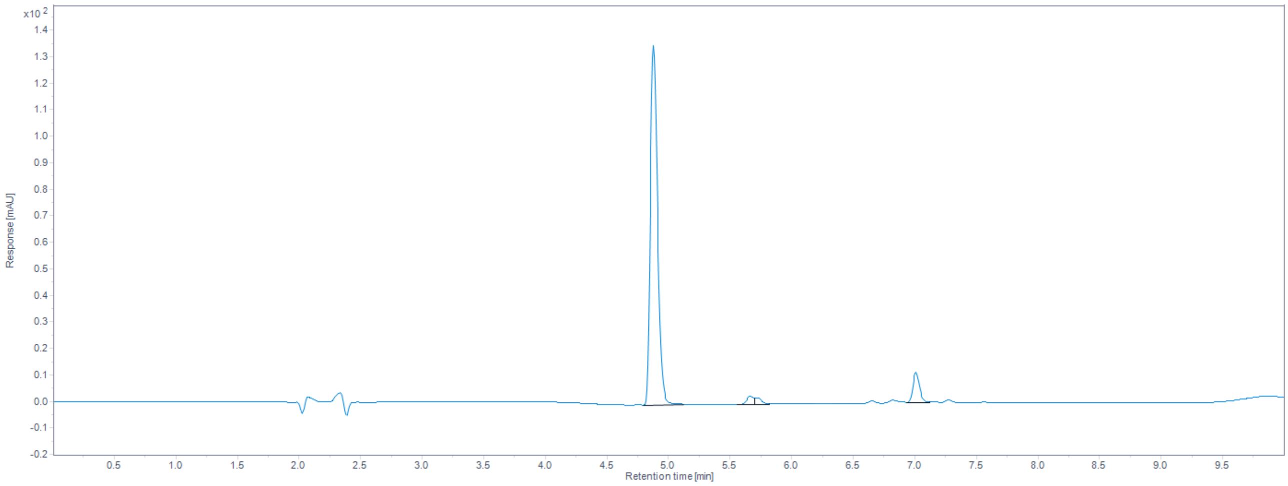
x10 2
Response [mAU]
Retention time [min]

8c

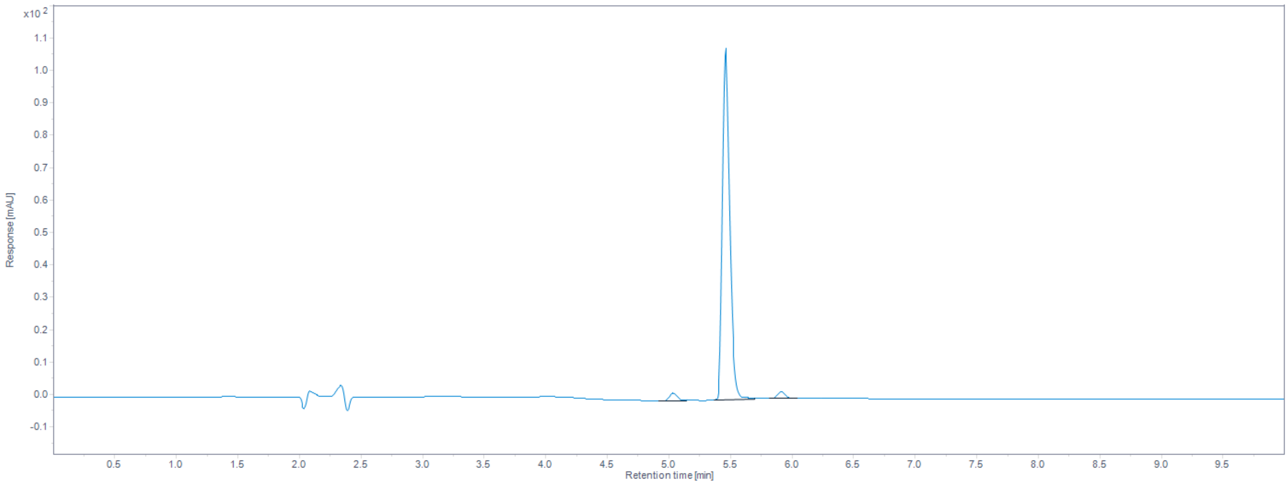
x10 2
Response [mAU]
Retention time [min]

8d

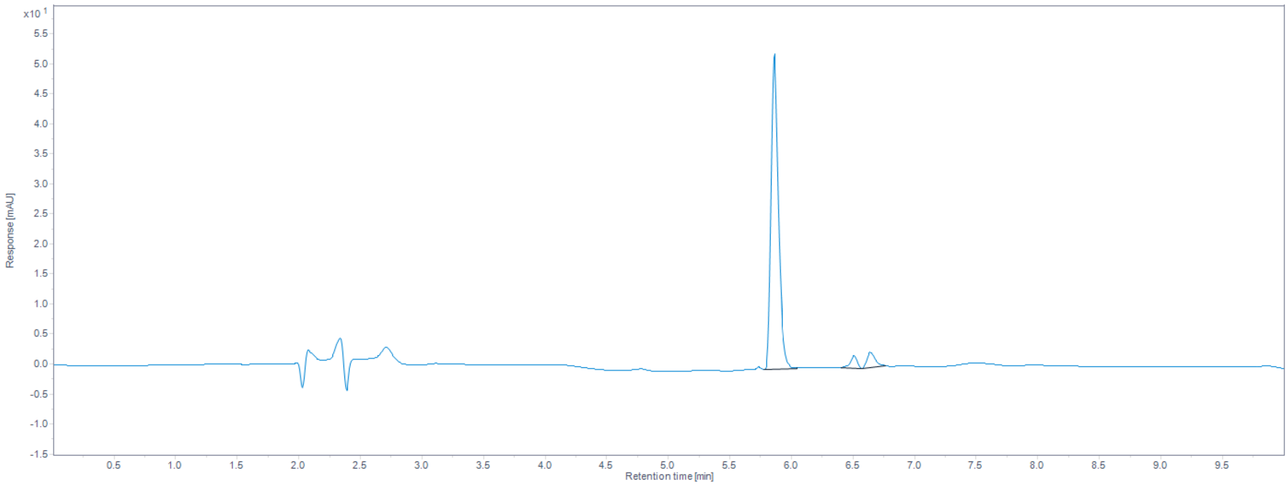
x10 1
Response [mAU]
Retention time [min]

NMR Spectroscopic data

3a

^{1}H NMR (700 MHz; CD_3OD)

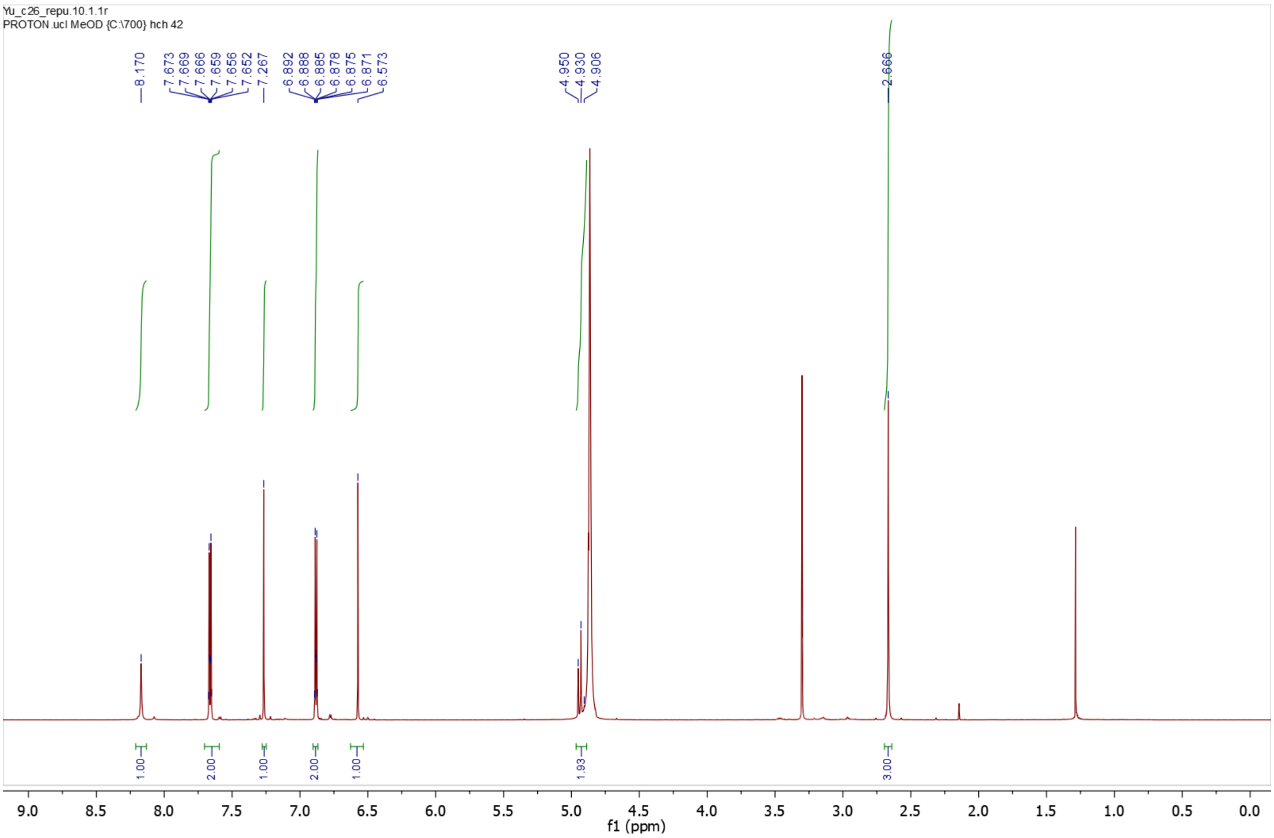

^{13}C NMR (175 MHz; CD_3OD)

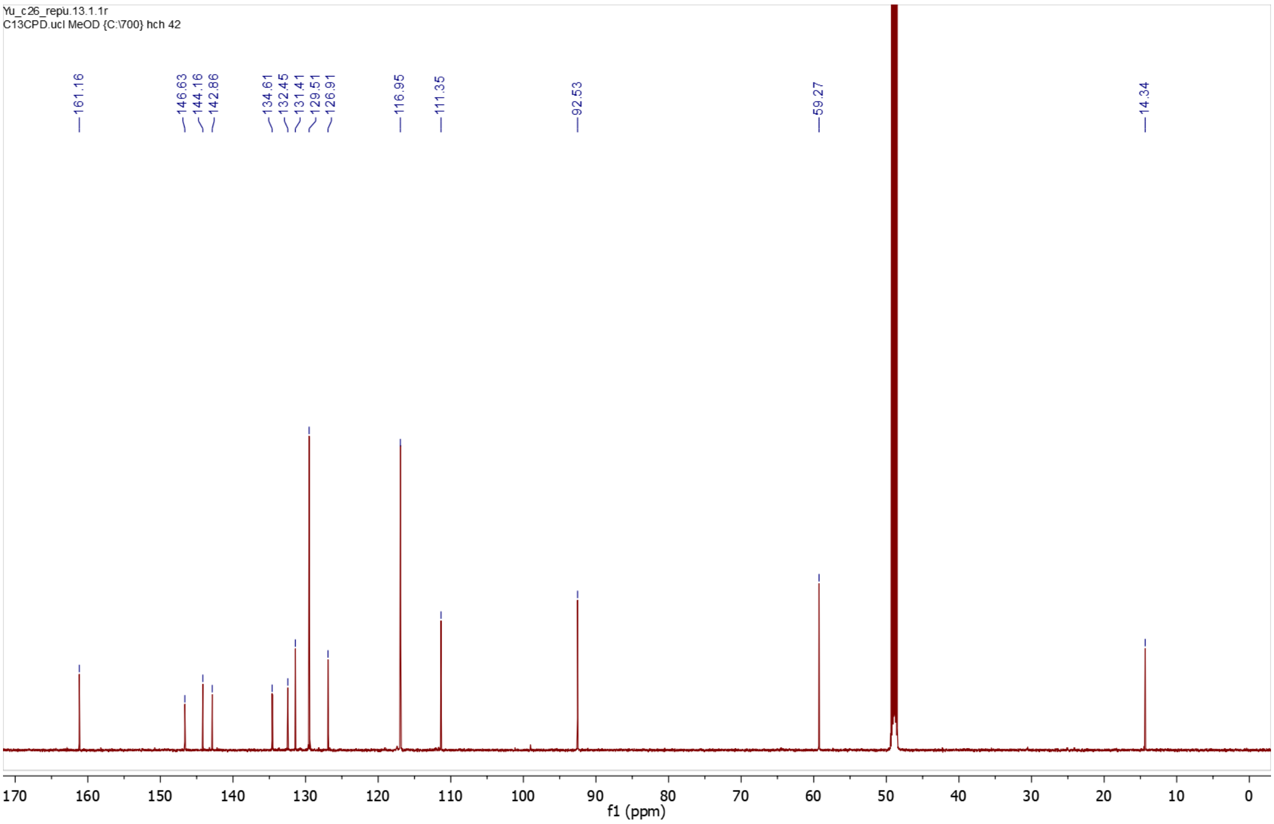

3b

^{1}H NMR (600 MHz; CD_3OD)

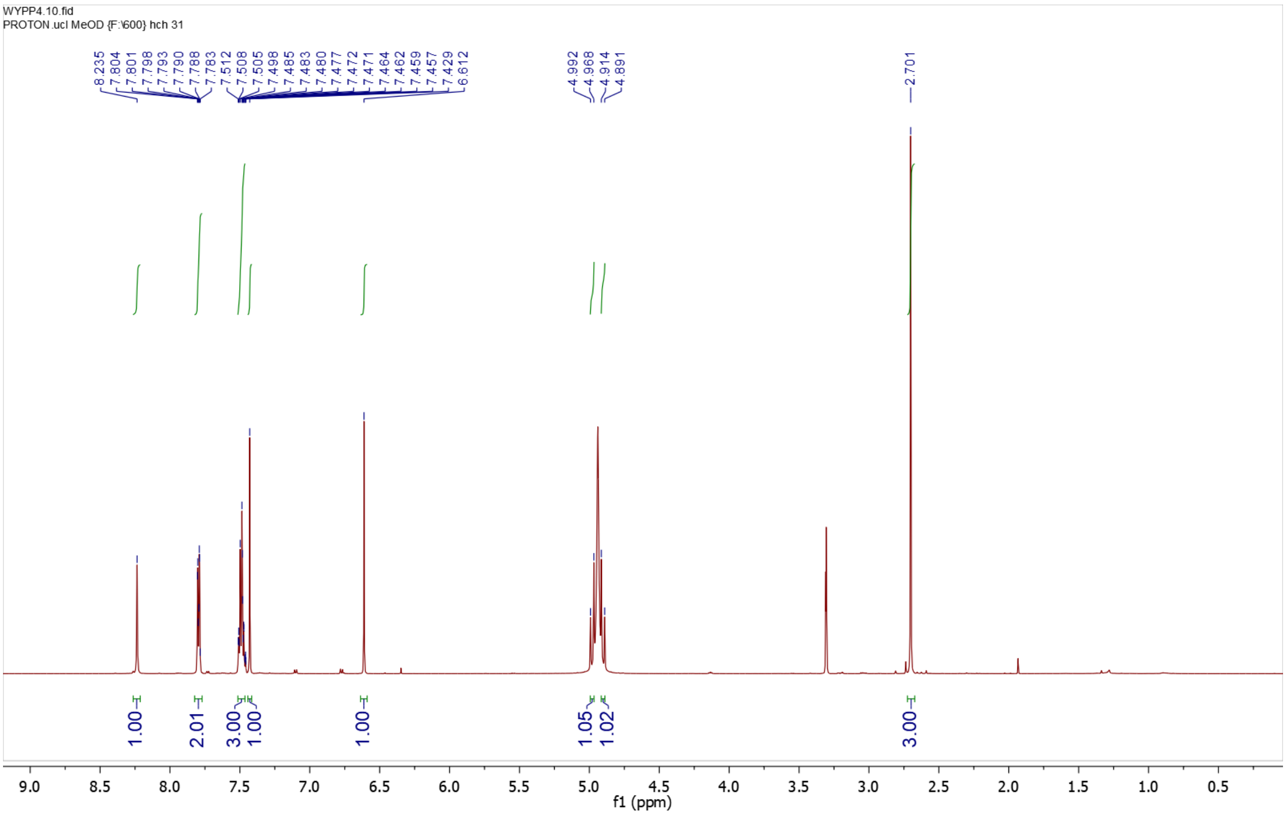

^{13}C NMR (151 MHz; CD_3OD)

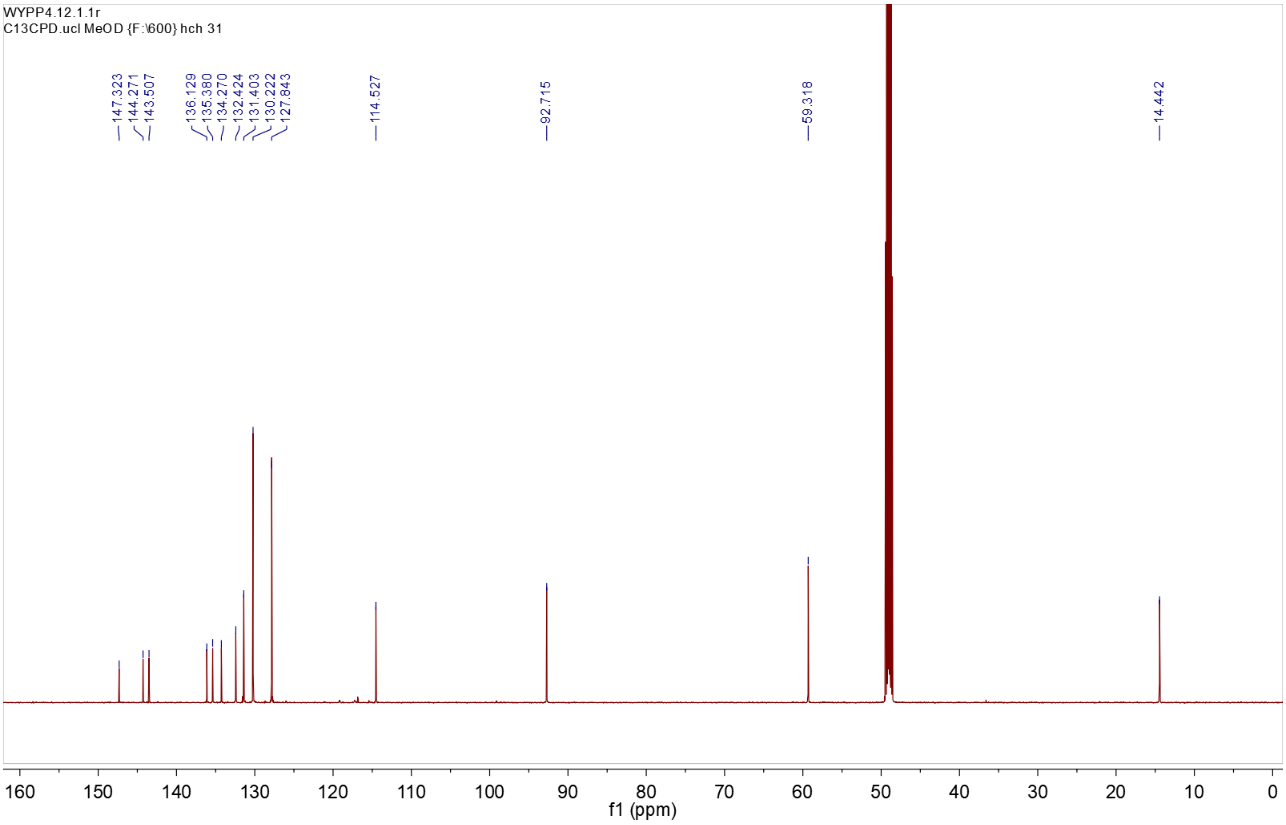

3e

^{1}H NMR (600 MHz; CD_3OD)

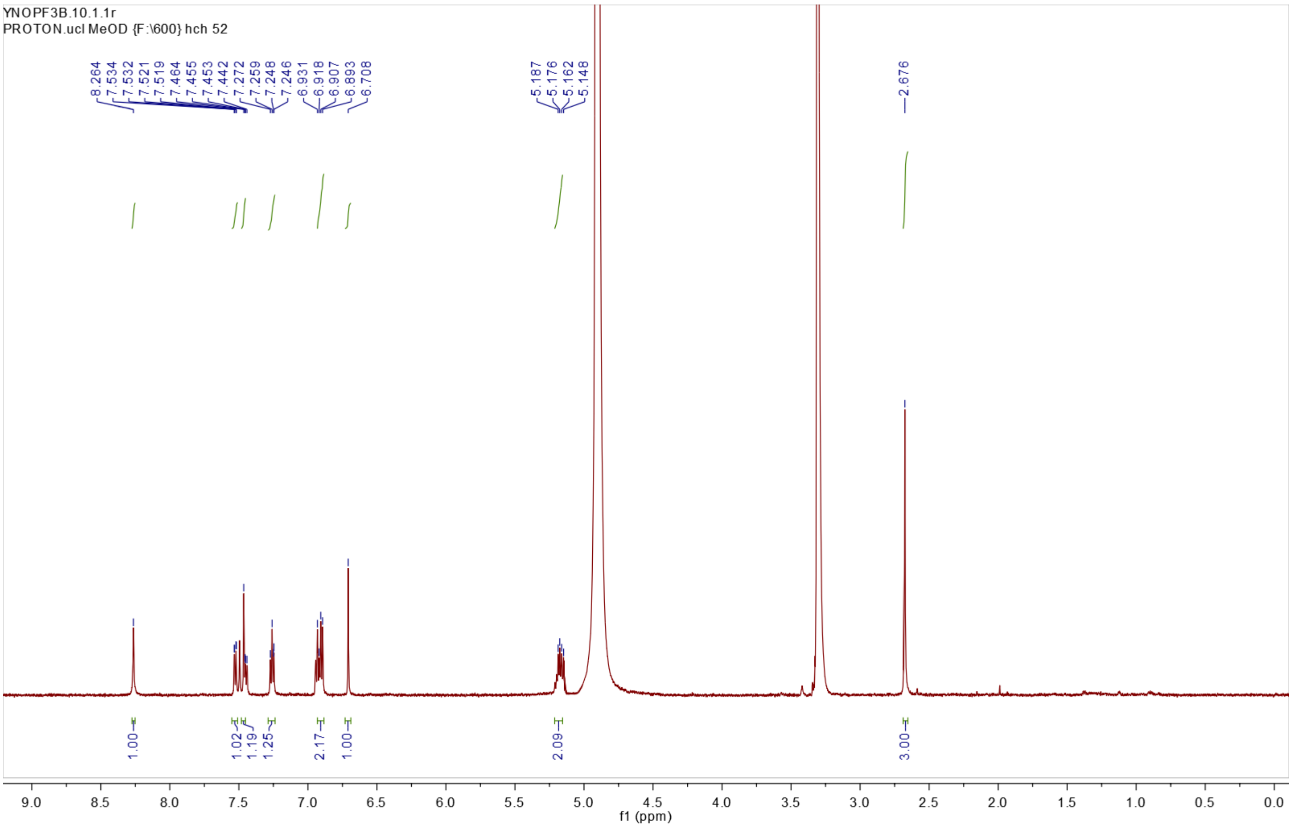

^{13}C NMR (151 MHz; CD_3OD)

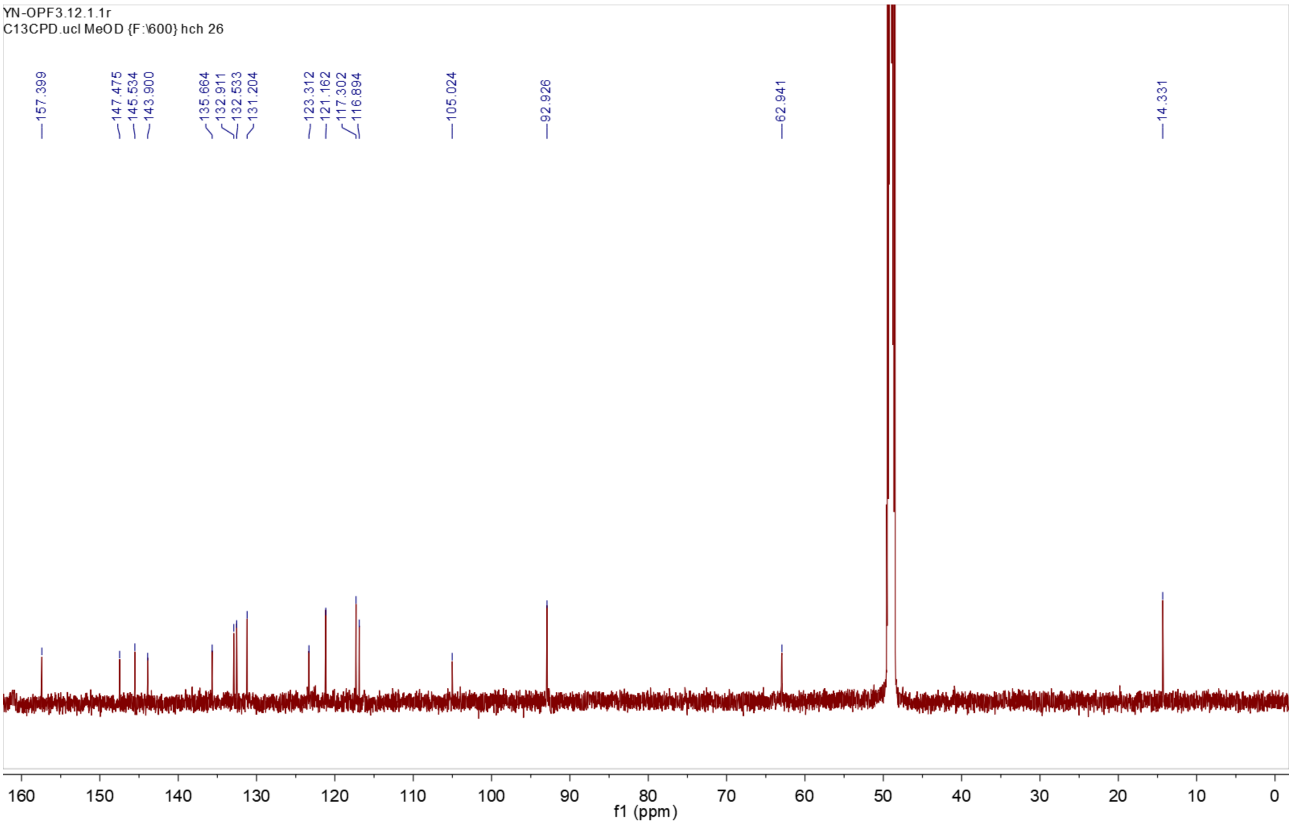

3e Phosphorylated

^{1}H NMR (600 MHz; CD_3OD)

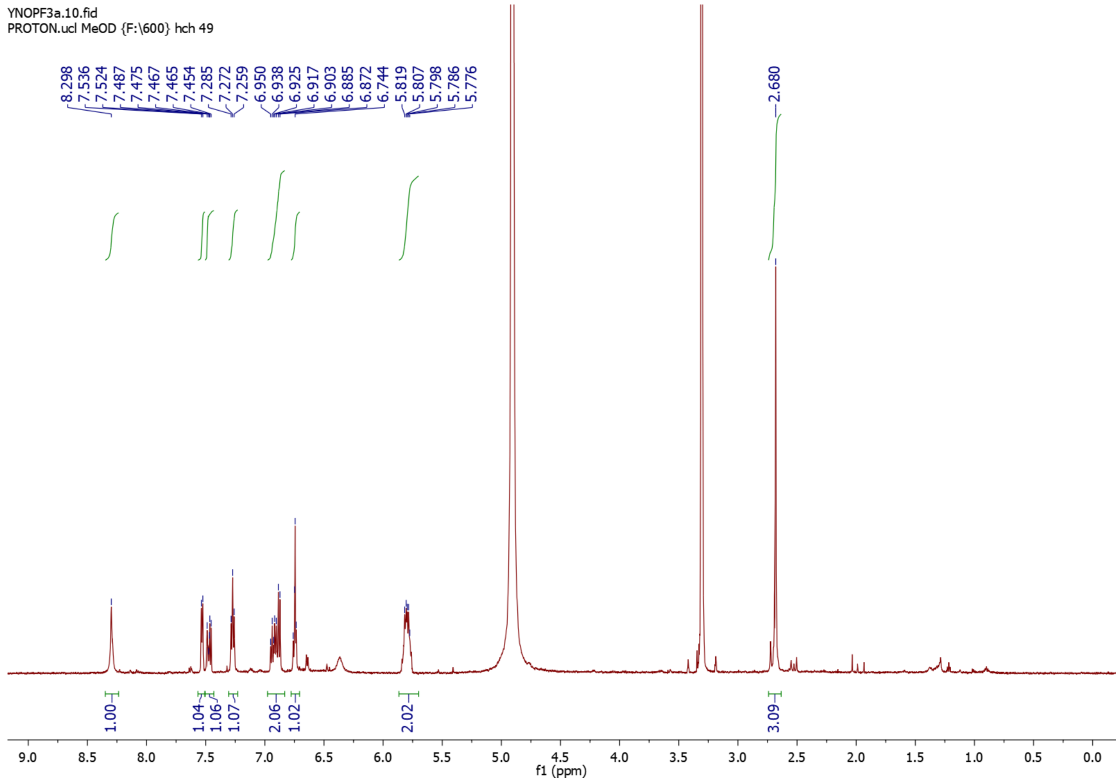

^{13}C NMR (151 MHz; CD_3OD)

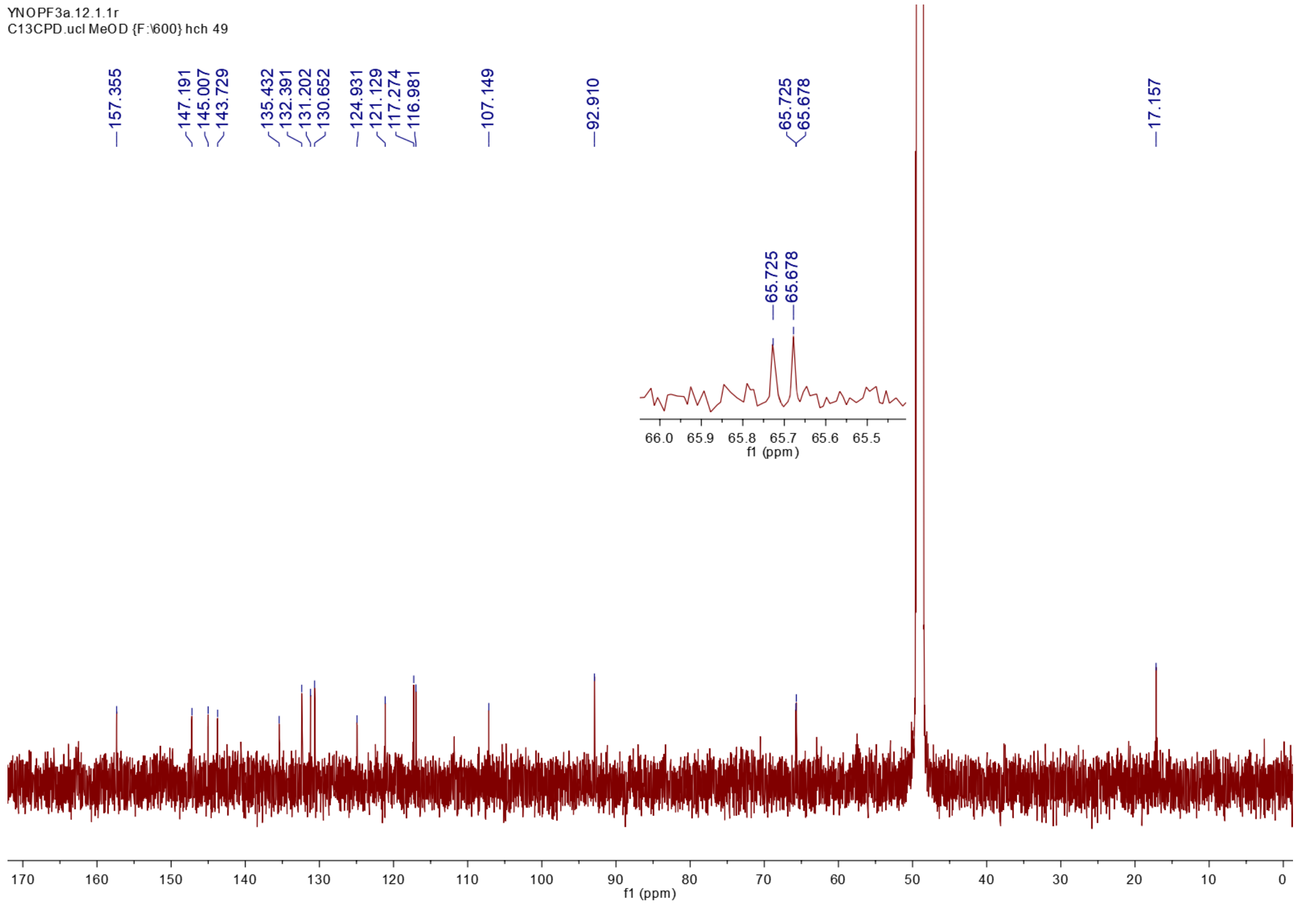

7a

^{1}H NMR (600 MHz; CD_3OD)

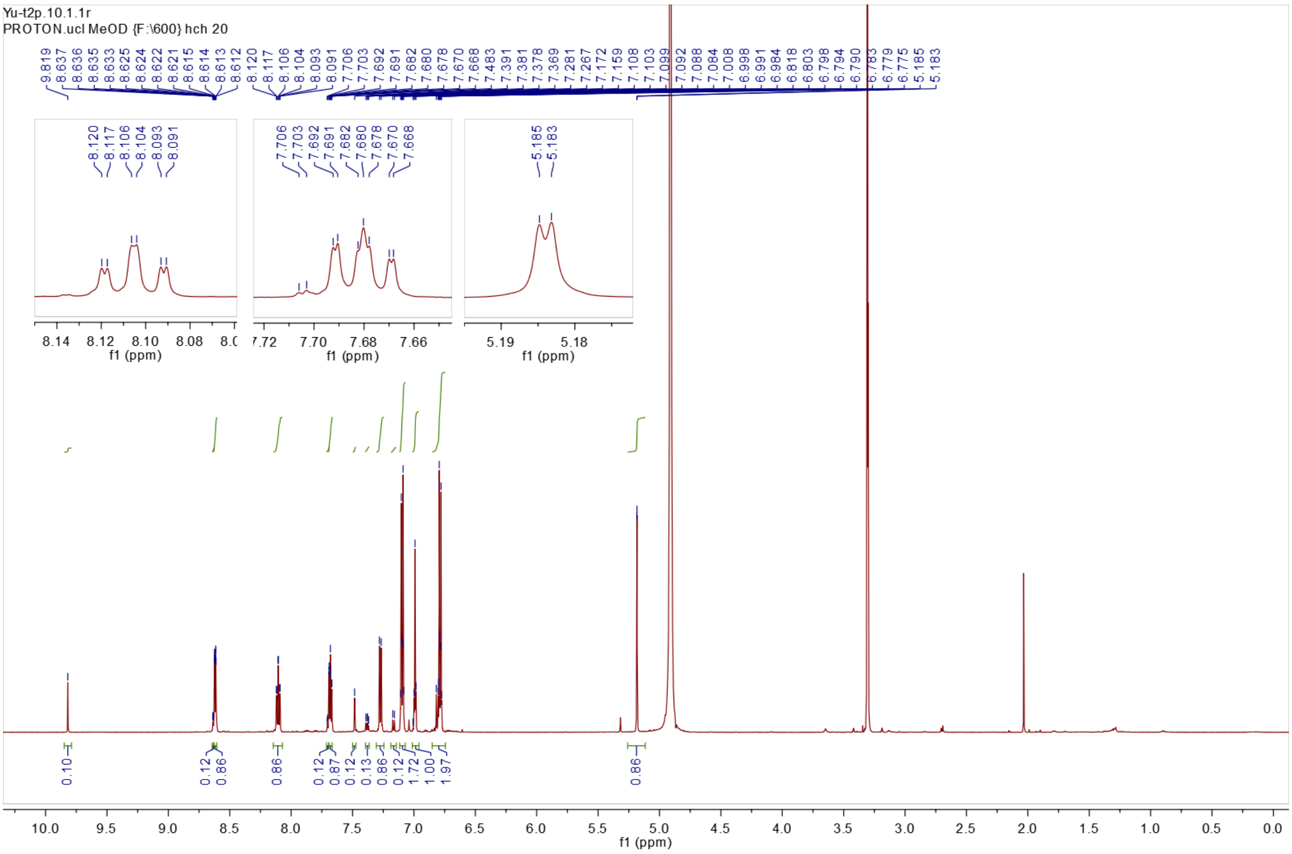

^{13}C NMR (151 MHz; CD_3OD)

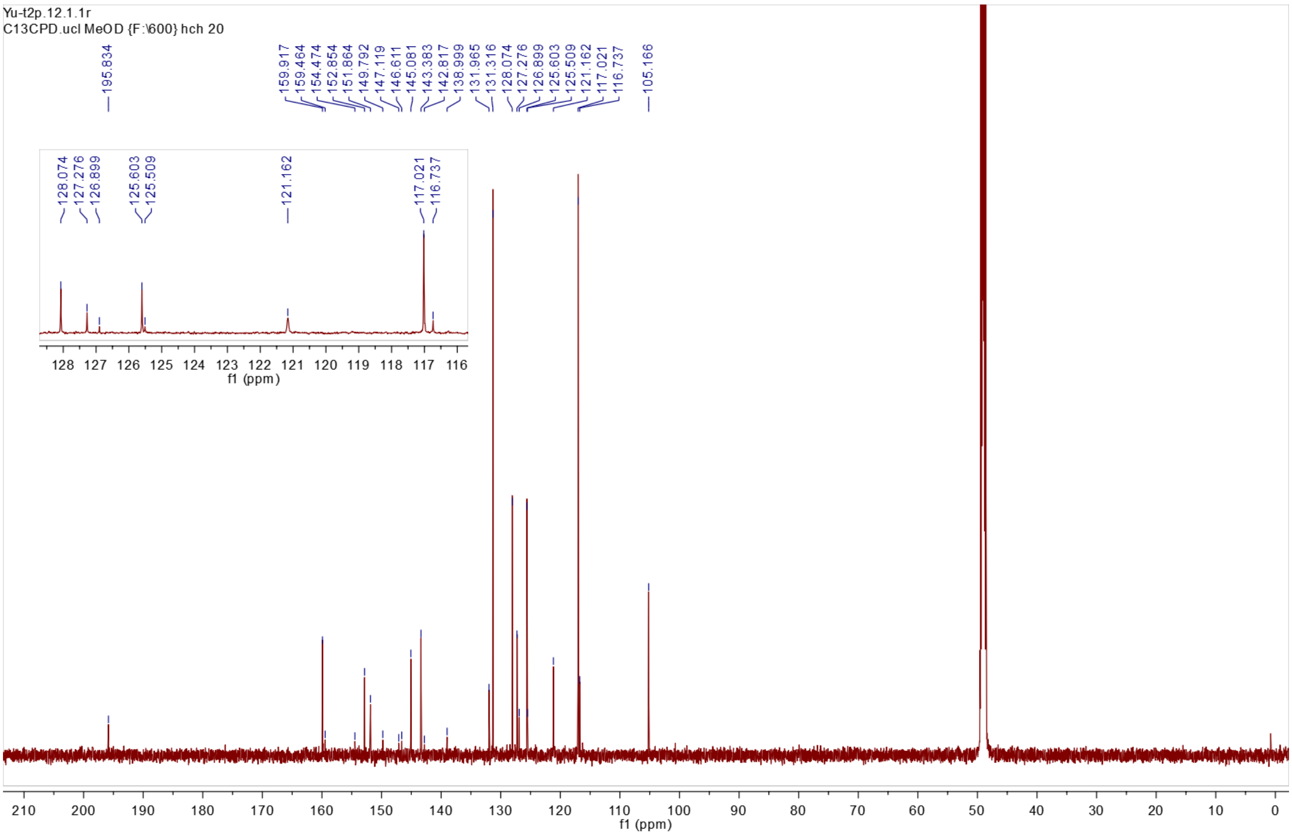

NOSEY NMR (600 MHz; CD_3OD)

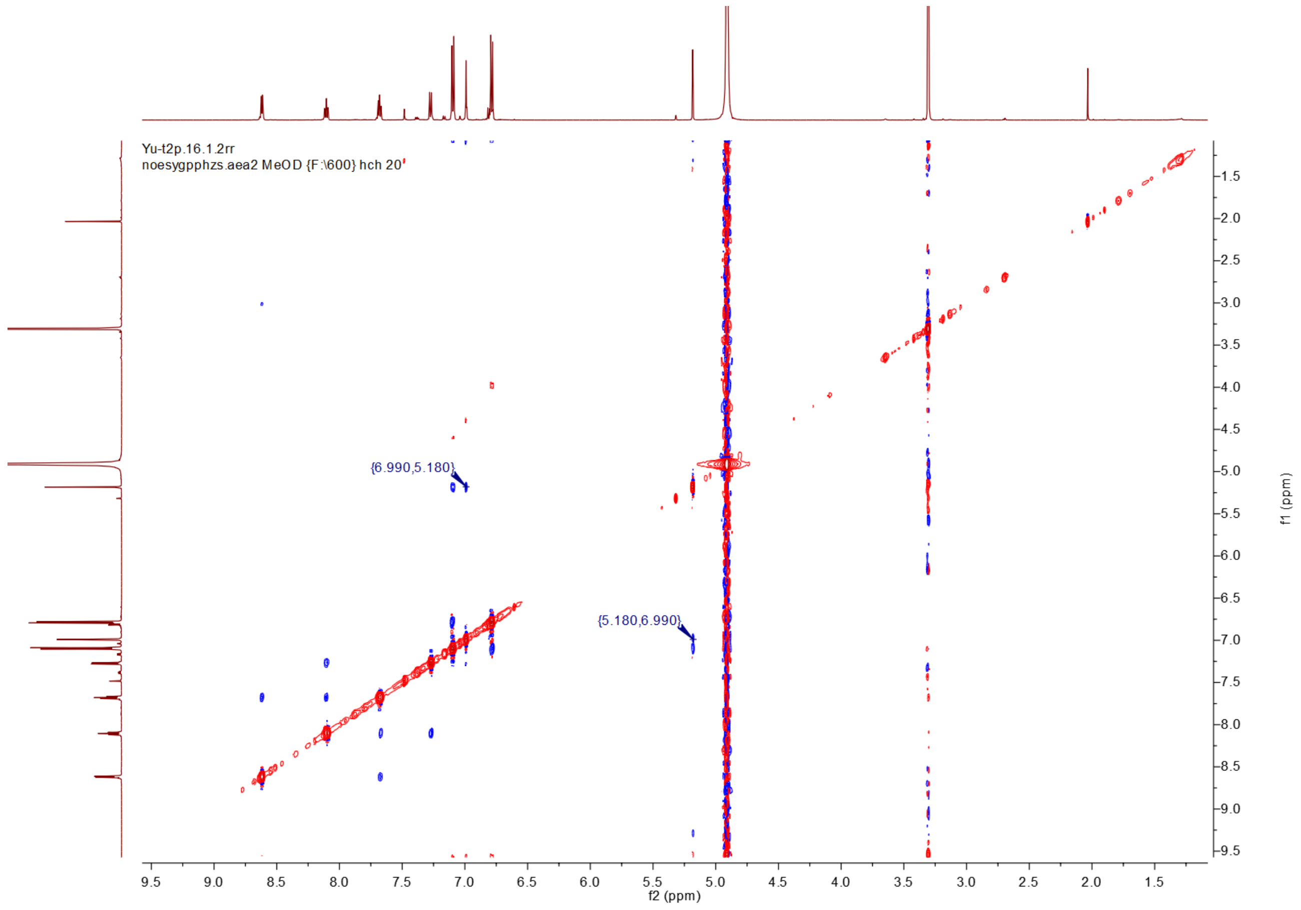

7b

^{1}H NMR (600 MHz; CD_3OD)

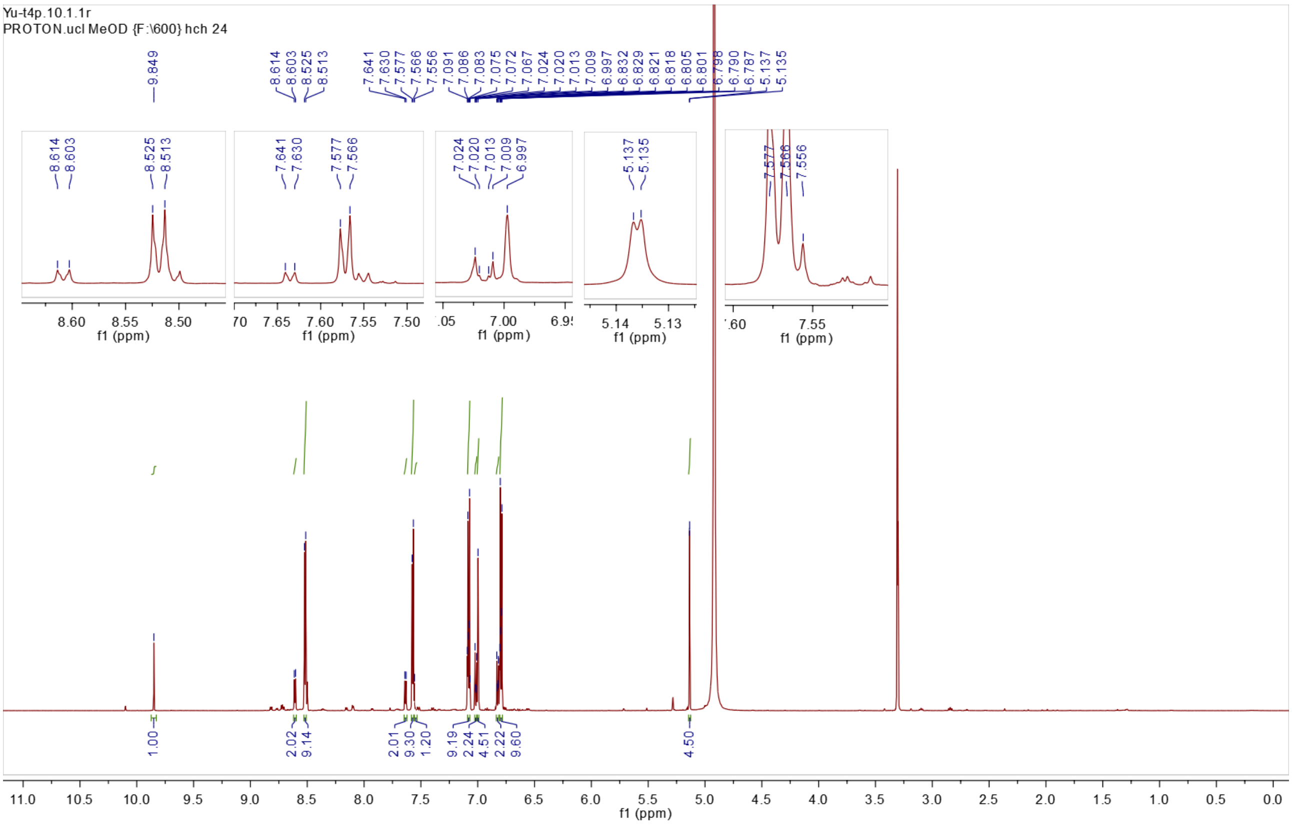

^{13}C NMR (151 MHz; CD_3OD)

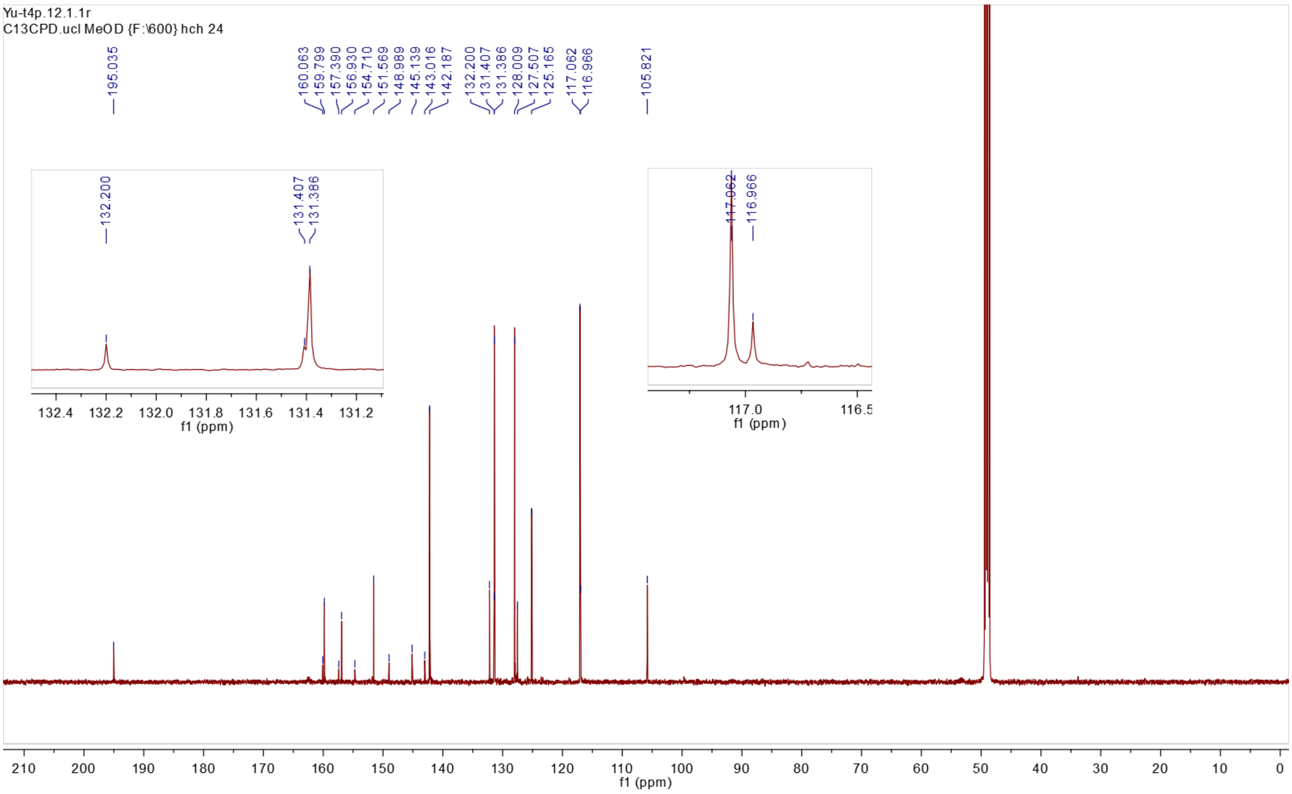

NOSEY NMR (600 MHz; CD_3OD)

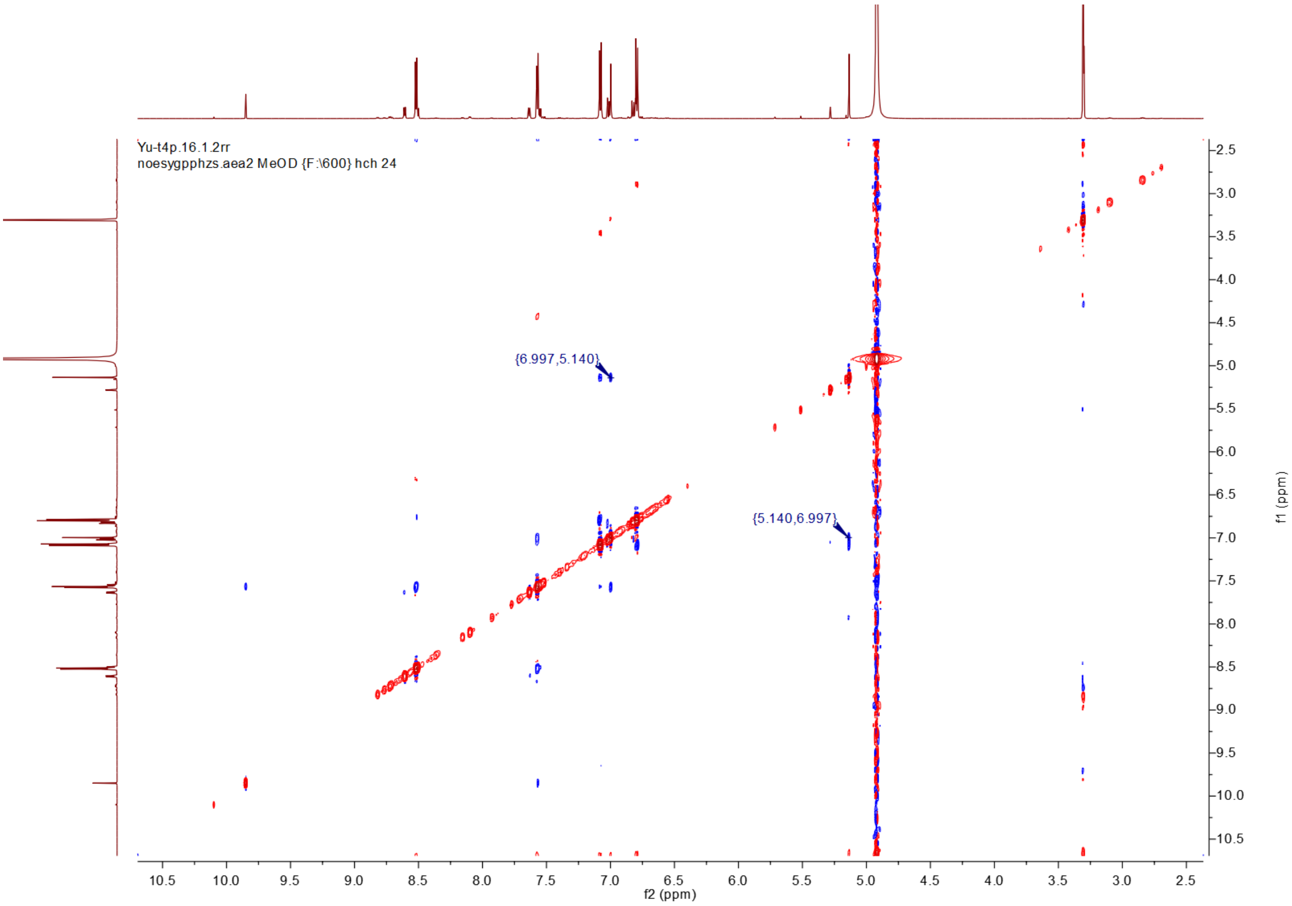

7c

^{1}H NMR (600 MHz; CD_3OD)

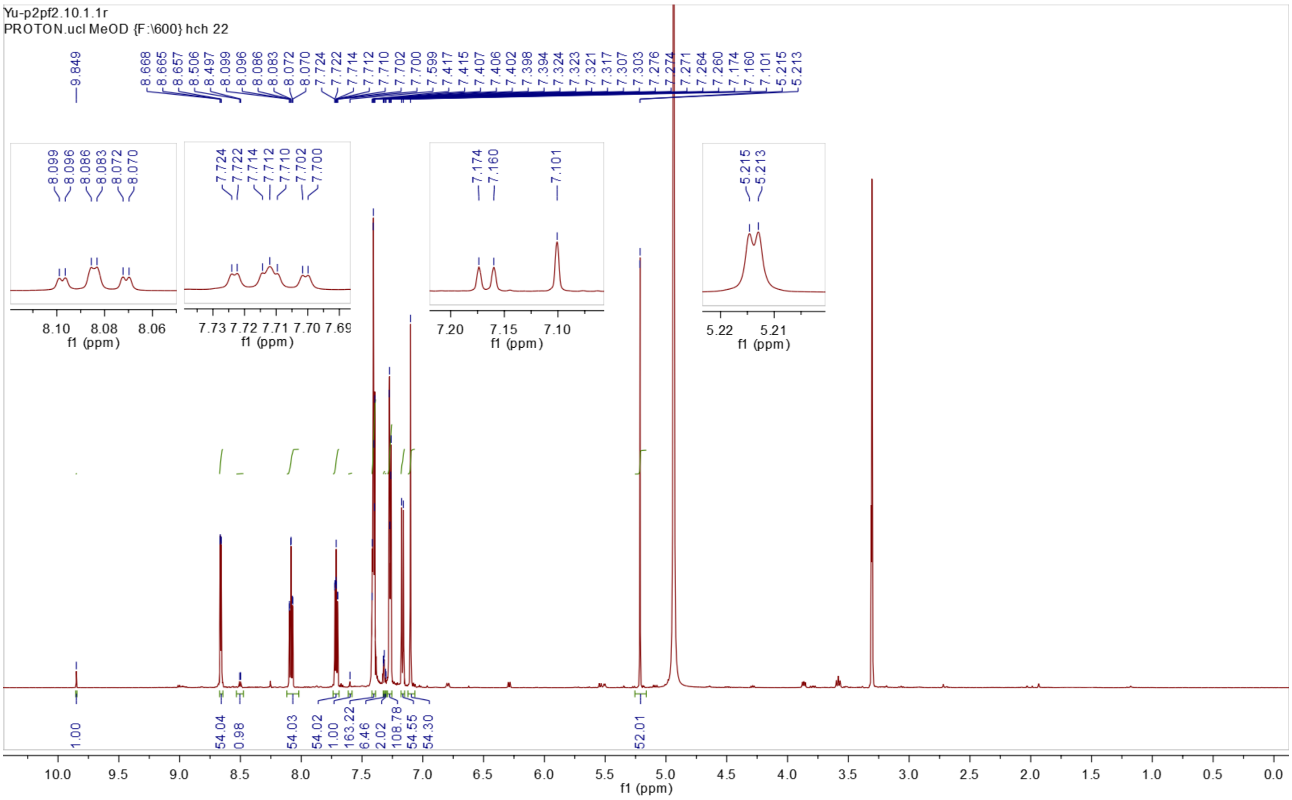

^{13}C NMR (151 MHz; CD_3OD)

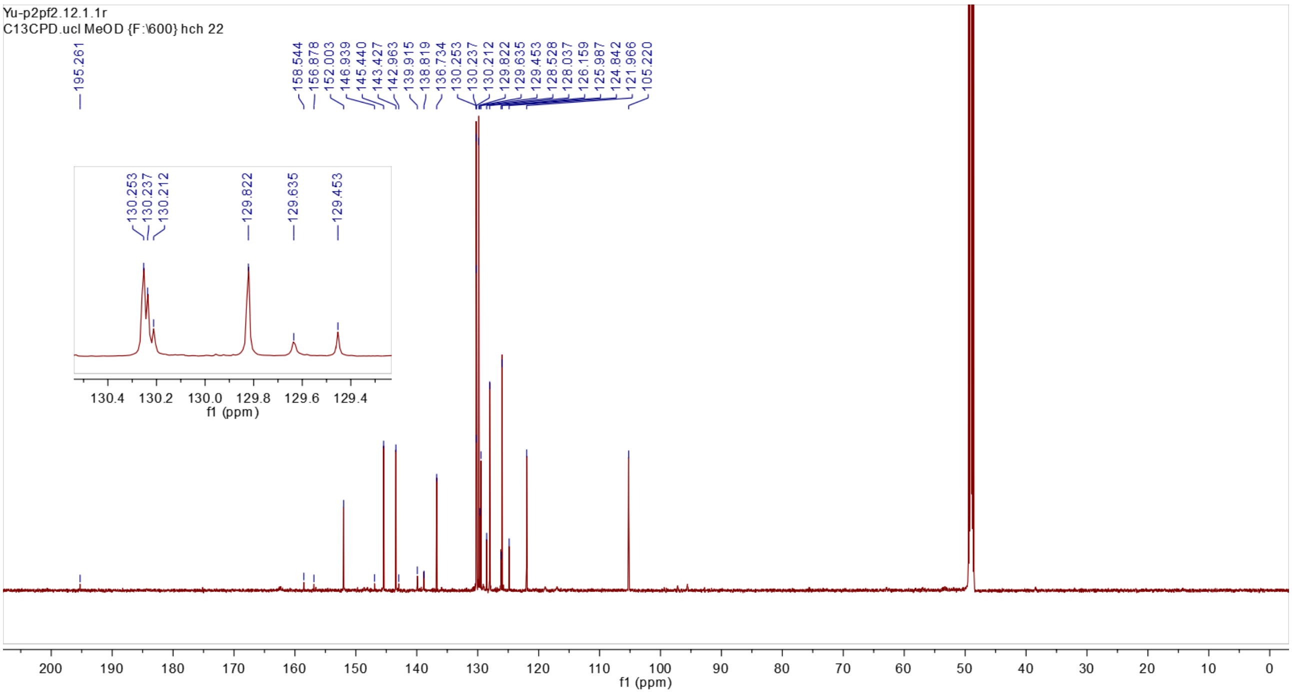

NOSEY NMR (600 MHz; CD_3OD)

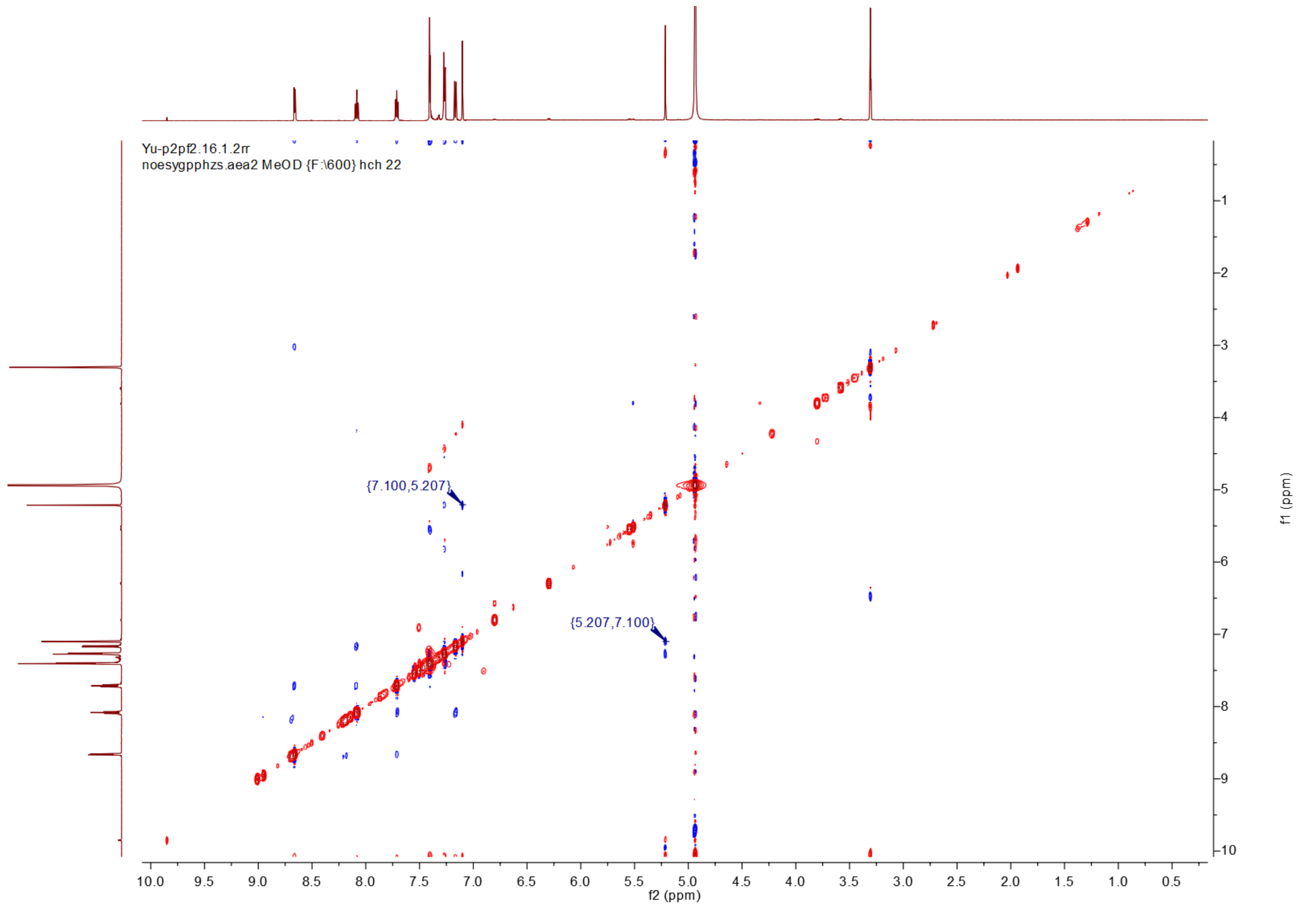

7d

^{1}H NMR (600 MHz; CD_3OD)

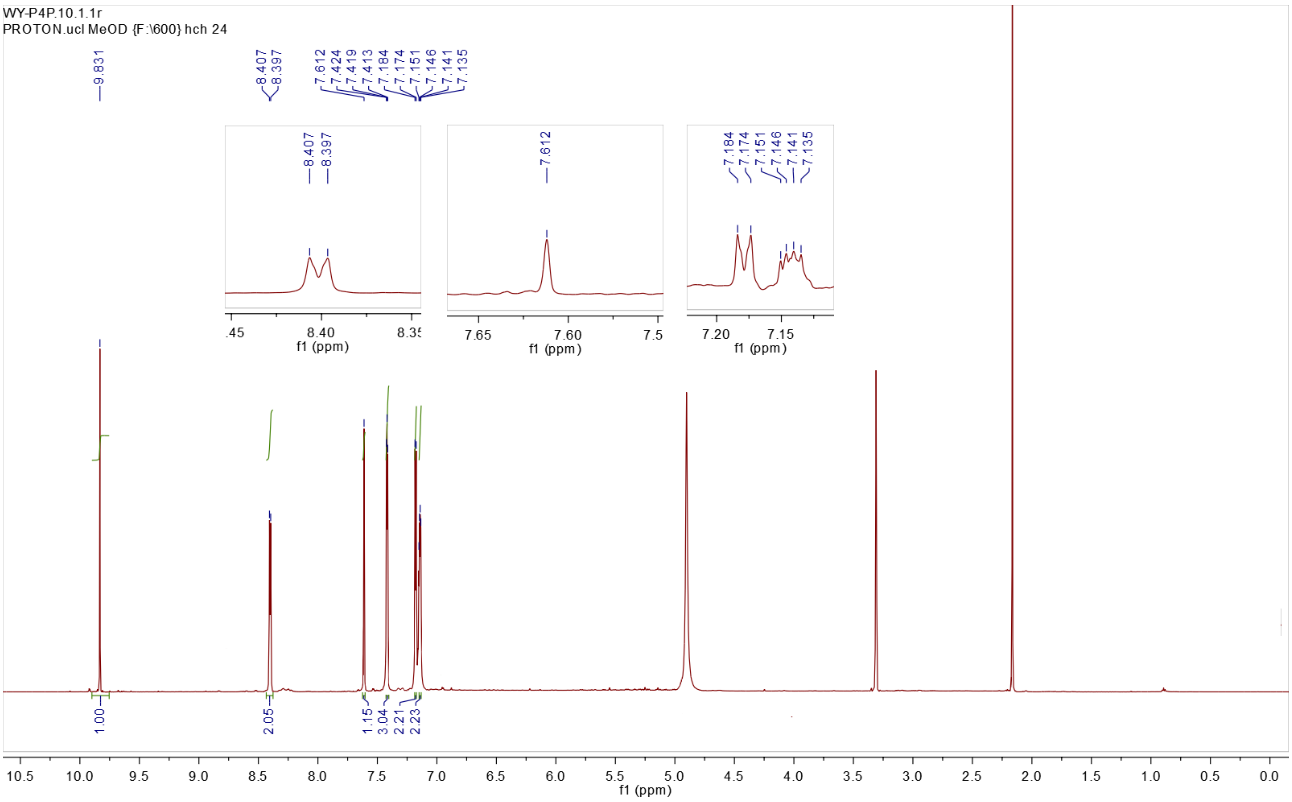

^{13}C NMR (151 MHz; CD_3OD)

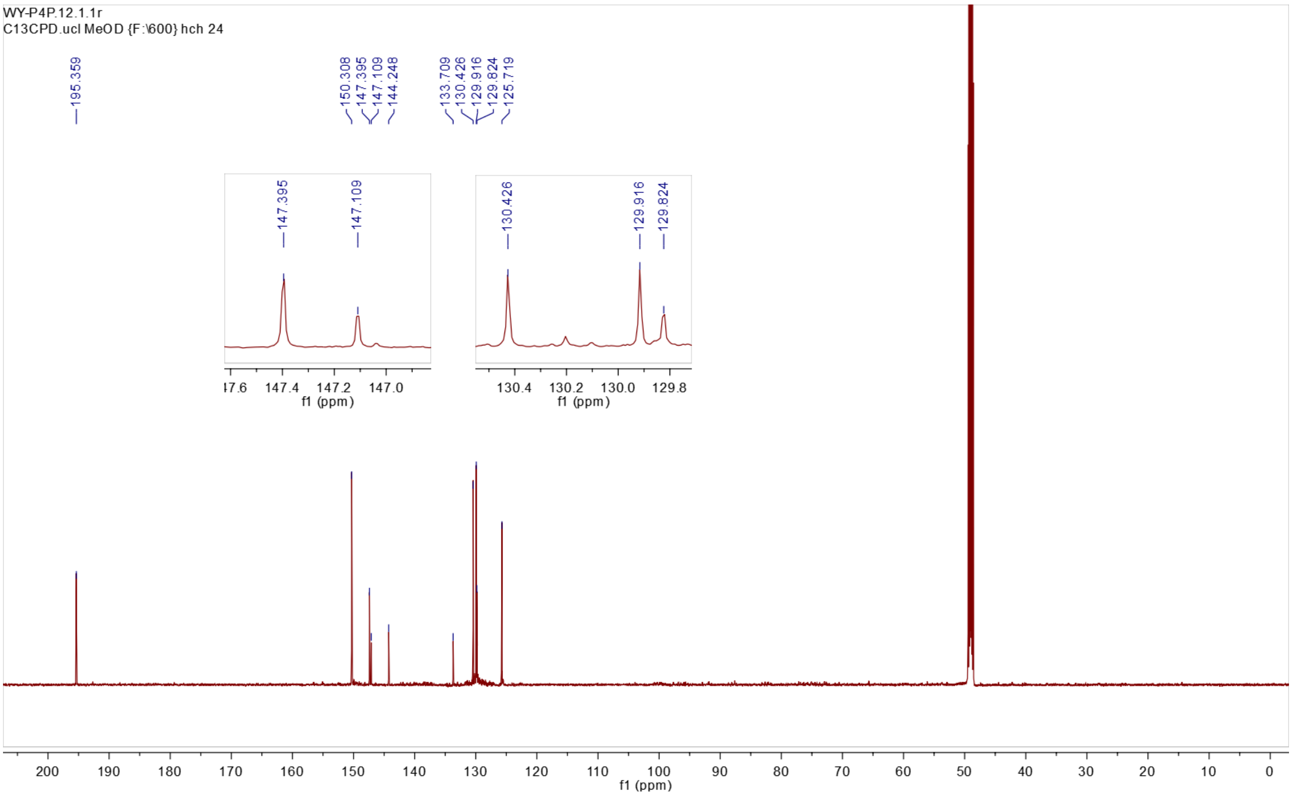

NOSEY NMR (600 MHz; CD_3OD)

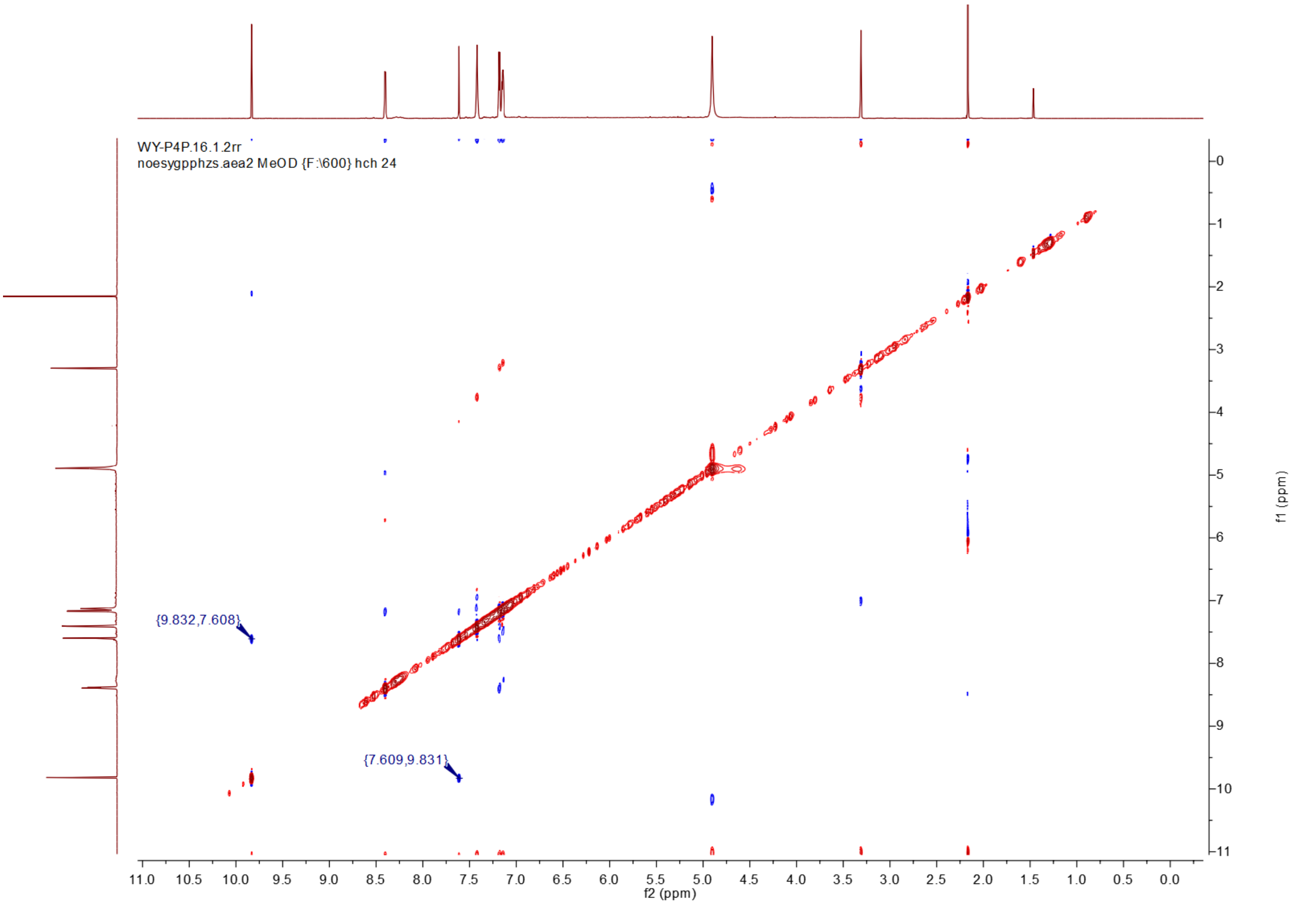

8a

^{1}H NMR (600 MHz; CD_3OD)

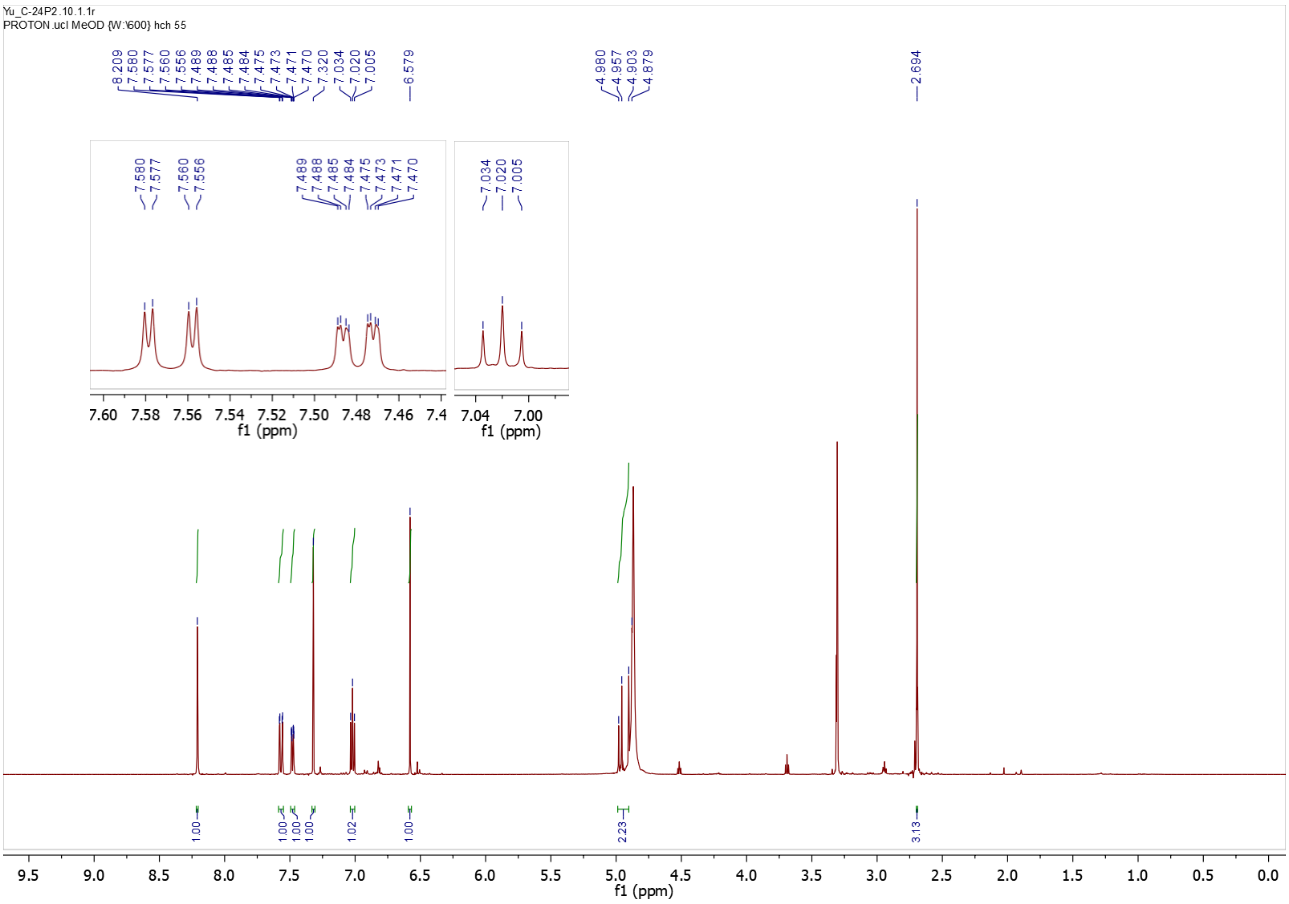

^{13}C NMR (151 MHz; CD_3OD)

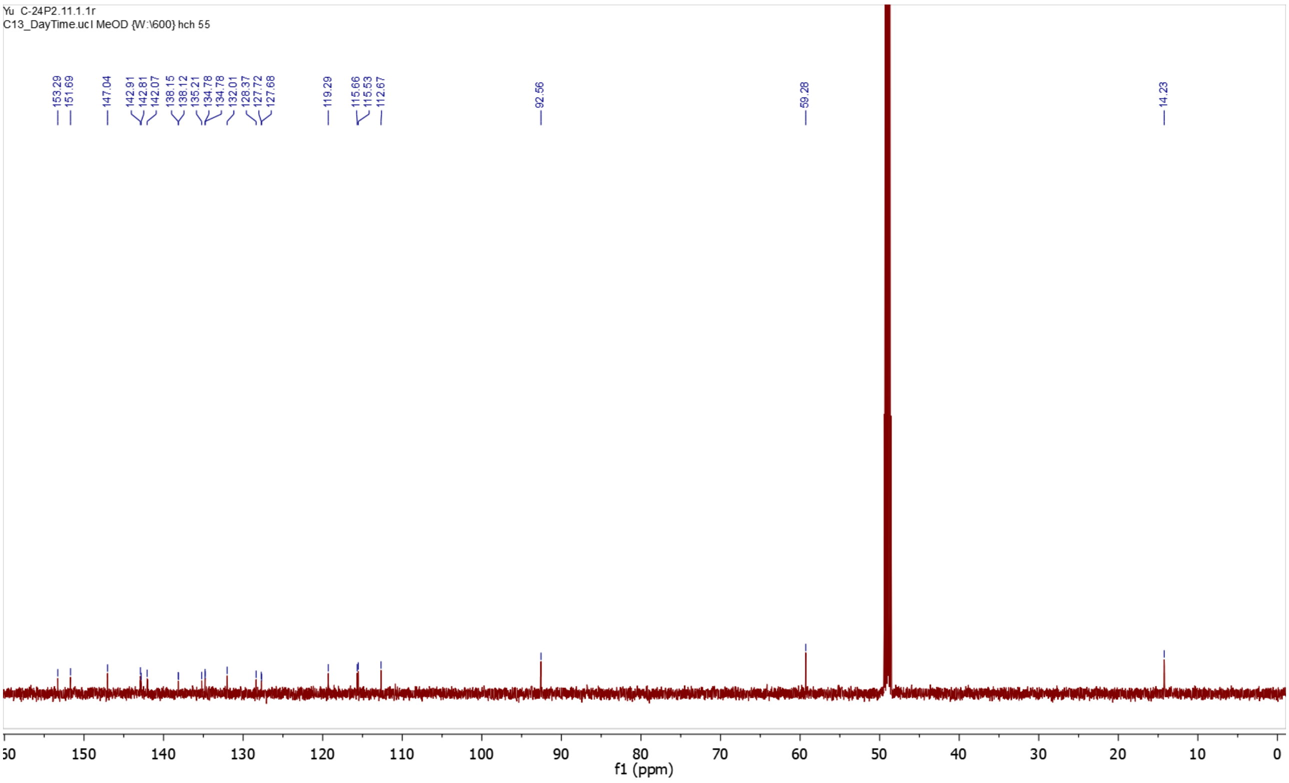

8b

^{1}H NMR (600 MHz; CD_3OD)

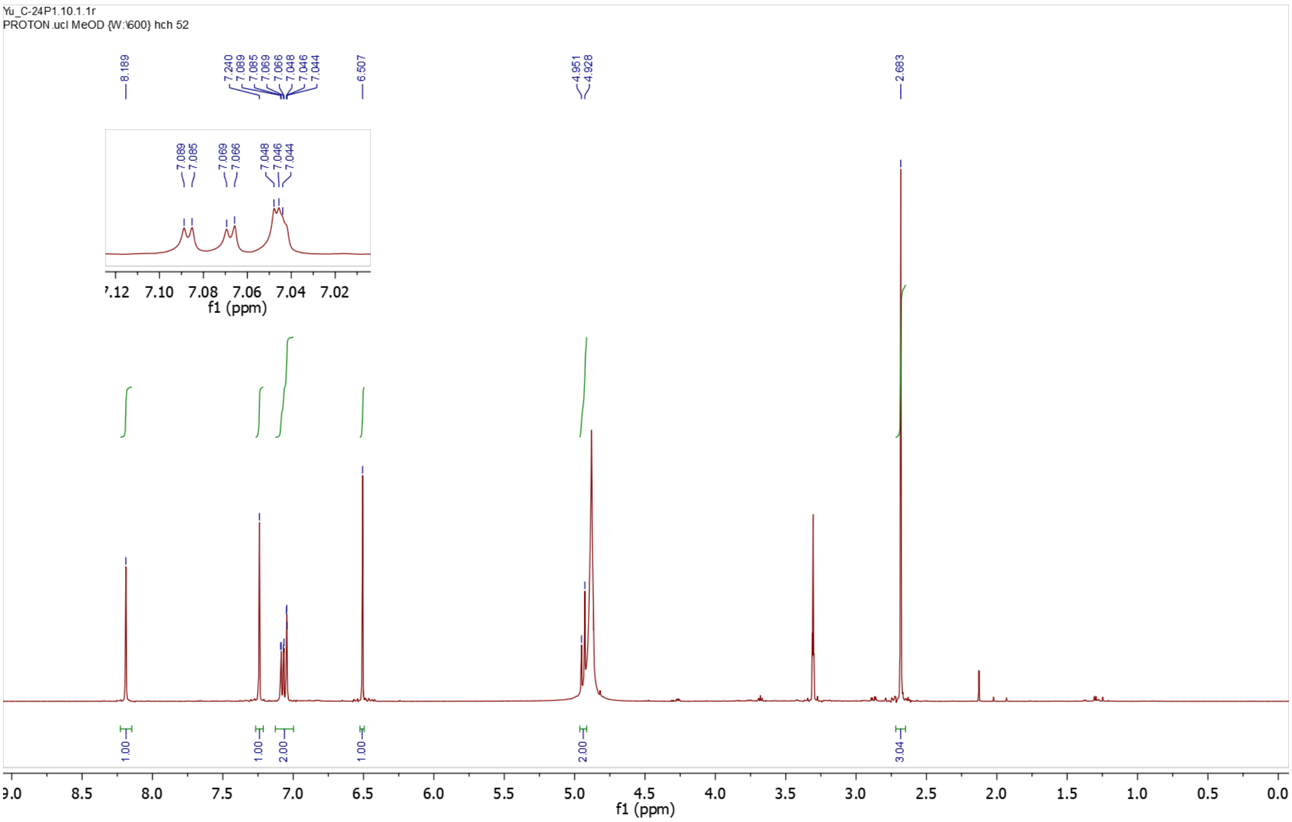

^{13}C NMR (151 MHz; CD_3OD)

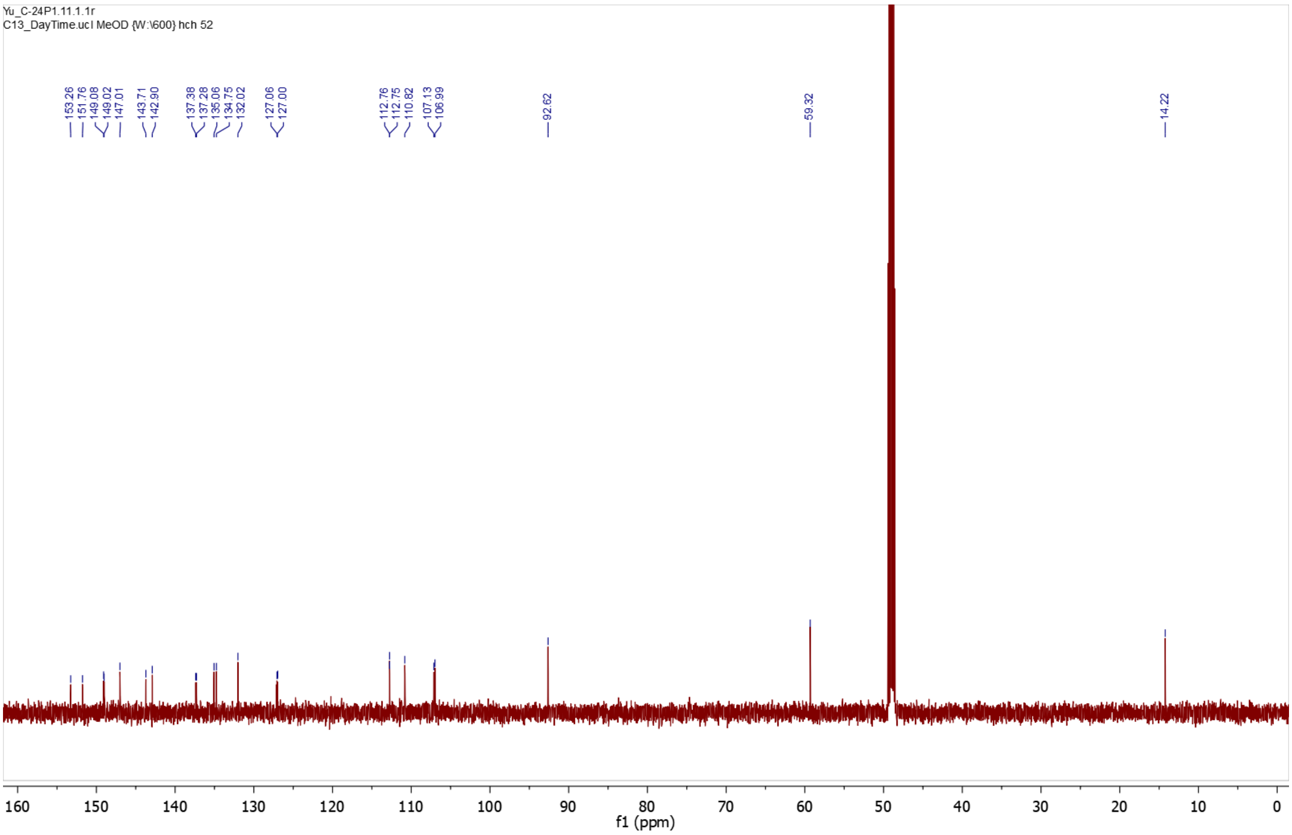

8c

^{1}H NMR (700 MHz; CD_3OD)

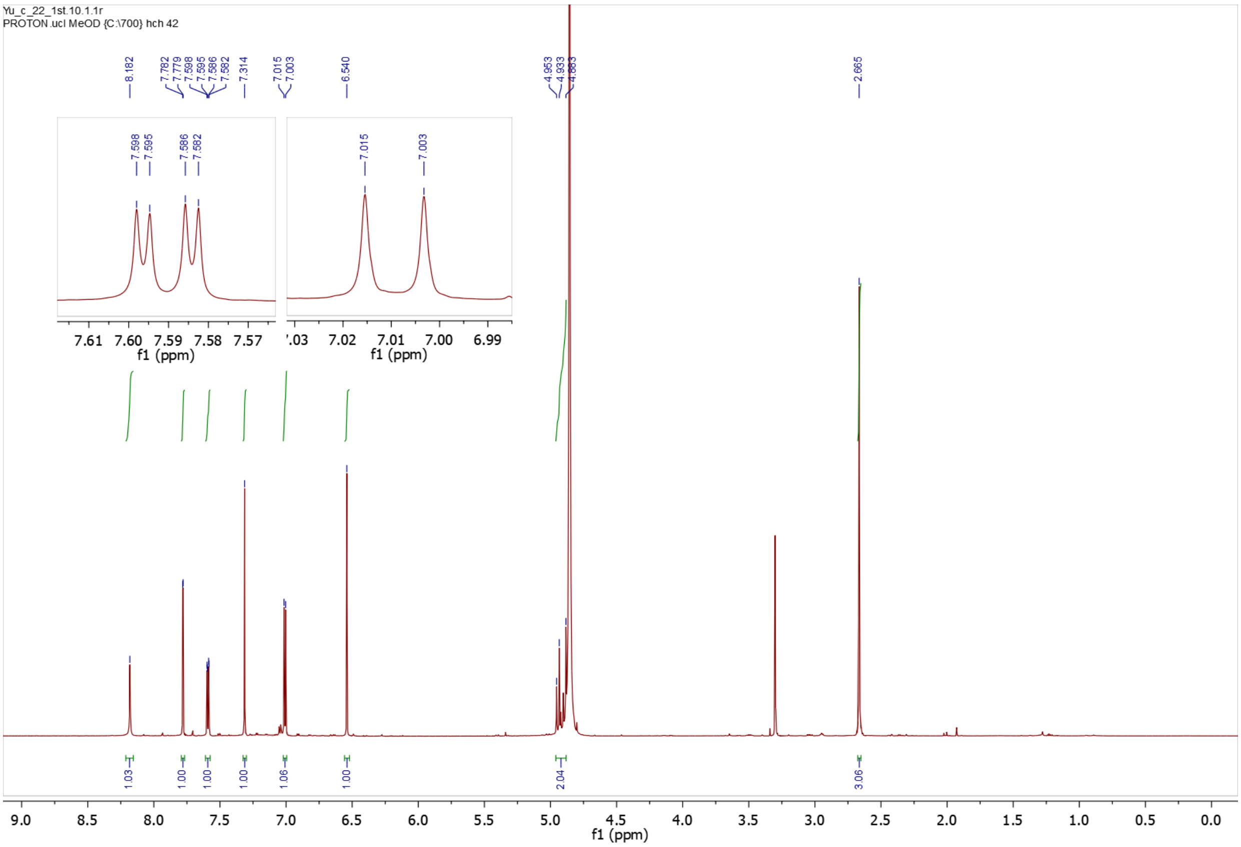

^{13}C NMR (175 MHz; CD_3OD)

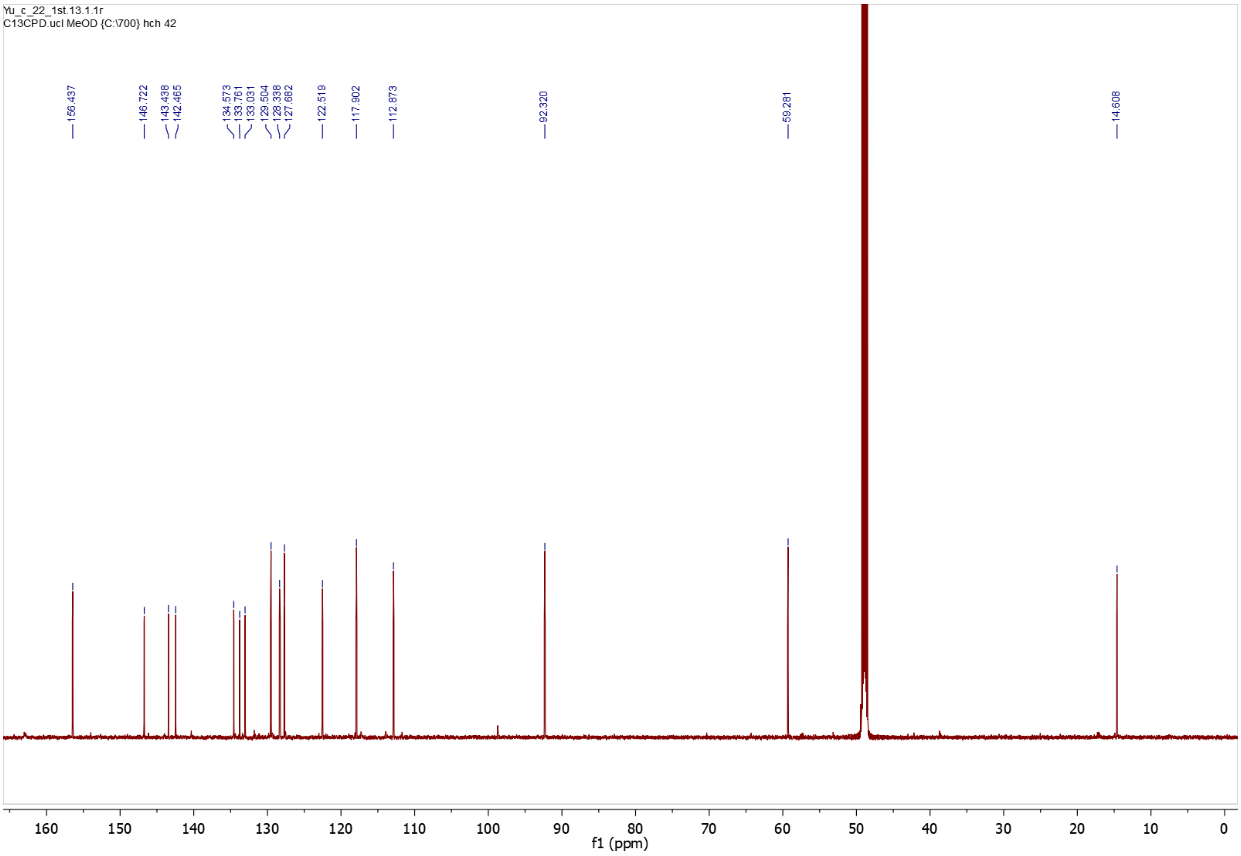

8d

^{1}H NMR (600 MHz; CD$_3$OD)

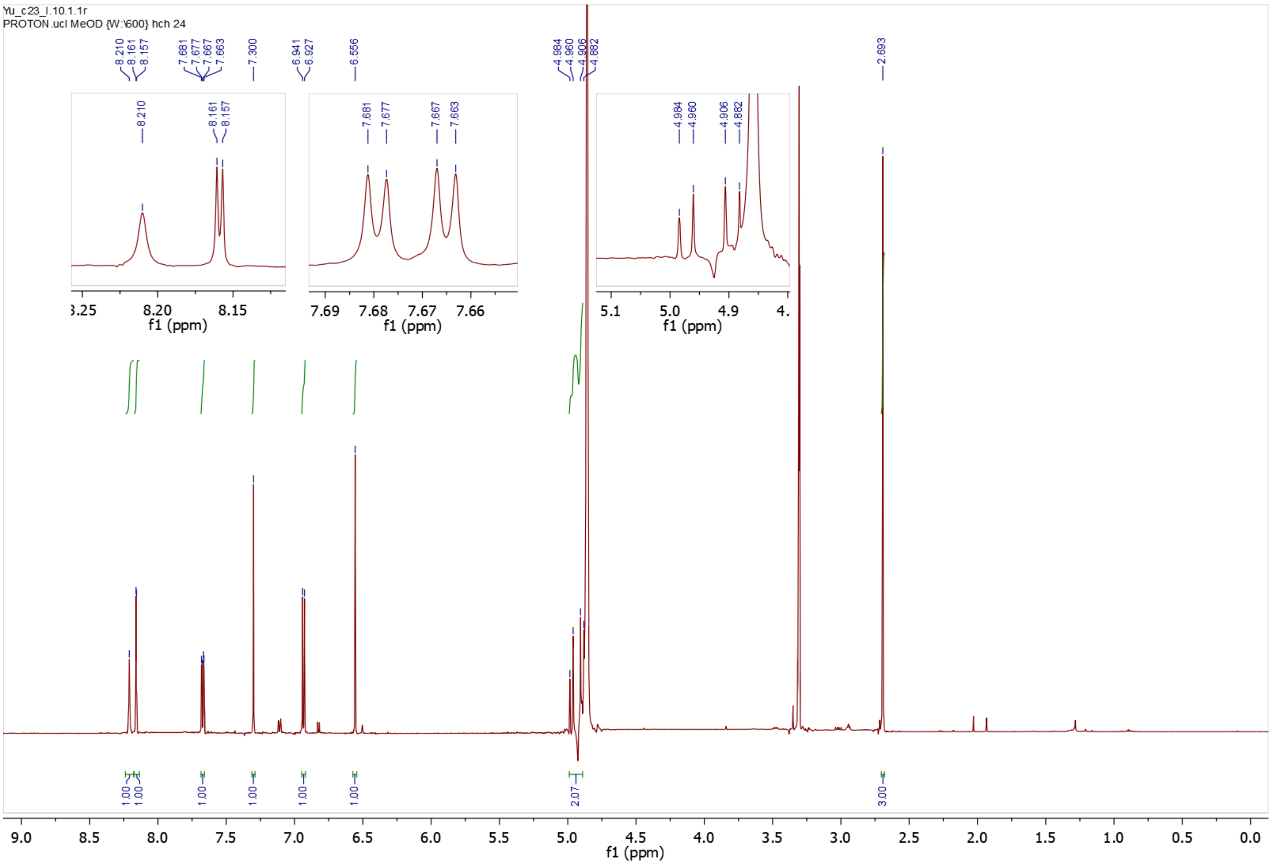

^{13}C NMR (151 MHz; CD$_3$OD)

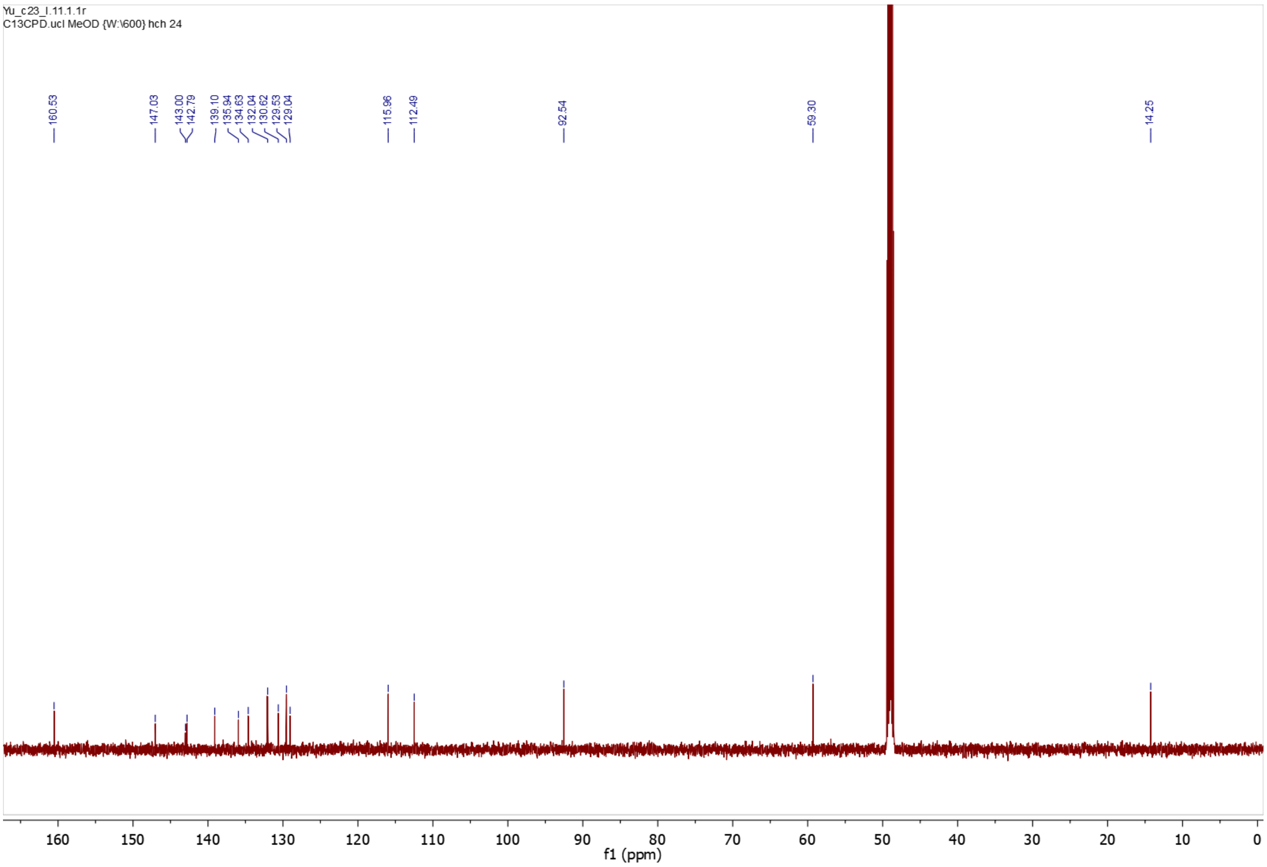

Accurate Mass spectrum data

3a

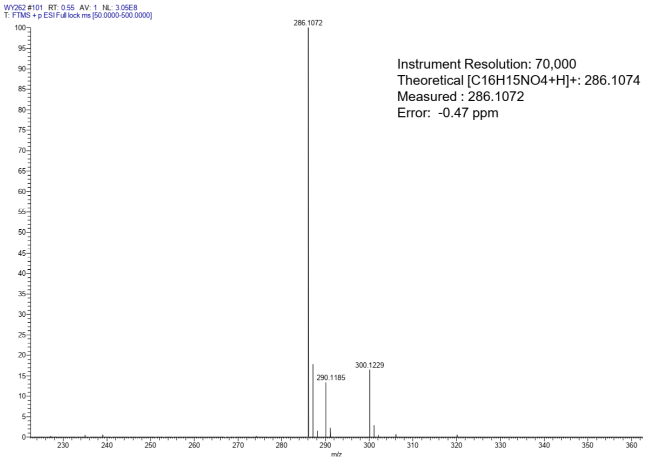

3b

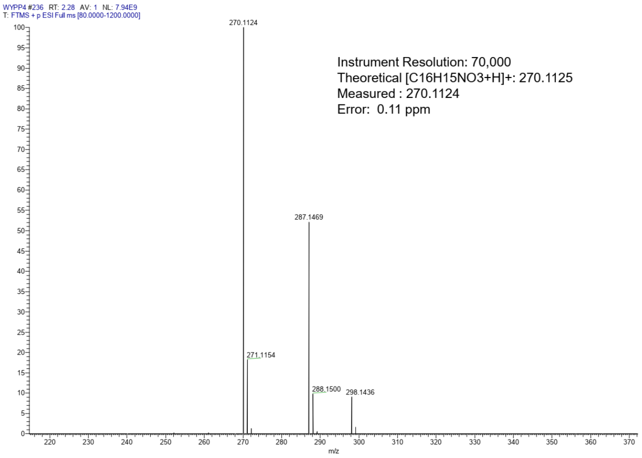

3e

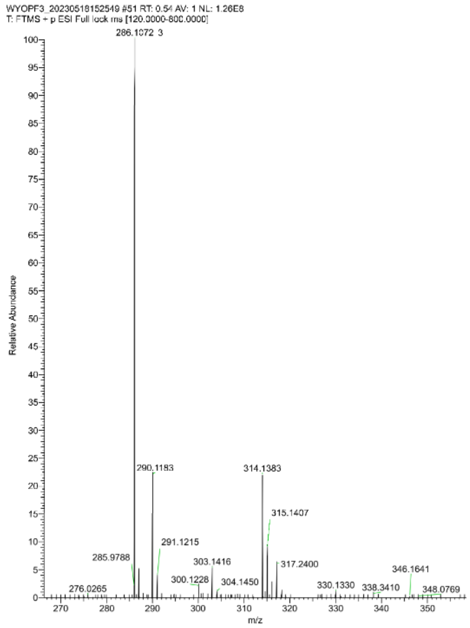

3e Phosphorylated

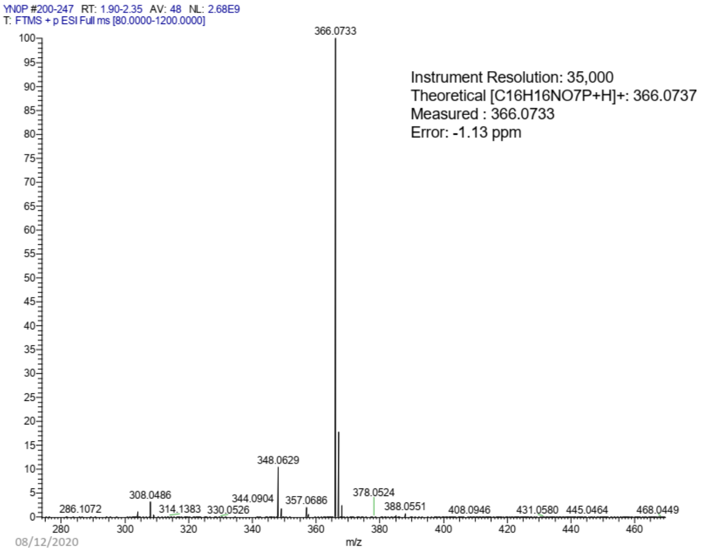

7a

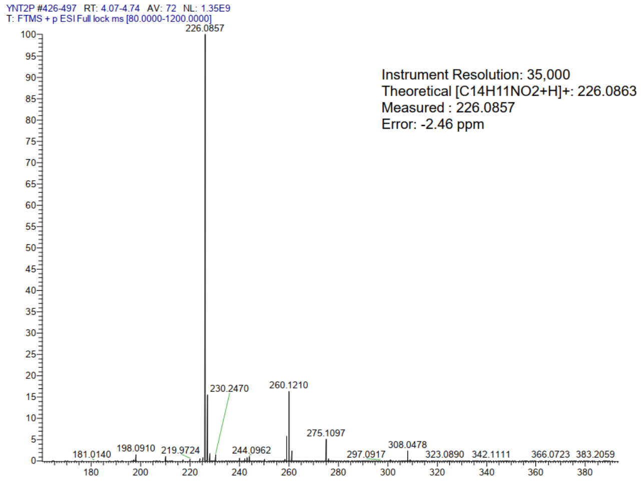

7b

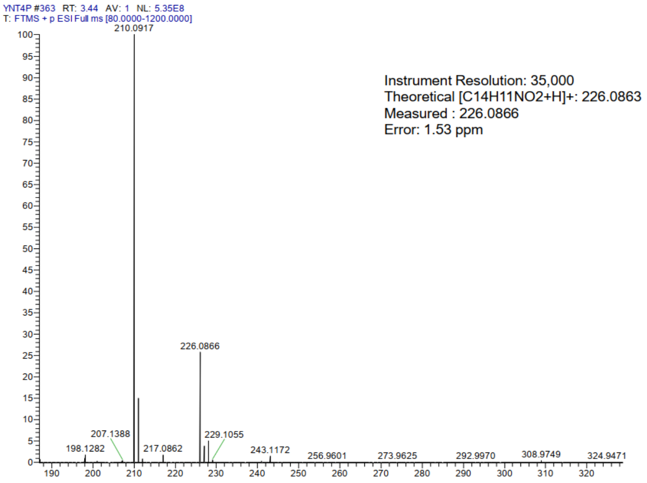

7c

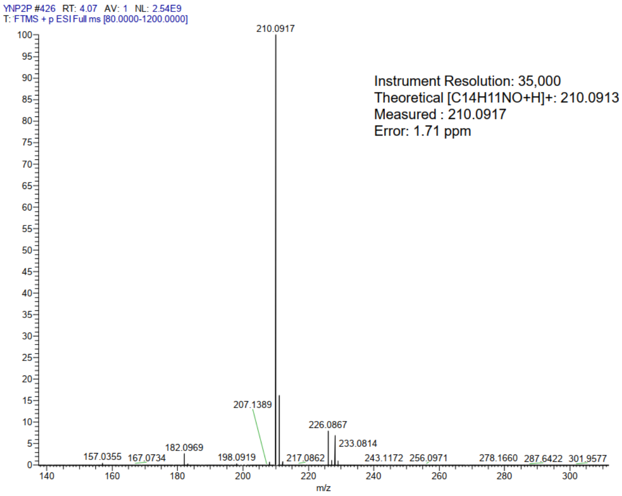

7d

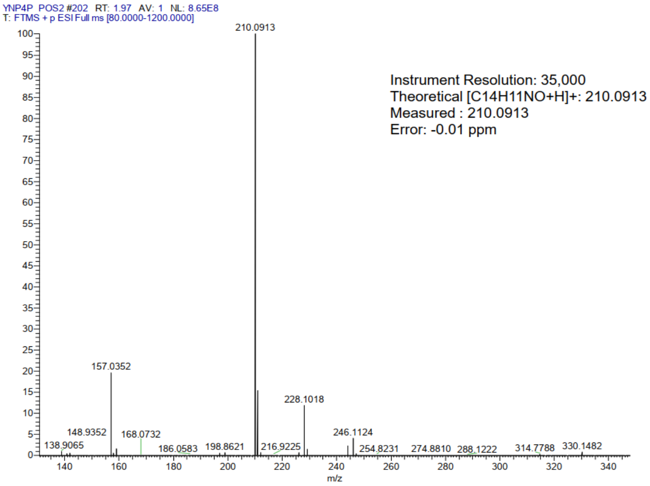

8a

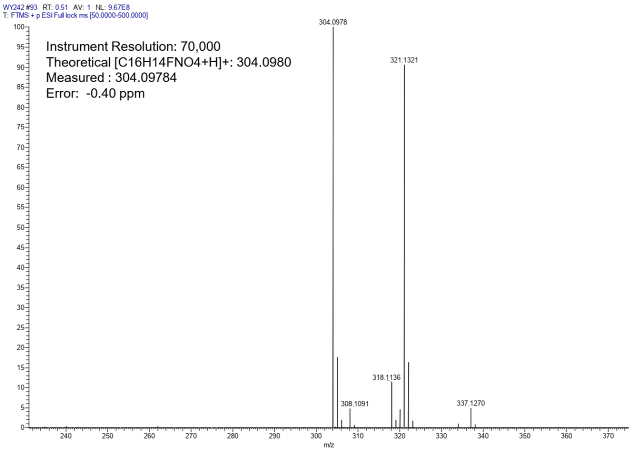

8b

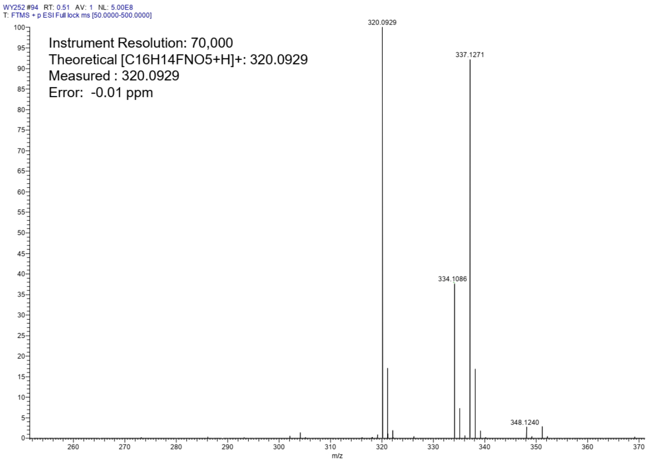

8c

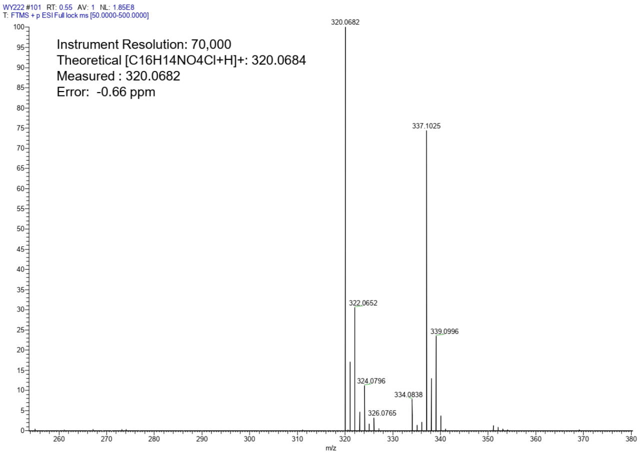

8d

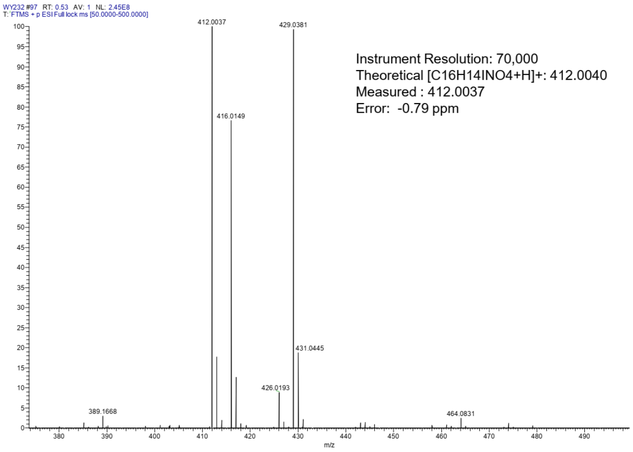

Full LC-MS for P1-P4

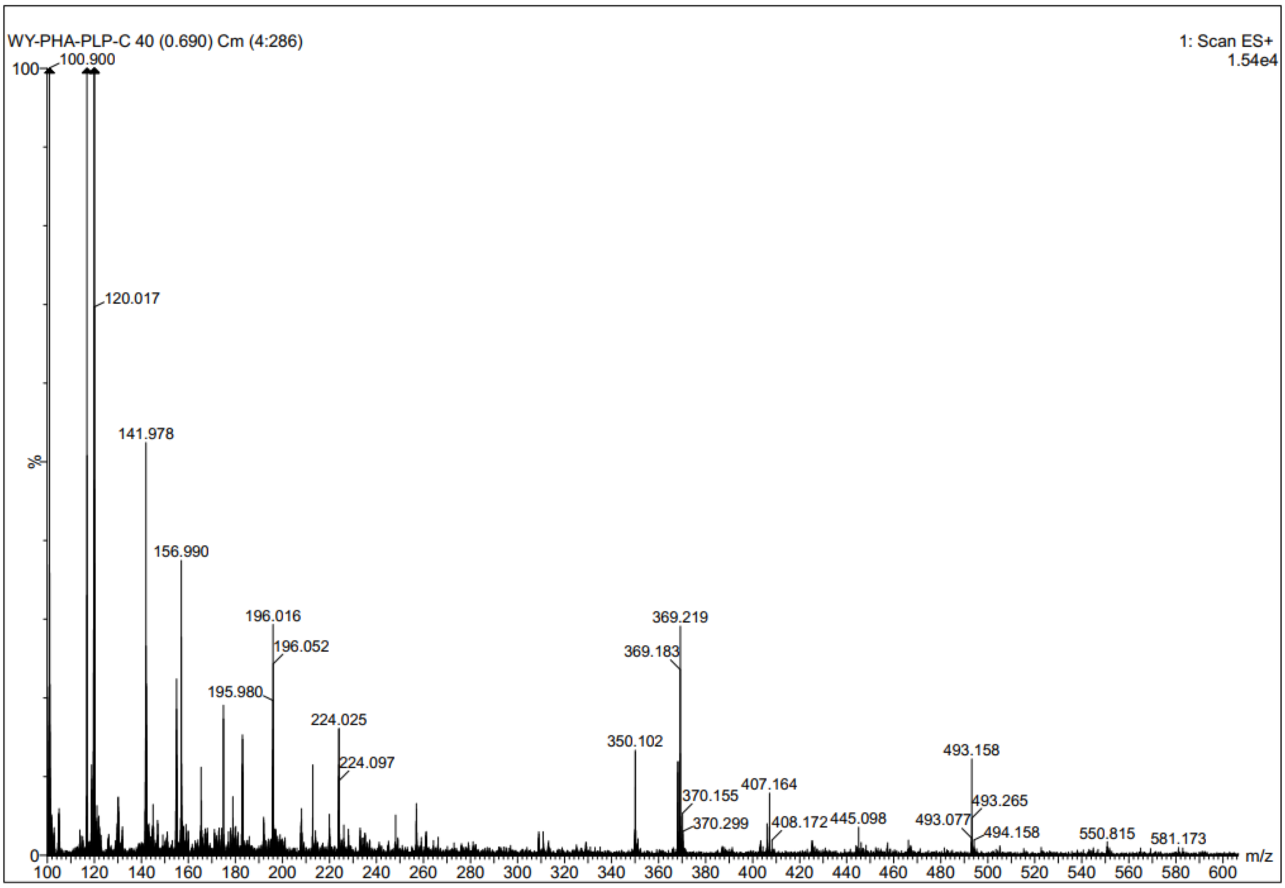

Supplement: CY-014-D3CY01370G-s004 [file CY-014-D3CY01370G-s004.pdf]
